# Supplementary material for: Ecological Prevalence and Non-Enzymatic Formation of Imidazolium Alkaloids on Moon Snail Egg Collars
Source: Molecules. 2026 Jan 1;31(1):159. doi: 10.3390/molecules31010159 (PMC12787093; doi:10.3390/molecules31010159)
Supplement: Supplementary file 1 [file molecules-31-00159-s001.zip › molecules-4035260-supplementary.pdf]

Supporting Information

Ecological Prevalence and Non-Enzymatic Formation of Imidazolium Alkaloids on Moon Snail Egg Collars

Karla Piedl,[1] Caitlyn O. Agee,[2] Anthony G. Tarulli,[1] Rose Campbell,[1] Paige Banks,[1] Nicklas W. Buchbinder,[1] R. Thomas Williamson,[2] Emily Mevers[1,*]

[1]Department of Chemistry, Virginia Tech, Blacksburg, VA, 24061, USA

[2]Department of Chemistry and Biochemistry, University of North Carolina – Wilmington, Wilmington, NC, 28403, USA

*Email: emevers@vt.edu; phone: 540-231-6570

Table of Contents

Supplementary Figures ....pg. 4
Figure S1. Intruder assays between *Flavobacteriaceae* that exhibited growth inhibition ...pg. 4
Figure S2. Spot-on-lawn assay of *C. omniverscoria* EM610 flash chromatography fractions ....pg. 4
Figure S3. LCMS chromatogram of EM610C, where bacillimidazoles A (**1**) and E (**2**) are the major metabolites ....pg. 5
Figure S4. LCMS chromatogram of flash chromatography fractions showing bacillimidazoles A (**1**) and E (**2**) are majorly present in fraction D ....pg. 6
Figure S5. HPLC chromatogram of purified bacillimidazole A (**1**)....................pg. 6
Figure S6. HPLC chromatogram of purified bacillimidazole B (**2**)....................pg. 7
Figure S7. ^{1}H NMR spectrum (500 MHz; d_6-DMSO) of bacillimidazole a (**1**) at two concentrations and with increasing amounts of fuming TFA ....pg. 7
Figure S8. ^{1}H NMR spectra (500 MHz; d_6-DMSO) of bacillimidazole A (**1**). ....pg. 8
Figure S9. ^{13}C NMR spectra (125 MHz; d_6-DMSO) of bacillimidazole A (**1**). ....pg. 8
Figure S10. COSY spectra (^{1}H 500 MHz; d_6-DMSO) of bacillimidazole A (**1**) ....pg. 9
Figure S11. ^{1}H,^{13}C-HMBC spectra (^{1}H 500 MHz; d_6-DMSO) of bacillimidazole A (**1**) ....pg. 9
Figure S12. ^{1}H,^{15}N-HMBC spectra (^{1}H 500 MHz; d_6-DMSO) of bacillimidazole A (**1**) ..pg. 10
Figure S13. HSQC spectra (^{1}H 500 MHz; d_6-DMSO) of bacillimidazole A (**1**) ....pg. 10
Figure S14. HR-LCMS (ESI q-ToF) fragmentation of bacillimidazole A (**1**) ....pg. 11
Figure S15. ^{1}H NMR spectra (500 MHz; d_6-DMSO) of bacillimidazole E (**2**). ....pg. 11
Figure S16. ^{13}C NMR spectra (125 MHz; d_6-DMSO) of bacillimidazole E (**2**). ....pg. 12
Figure S17. HSQC spectra (^{1}H 500 MHz; d_6-DMSO) of bacillimidazole E (**2**)....................pg. 12
Figure S18. COSY spectra (^{1}H 500 MHz; d_6-DMSO) of bacillimidazole E (**2**)....................pg. 13
Figure S19. ^{1}H,^{13}C-HMBC spectra (^{1}H 500 MHz; d_6-DMSO) of bacillimidazole E (**2**)...pg. 13
Figure S20. ^{1}H,^{15}N-HMBC spectra (^{1}H 500 MHz; d_6-DMSO) of bacillimidazole E (**2**)...pg. 14
Figure S21. HR-LCMS (ESI q-ToF) fragmentation of bacillimidazole E (**2**) ....pg. 14
Figure S22. LCMS analysis of the non-enzymatic reaction of phenethylamine with 2,3-butanedione in PBS to form bacillimidazole A (**1**) ....pg. 15
Figure S23. ^{1}H NMR spectra of 2,3-butanedione (600 MHz; $CDCl_3$)....................pg. 15
Figure S24. ^{1}H NMR spectra of freshly distilled 2,3-butanedione (600 MHz; $CDCl_3$).....pg. 16
Figure S25. ^{1}H NMR spectra from a kinetics experiment on the non-enzymatic reaction of phenethylamine with 2,3-butanedione in D_2O. ....pg. 17
Figure S26. Intensified ^{1}H NMR spectra from a kinetics experiment on the non-enzymatic reaction of phenethylamine with 2,3-butanedione in D_2O ....pg. 18
Figure S27. ^{1}H NMR spectra from individual components of the kinetics experiment on the non-enzymatic reaction of phenethylamine with 2,3-butanedione in D_2O ....pg. 19
Figure S28. LCMS chromatogram of the non-enzymatic reaction of phenethylamine with 2,3-butanedione conducted under an inert atmosphere. ....pg. 19
Figure S29. LCMS chromatogram of the non-enzymatic reaction of phenethylamine with 2,3-butanedione conducted in the absence of light....................pg. 20
Figure S30. Products and observed fragments for each of the non-enzymatic reaction ....pg. 22

Figure S31. Mass spectroscopy of phenethylamine non-enzymatic reactions to generate bacillimidazole A (**1**) (*m/z* 305), bacillimidazole B (**1a**) (*m/z* 319), and discolin A (**1b**) (*m/z* 347)....pg. 23
Figure S32. Mass spectroscopy of phethethylamine and tryptamine non-enzymatic reactions to generate bacillimidazole E (**2**) (*m/z* 344), bacillimidazole F (**2a)** (*m/z* 358), and discolin B (**2b**) (*m/z* 386)....pg. 24
Figure S33. Mass spectroscopy of tryptamine non-enzymatic reactions to generate bacillimidazole C (**3**) (*m/z* 383), bacillimidazole D (**3a**) (*m/z* 397), and discolin D/bacillimidazole G (**3b**) (*m/z* 425). ....pg. 25
Figure S34. Mass spectroscopy of tyramine non-enzymatic reactions to (**4**) (*m/z* 337), (**4a**) (*m/z* 351), and (**4b**) (*m/z* 397)....pg. 27
Figure S35. Mass spectroscopy of benzylamine non-enzymatic reactions to generate lepidiline A (**5**) (*m/z* 277), lepidiline B (**5a**) (*m/z* 291), and (**5b**) (*m/z* 319)....pg. 29
Figure S36. Mass spectroscopy of isobutyl amine non-enzymatic reactions to generate (**6**) (*m/z* 209), (**6a**) (*m/z* 223), and (**6b**) (*m/z* 251)....pg. 31
Figure S37. Mass spectroscopy of phenethylamine and tyramine non-enzymatic reactions to generate hydroxybacillimidazole A (**7**) (*m/z* 321), (**7a**) (*m/z* 335), and discolin E (**7b**) (*m/z* 363)....pg. 33
Figure S38. Mass spectroscopy of tryptamine and tyramine non-enzymatic reactions to generate (**8**) (*m/z* 360), (**8a**) (*m/z* 374), and discolin C (**8b**) (*m/z* 402) ....pg. 35
Figure S39. Mass spectroscopy of isobutyl amine and tryptamine non-enzymatic reactions to generate (**9**) (*m/z* 296), (**9a**) (*m/z* 310), and bacillimidazole J (**9b**) (*m/z* 338)....pg. 37
Figure S40. HR-LCMS analysis of egg collar 5C (site 1)....pg. 38
Figure S41. HR-LCMS analysis of egg collar 8C (site 1)....pg. 39
Figure S42. HR-LCMS analysis of egg collar 47C (site 2)....pg. 40
Figure S43. HR-LCMS analysis of egg collar 48C (site 2)....pg. 41
Figure S44. HR-LCMS analysis of egg collar 86C (site 3)....pg. 42
Figure S45. HR-LCMS analysis of egg collar 87C (site 3)....pg. 43
Figure S46. HR-LCMS analysis of egg collar 88C (site 3)....pg. 44
Figure S47. HR-LCMS analysis of egg collar 106C (site 4)....pg. 45
Figure S48. HR-LCMS analysis of egg collar 107C (site 4)....pg. 46
Figure S49. HR-LCMS analysis of egg collar 108C (site 4)....pg. 47
Figure S50. Growth inhibition of bacillimidazole A (**A**) (grey bars) and bacillimidazole E (**B**) (green bars) against the human pathogens *B. cereus, E. coli, S. aureus, M. smegmatis, L. monocytogenes, S. aureus* MRSA, and *B. subtilis* at 64 µg/mL. ....pg. 48
Figure S51. Genomic phylogram of C. *omnivescoria* EM610 generated by the Type Genome Server. "EM610(2)" is the query genome sequence ....pg. 49

Supplemental Tables....pg. 50
Table S1. Summary of all intruder assays ....pg. 50
Table S2. Genes involved in acetolactate pathway and acetolactate degradation....pg. 54
Table S3. Detection of bacillimidazole A (**1**) on Florida moon snail egg masses....pg. 55
Table S4. Antibacterial MIC values of bacillimidazoles A and E....pg. 55
Table S5. Tools used by the SeqCenter for genome assembly and annotation ....pg. 56

Supplementary Figures

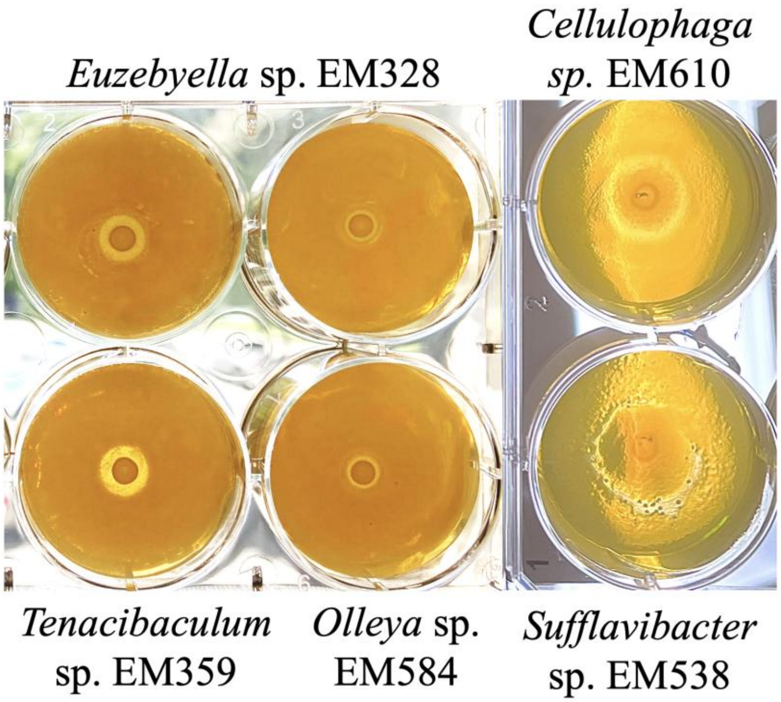

Figure S1. Intruder assays between *Flavobacteriaceae* that exhibited growth inhibition. Resident strains are indicated on the top of the wells, and intruder strains are shown on the bottom.

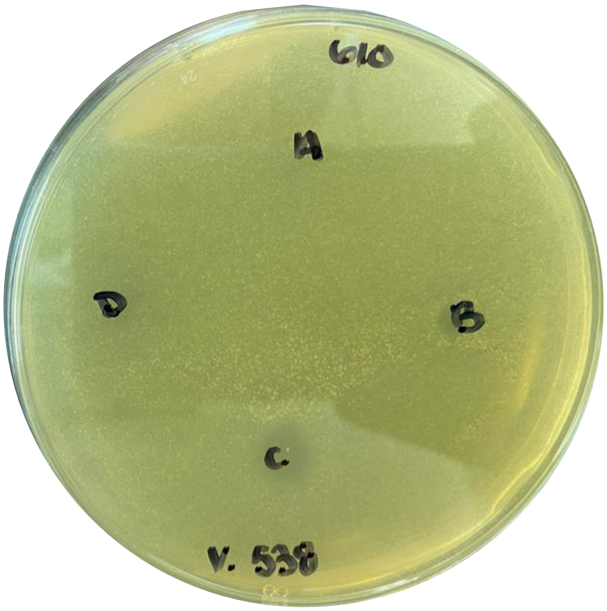

Figure S2. Spot-on-lawn assay of *C. omniverscoria* EM610 SPE chromatography fractions against environmental isolate *Sufflavibacter* sp. EM538. Small node visible around fraction B, larger node visible around fraction C. Fractions are designated as follows: 100% water = A, 50%/50% water/MeOH = B, 100% MeOH = C, solvent control = D.

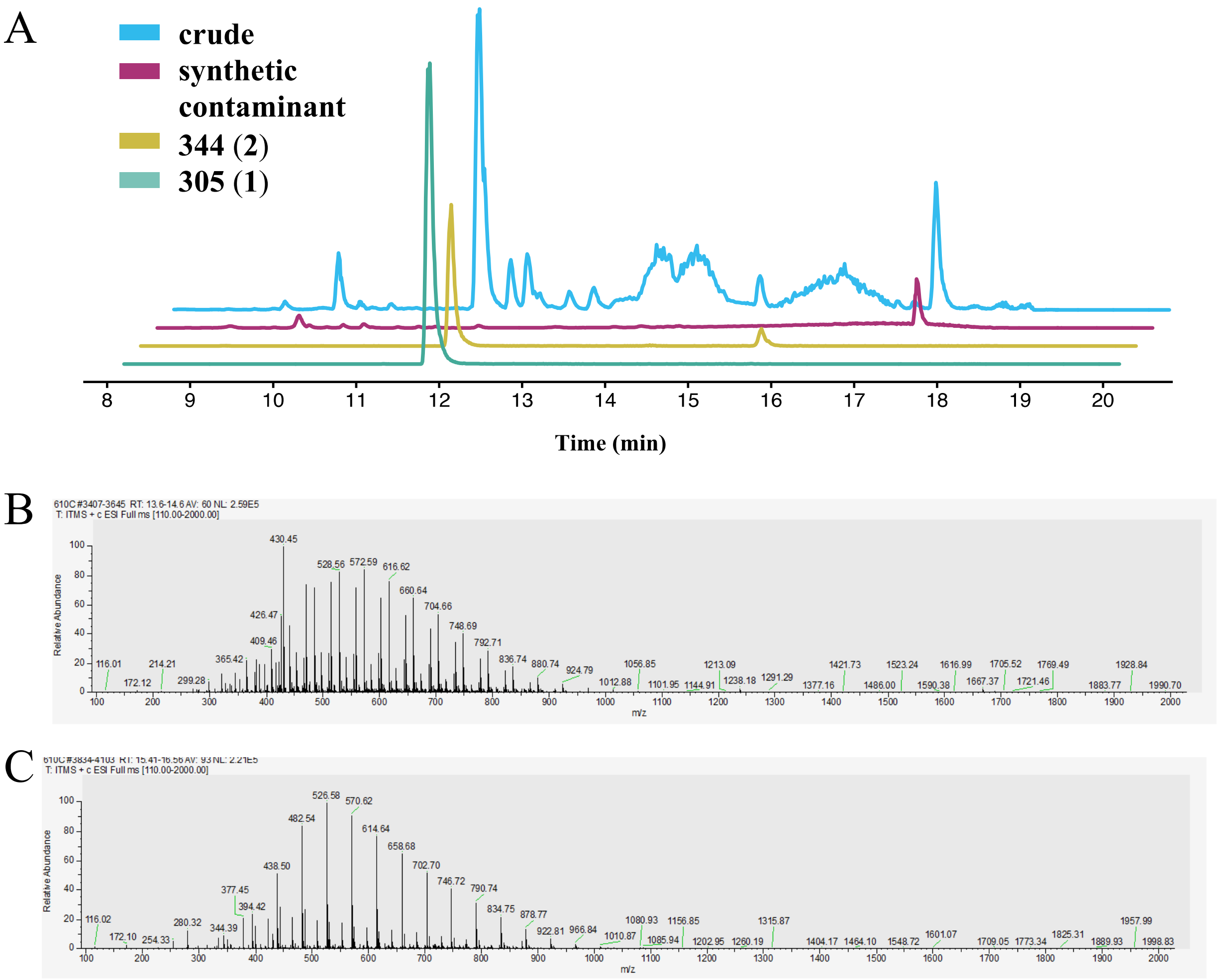

Figure S3. (**A**) LCMS chromatogram of EM610C (BPC - blue), where bacillimidazoles A (**1**) (EIC - green) and E (**2**) (EIC - yellow) are the major metabolites at 12 min. The peak at 17.2 min is column bleed from a synthetic compound not associated with this work (BPC – purple). Average ions in spectra between (**B**) 13.6–14.6 and (**C**) 15.1–16.5 min, which reveal ions characteristic of plastics.

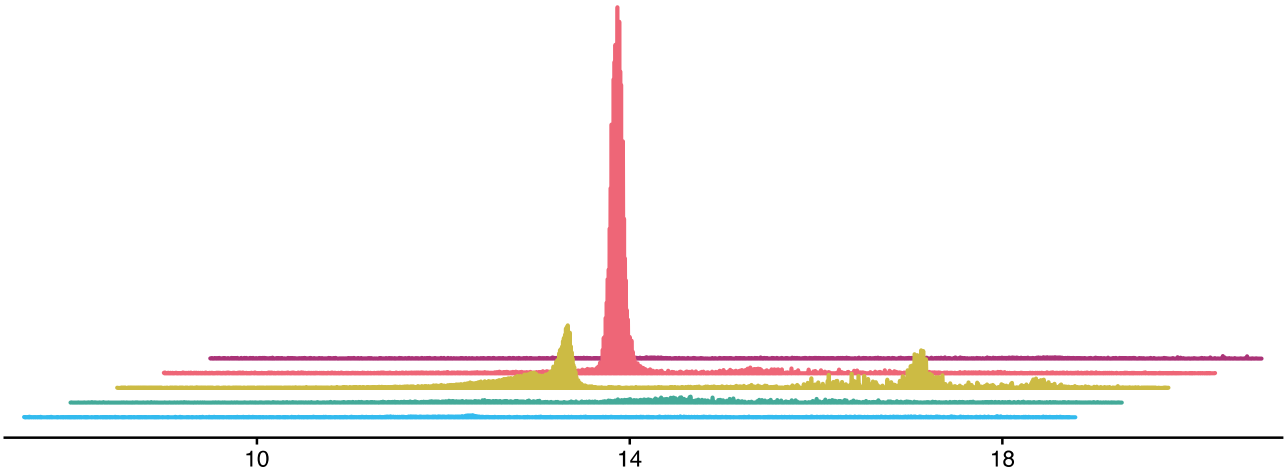

Figure S4. Extracted ion chromatogram of flash chromatography fractions showing bacillimidazoles A (**1**) and E (**2**) are majorly present in fraction D. Fraction A (blue) = 100% water, fraction B (green) = 75% water/25% CH_3CN, fraction C (yellow) = 50% water/50% CH_3CN, fraction D (pink) = 25% water/75% CH_3CN, and fraction E (purple) = 100 % CH_3CN.

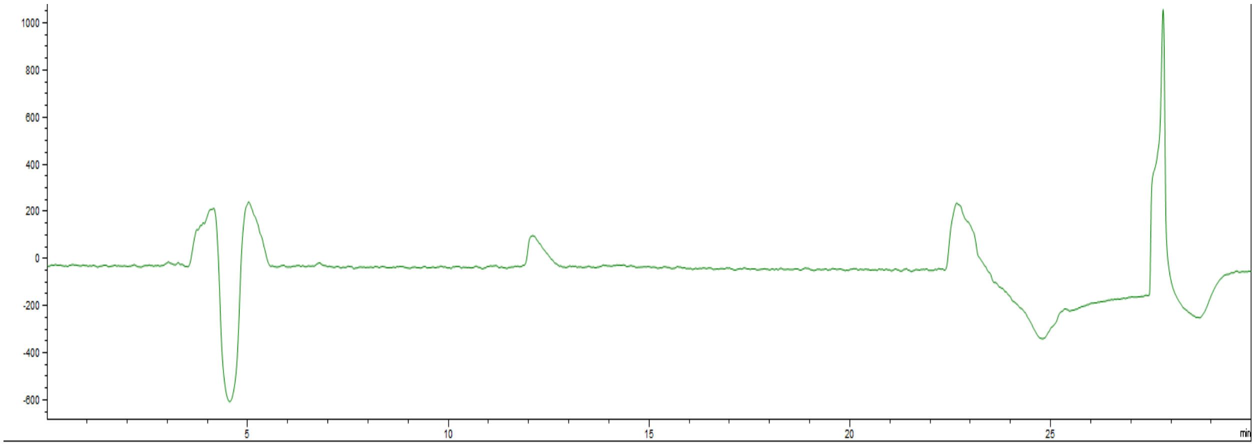

Figure S5. HPLC chromatogram of purified bacillimidazole A (**1**) at 210 nm (retention time = 12 min).

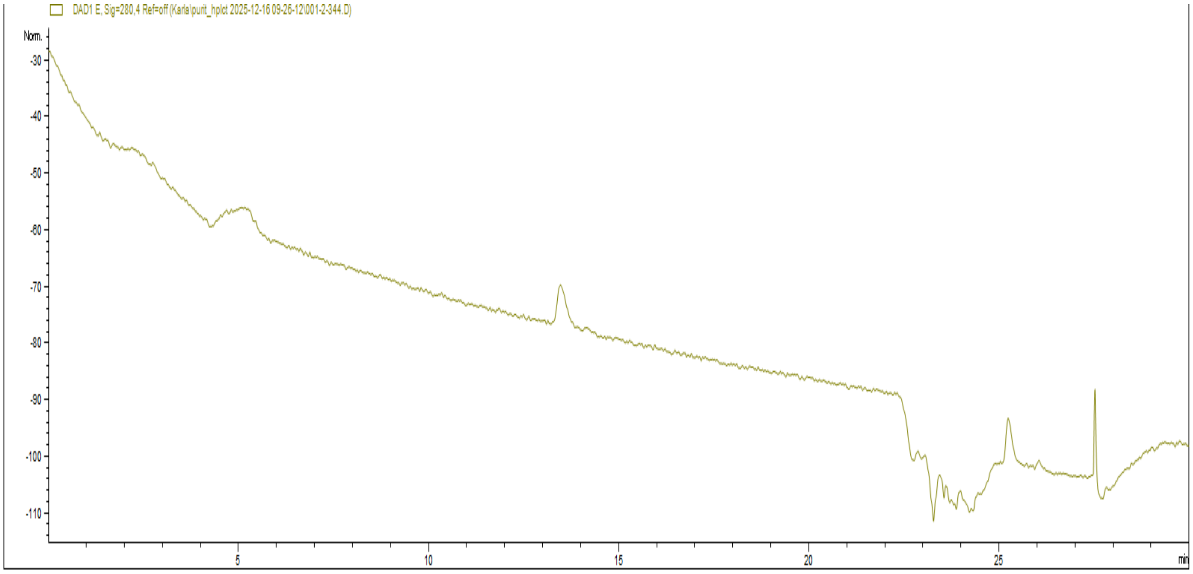

Figure S6. HPLC chromatogram of purified bacillimidazole E (**2**) at 280 nm (retention time = 13.5. min).

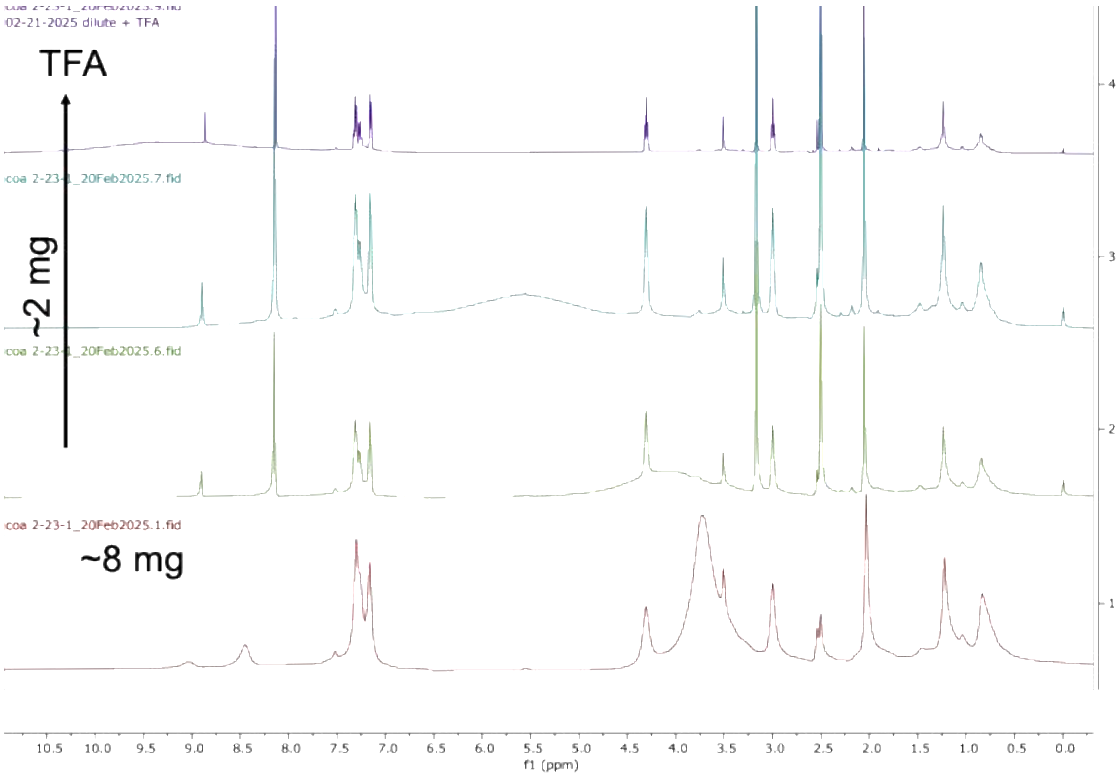

Figure S7. ^{1}H NMR spectrum (500 MHz; d_6-DMSO) of bacillimidazole A (**1**) at 8 mg with no TFA (bottom, spectrum 1), 2 mg with no TFA (spectrum 2), and 2 mg with increasing amounts of fuming TFA (spectra 3–4).

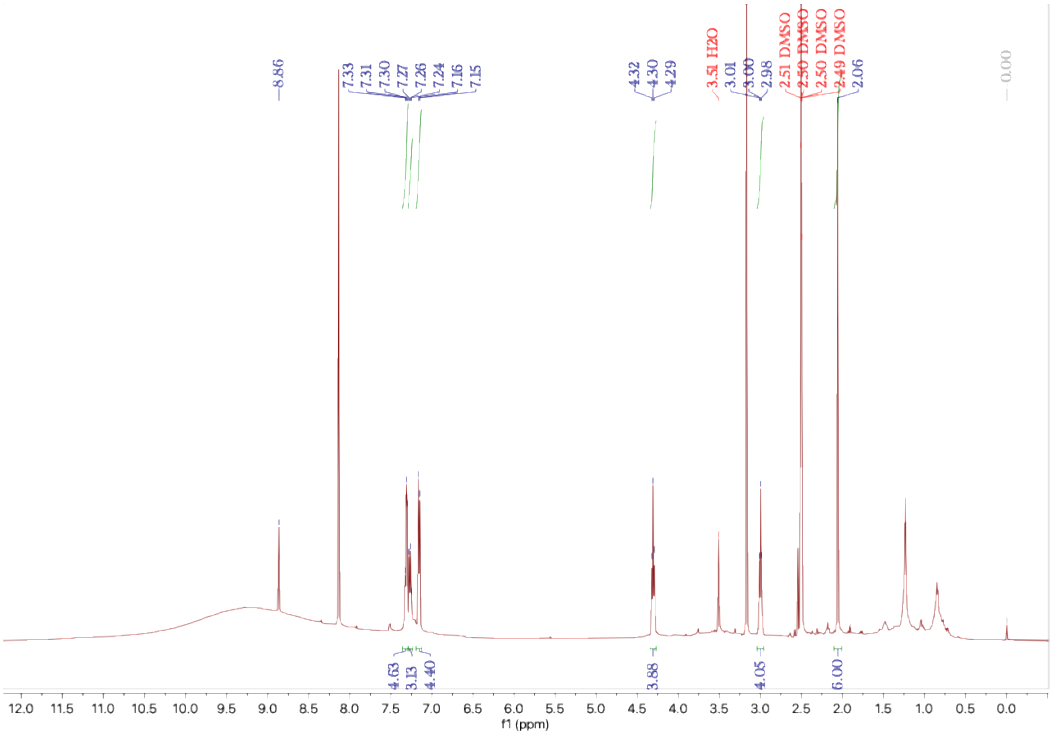

Figure S8. ^{1}H NMR spectra (500 MHz; d_6-DMSO) of bacillimidazole A (**1**).

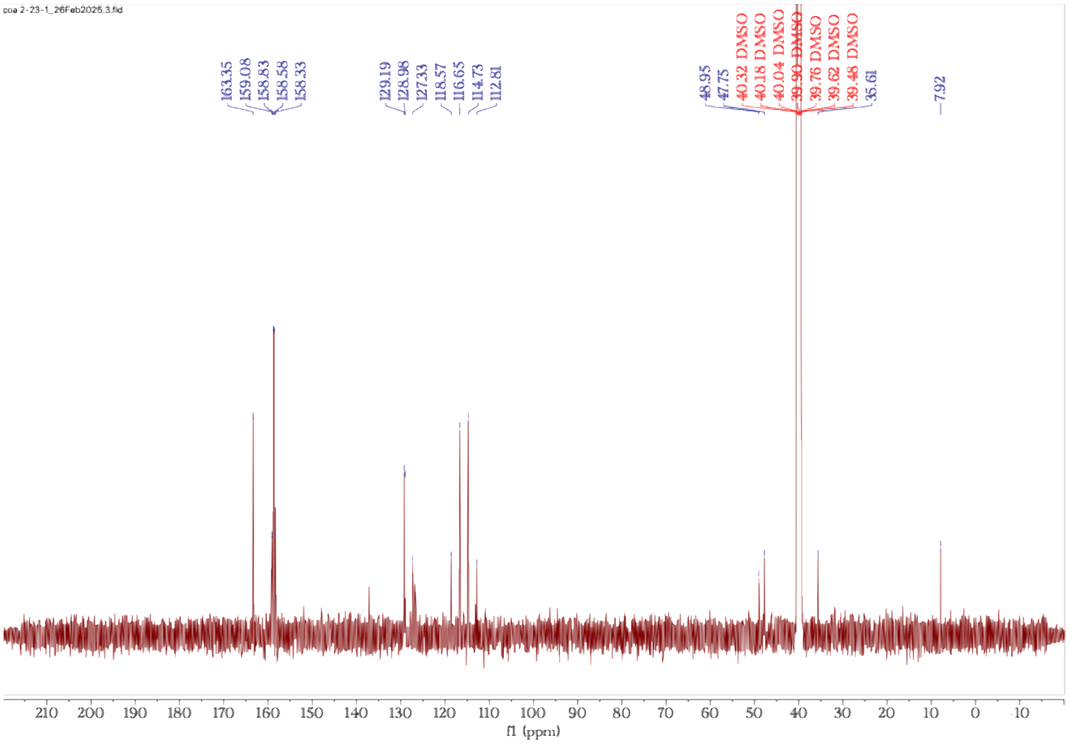

Figure S9. ^{13}C NMR spectra (125 MHz; d_6-DMSO) of bacillimidazole A (**1**).

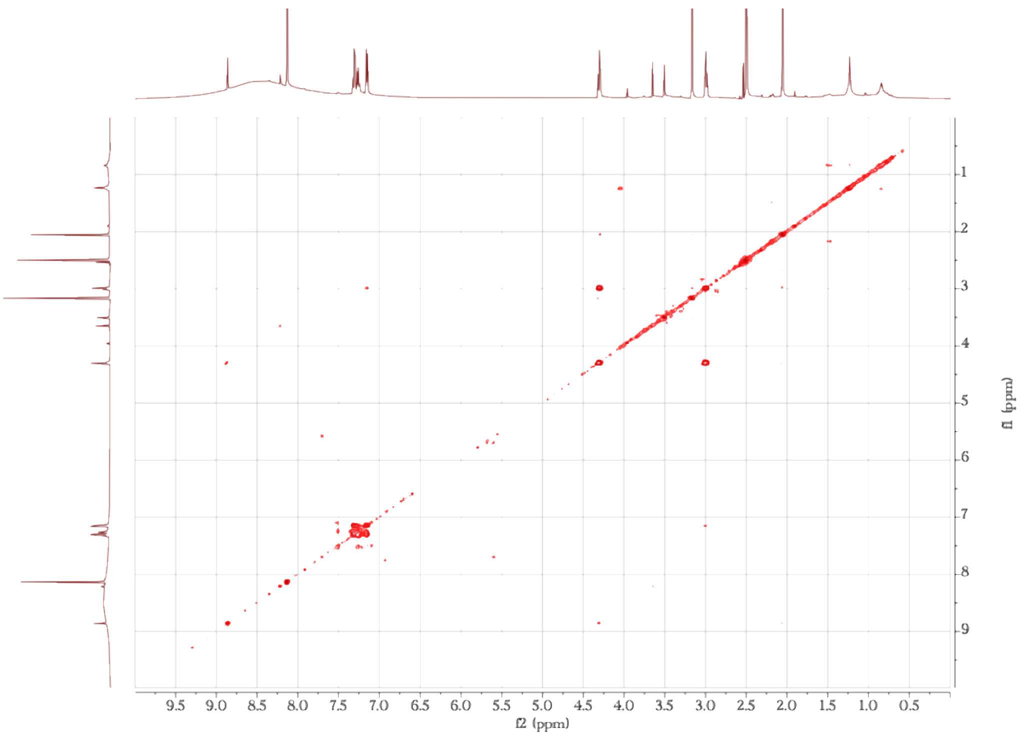

Figure S10. COSY spectra (^{1}H 500 MHz; d_6-DMSO) of bacillimidazole A (**1**).

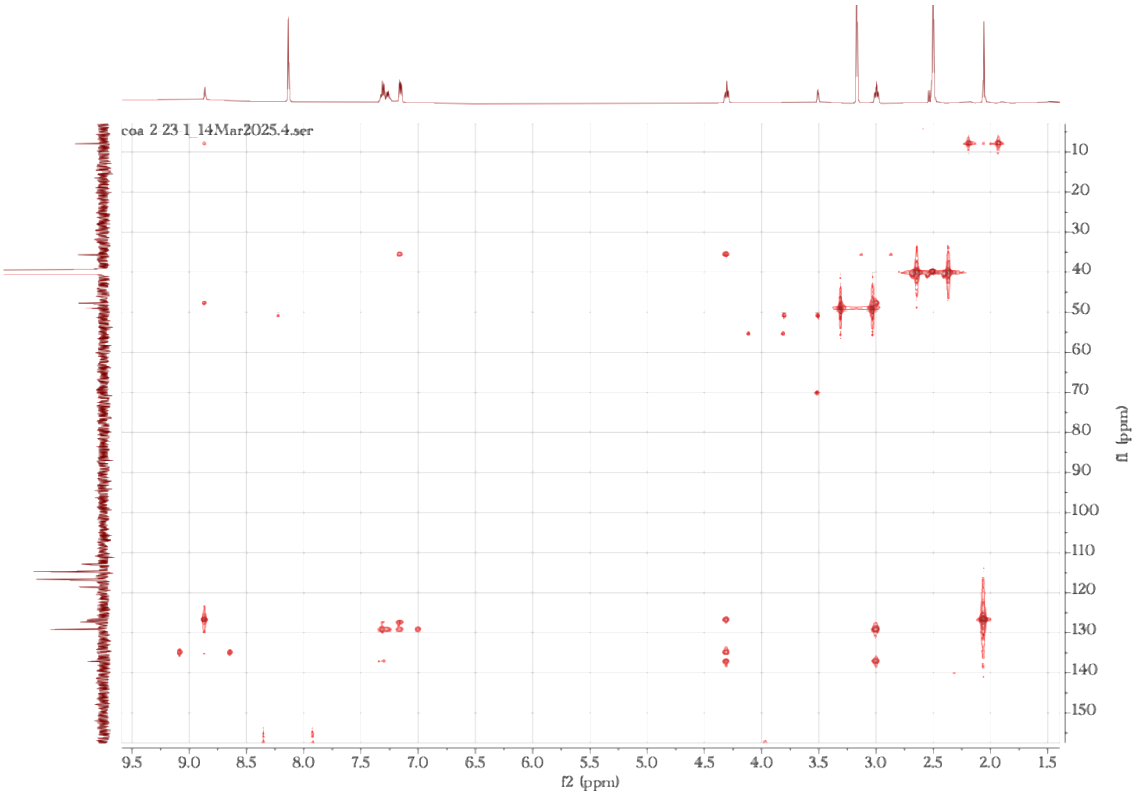

Figure S11. ^{1}H,^{13}C-HMBC spectra (^{1}H 500 MHz; d_6-DMSO) of bacillimidazole A (**1**).

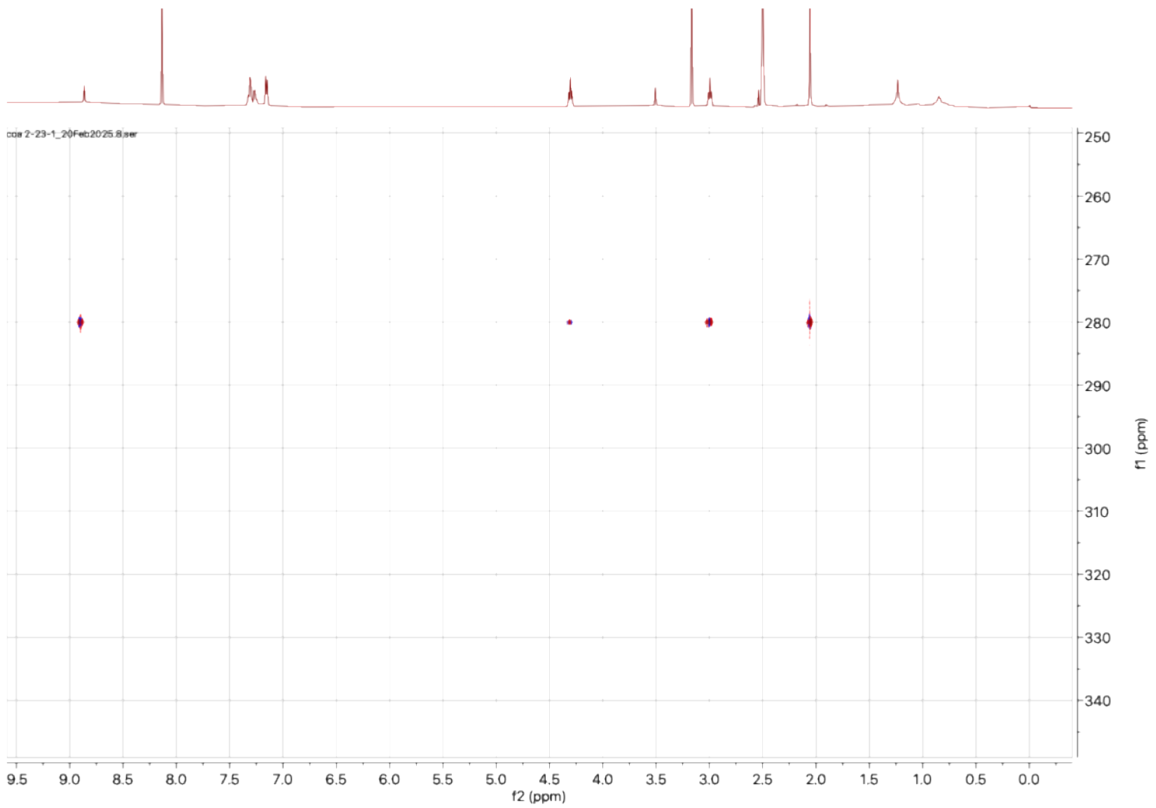

Figure S12. $^{1}H,^{15}N$-HMBC spectra (^{1}H 500 MHz; d_6-DMSO) of bacillimidazole A (**1**).

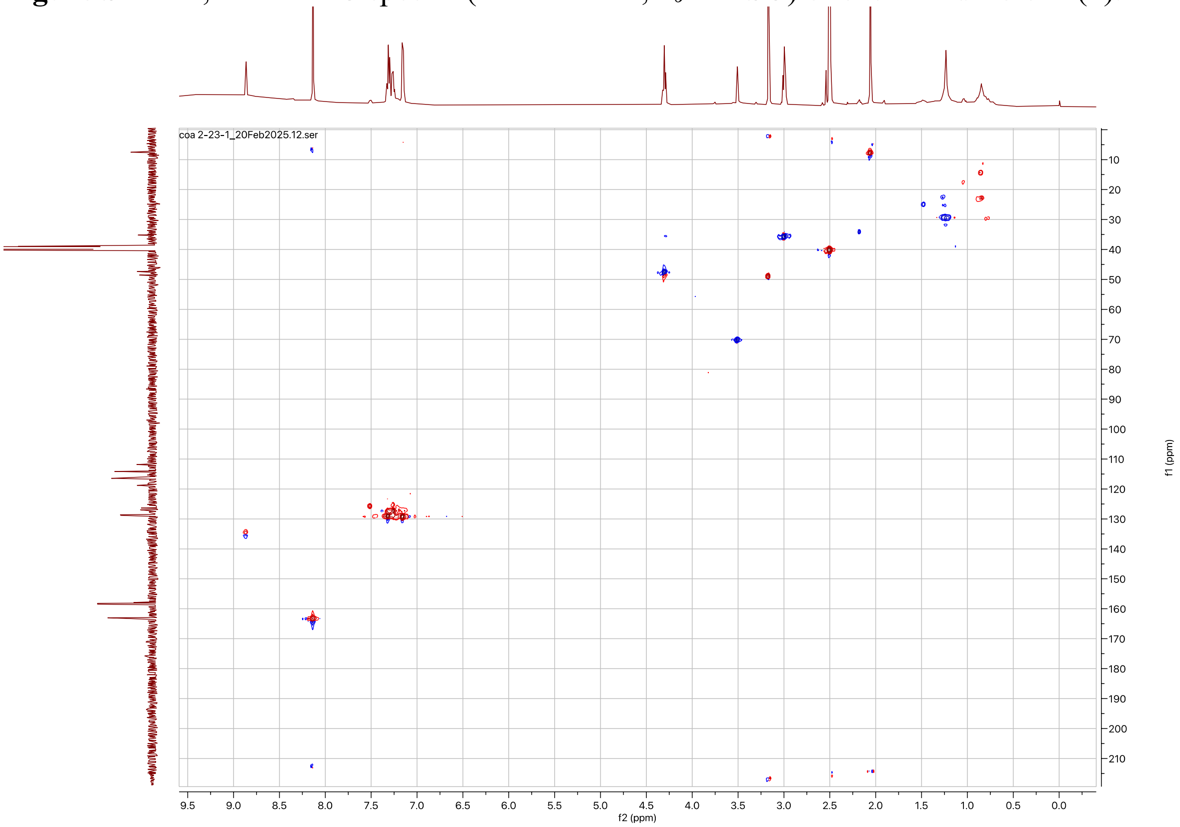

Figure S13. $^{1}H,^{13}C$-HMBC spectra (^{1}H 500 MHz; d_6-DMSO) of bacillimidazole A (**1**).

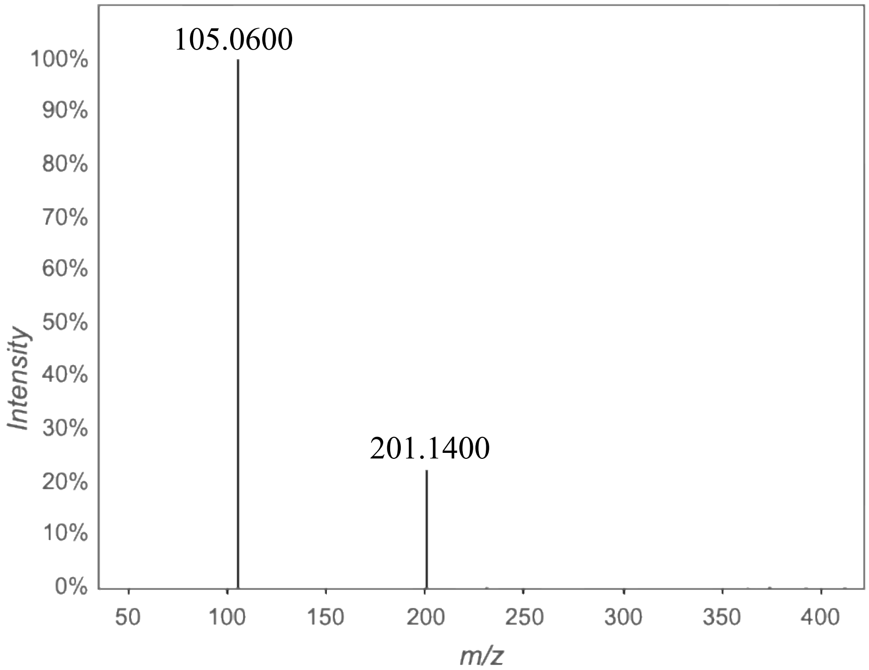

Figure S14. HR-LCMS (ESI q-ToF) fragmentation of bacillimidazole A (**1**) (USI: mzspec:MassIVE:TASK-8724e892b45f4db091944806b4d25cce-spectra/specs_ms.mgf:scan:20585).

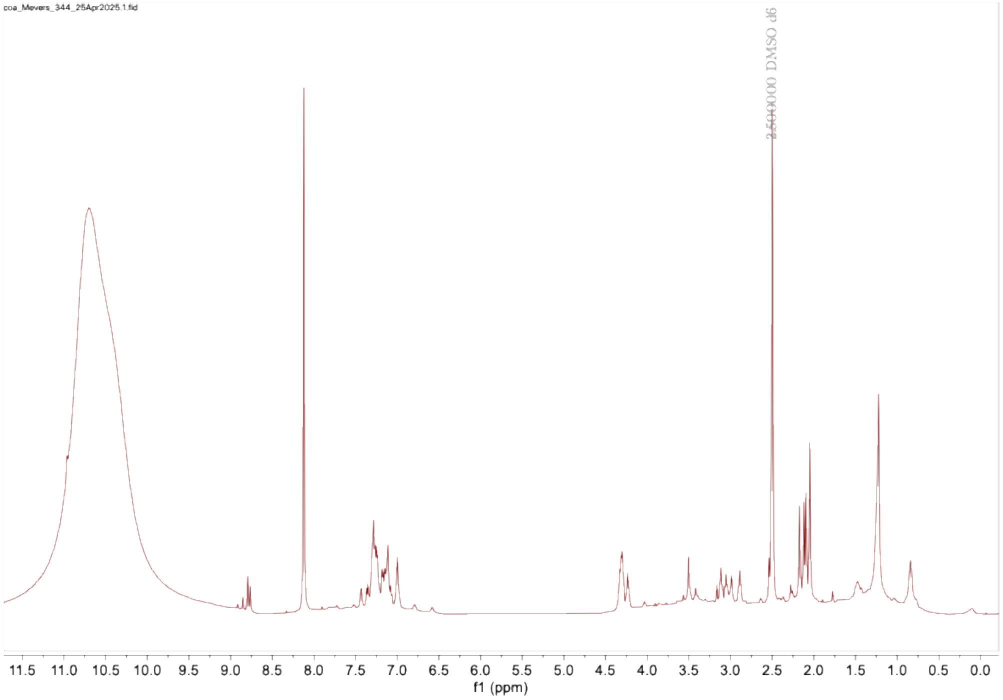

Figure S15. ^{1}H NMR spectra (500 MHz; d_6-DMSO) of bacillimidazole E (**2**). Based on LC (**Figure S6**) this sample looks to be 90% pure. Acidifying with TFA did not sharpen the peaks as significantly as for bacillimidazole A. There also appears to be peak duplications that may be arising from pi-stacking between the imidazolium ring and the side chains.

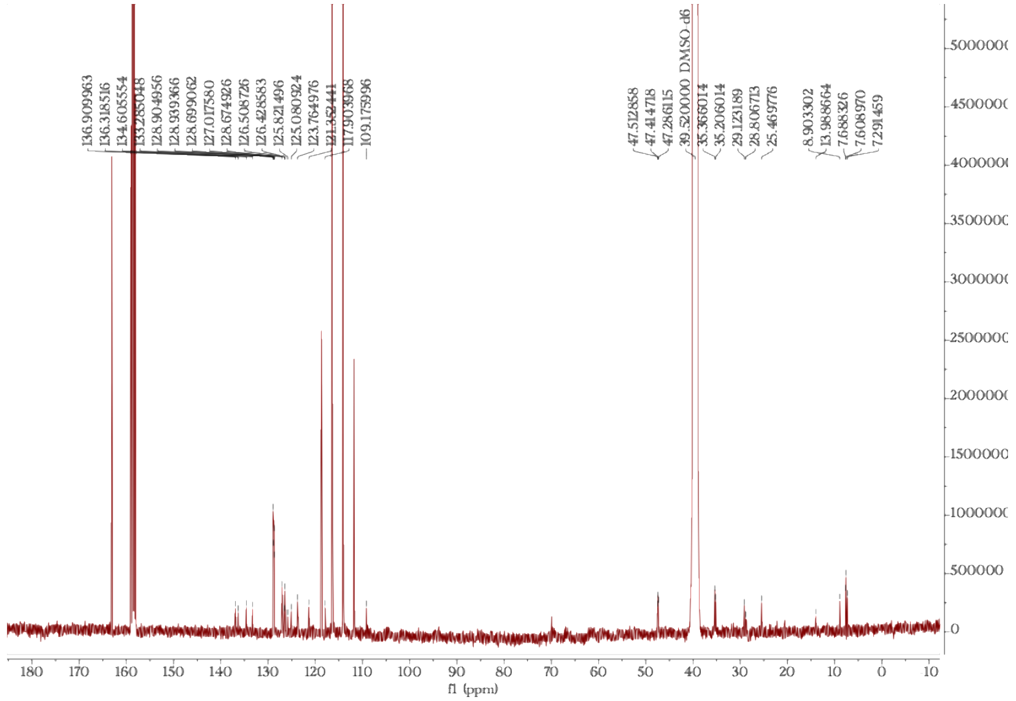

Figure S16. ^{13}C NMR spectra (125 MHz; d_6-DMSO) of bacillimidazole E (**2**). TFA was added to sharpen the peaks and accounts for many of the large signals.

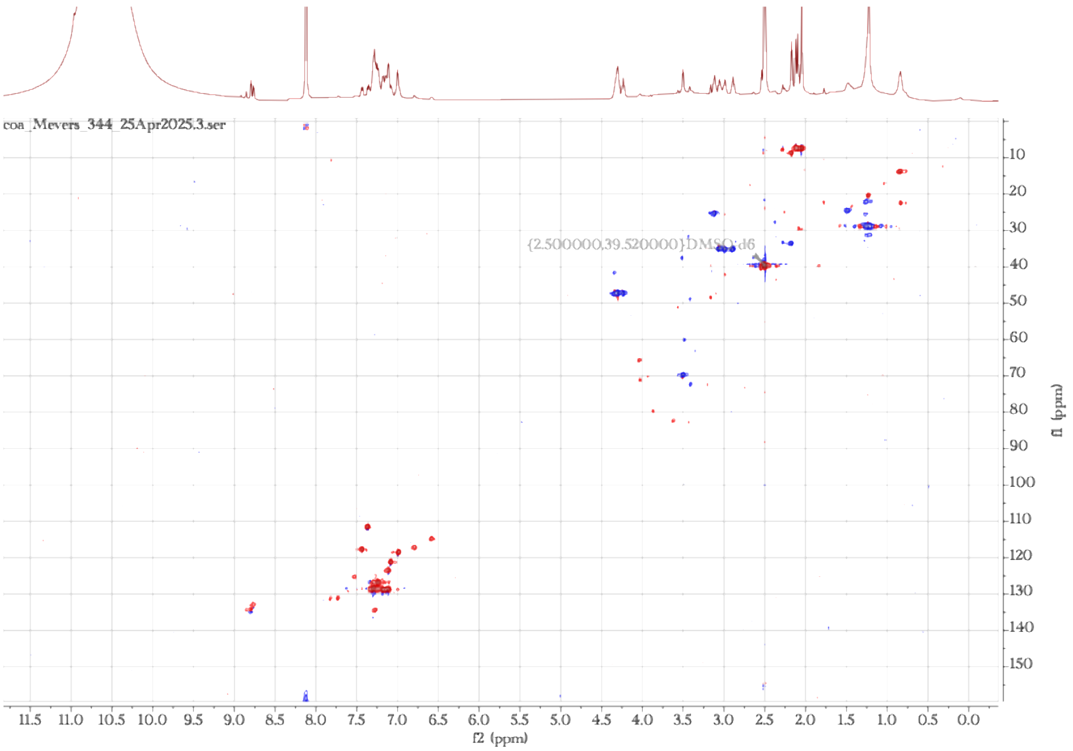

Figure S17. HSQC spectra (^1H 500 MHz; d_6-DMSO) of bacillimidazole E (**2**).

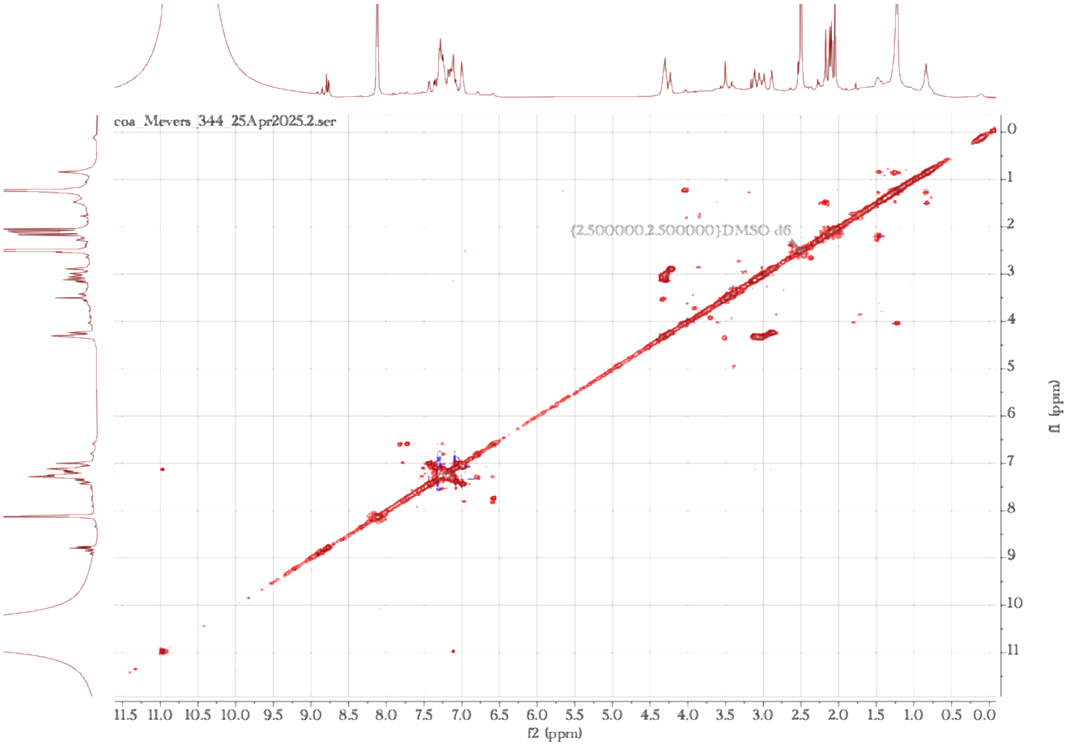

Figure S18. COSY spectra (^{1}H 500 MHz; *d*$_6$-DMSO) of bacillimidazole E (**2**).

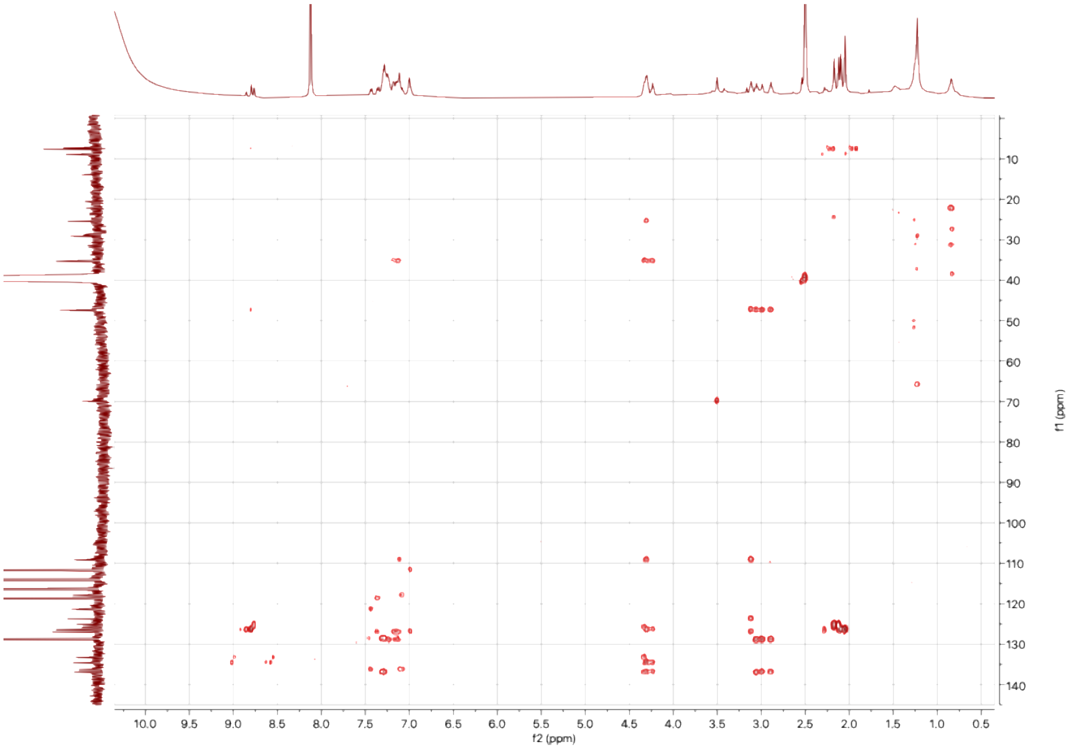

Figure S19. ^{1}H,^{13}C-HMBC spectra (^{1}H 500 MHz; *d*$_6$-DMSO) of bacillimidazole E (**2**).

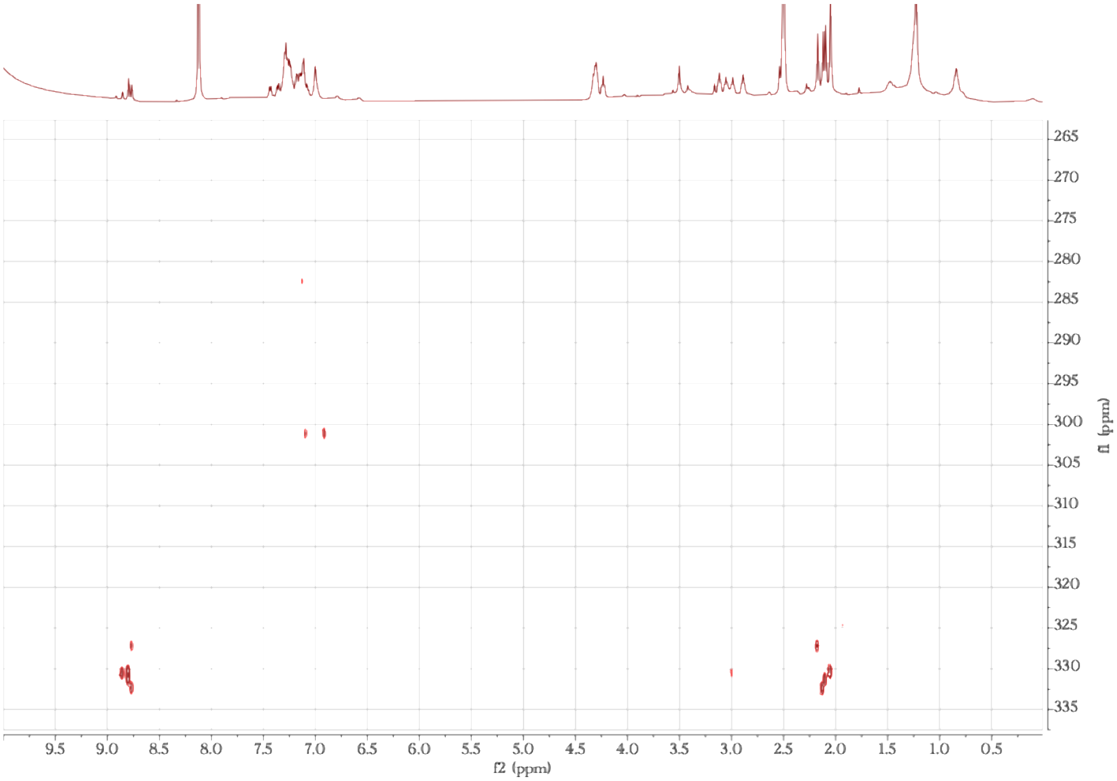

Figure S20. $^{1}H,^{15}N$-HMBC spectra (^{1}H 500 MHz; d_6-DMSO) of bacillimidazole E (**2**).

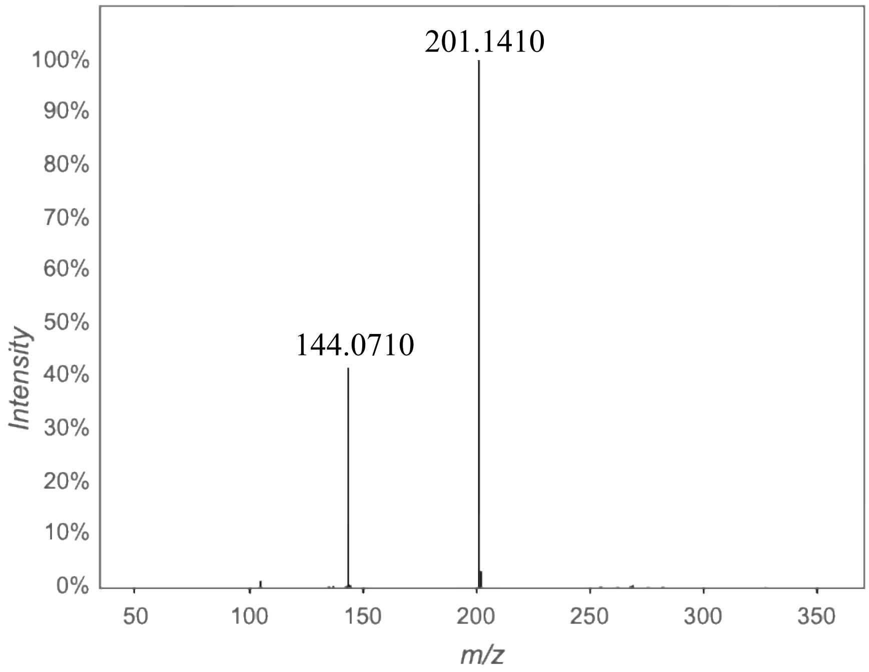

Figure S21. HR-LCMS (ESI q-ToF) fragmentation of bacillimidazole E (**2**). (USI: mzspec:MassIVE:TASK-8724e892b45f4db091944806b4d25cce-spectra/specs_ms.mgf:scan:30833)

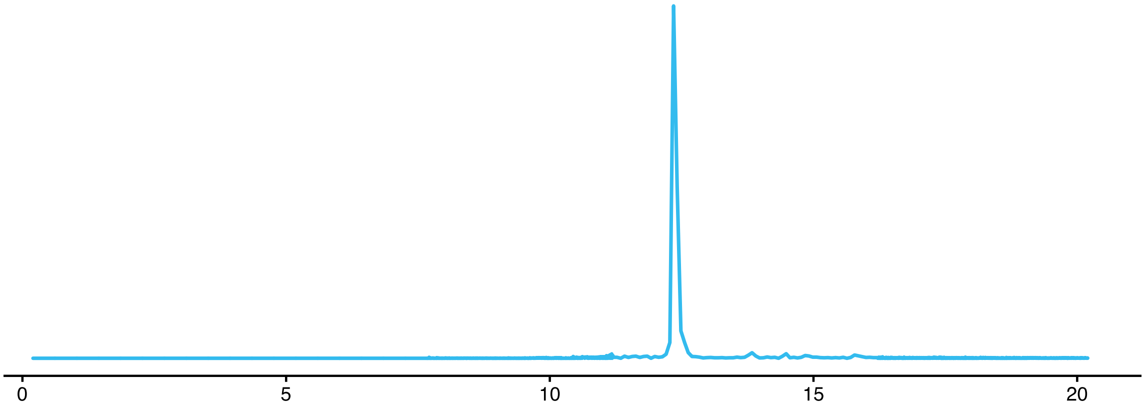

Figure S22. LCMS analysis of the non-enzymatic reaction of phenethylamine with 2,3-butanedione in PBS to form bacillimidazole A (**1**)

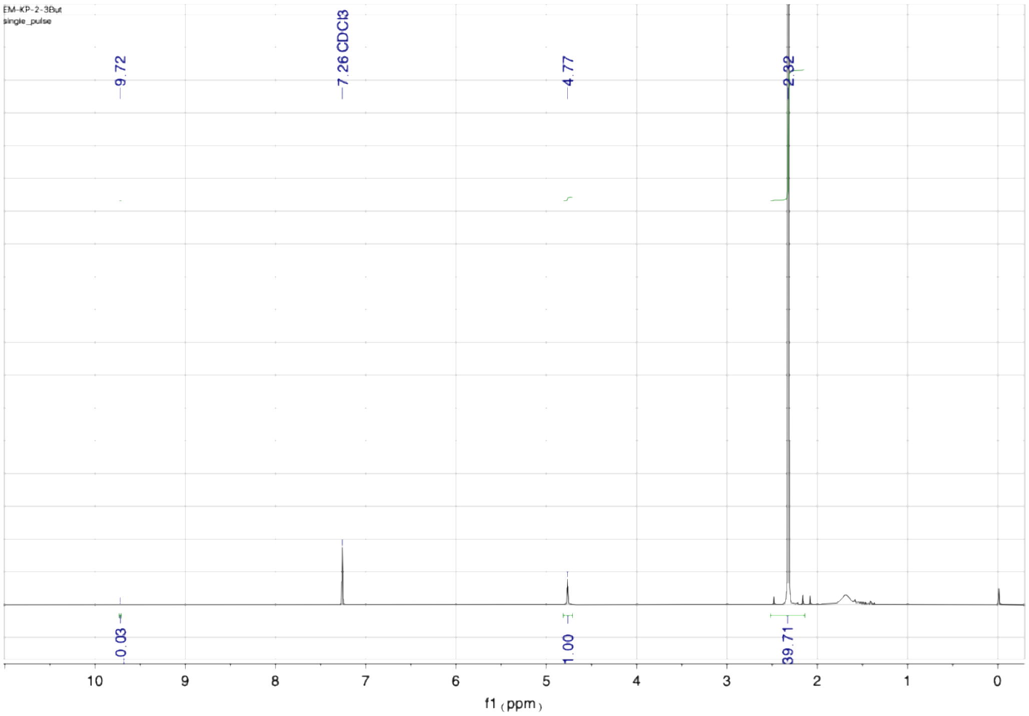

Figure S23. ^{1}H NMR spectra of 2,3-butanedione ($CDCl_3$; 600 MHz). Integration confirms a ratio of 2,3-butanedione, methylene glycol, and formaldehyde at a 40:1:0.03.

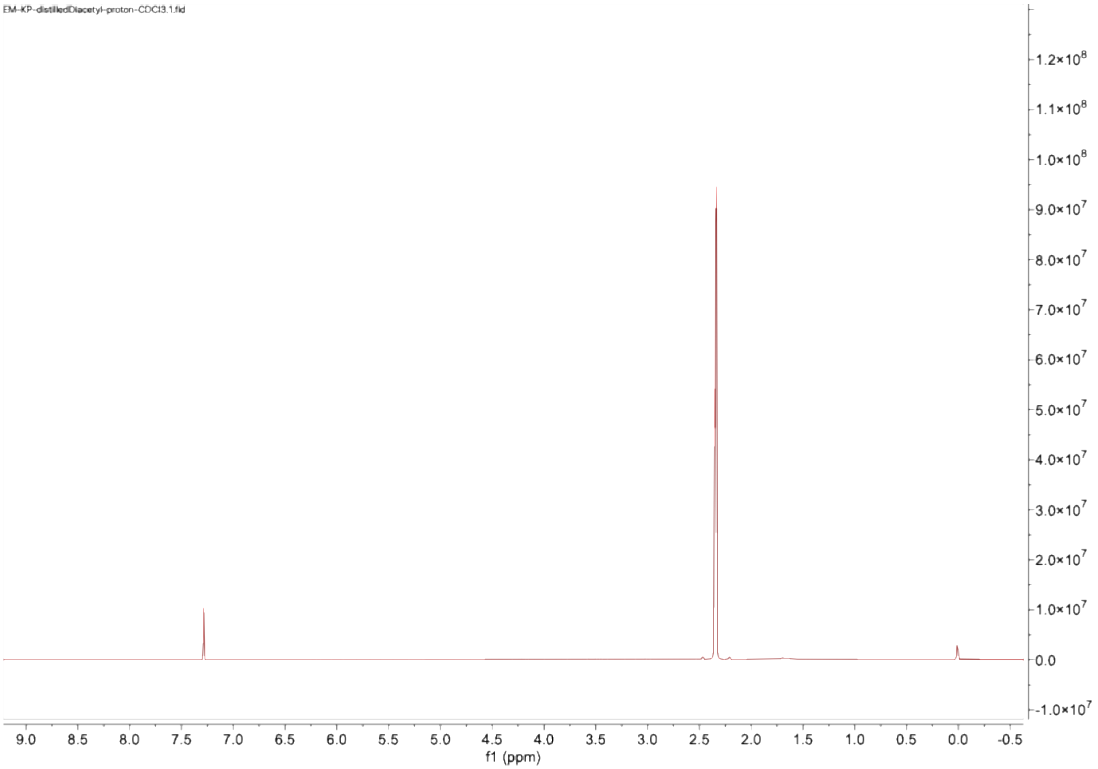

Figure S24. ^1H NMR spectra of freshly distilled 2,3-butanedione ($CDCl_3$; 500 MHz). Integration confirms a ratio of 2,3-butanedione and methylene glycol of 29,733:1 with no detection of formaldehyde.

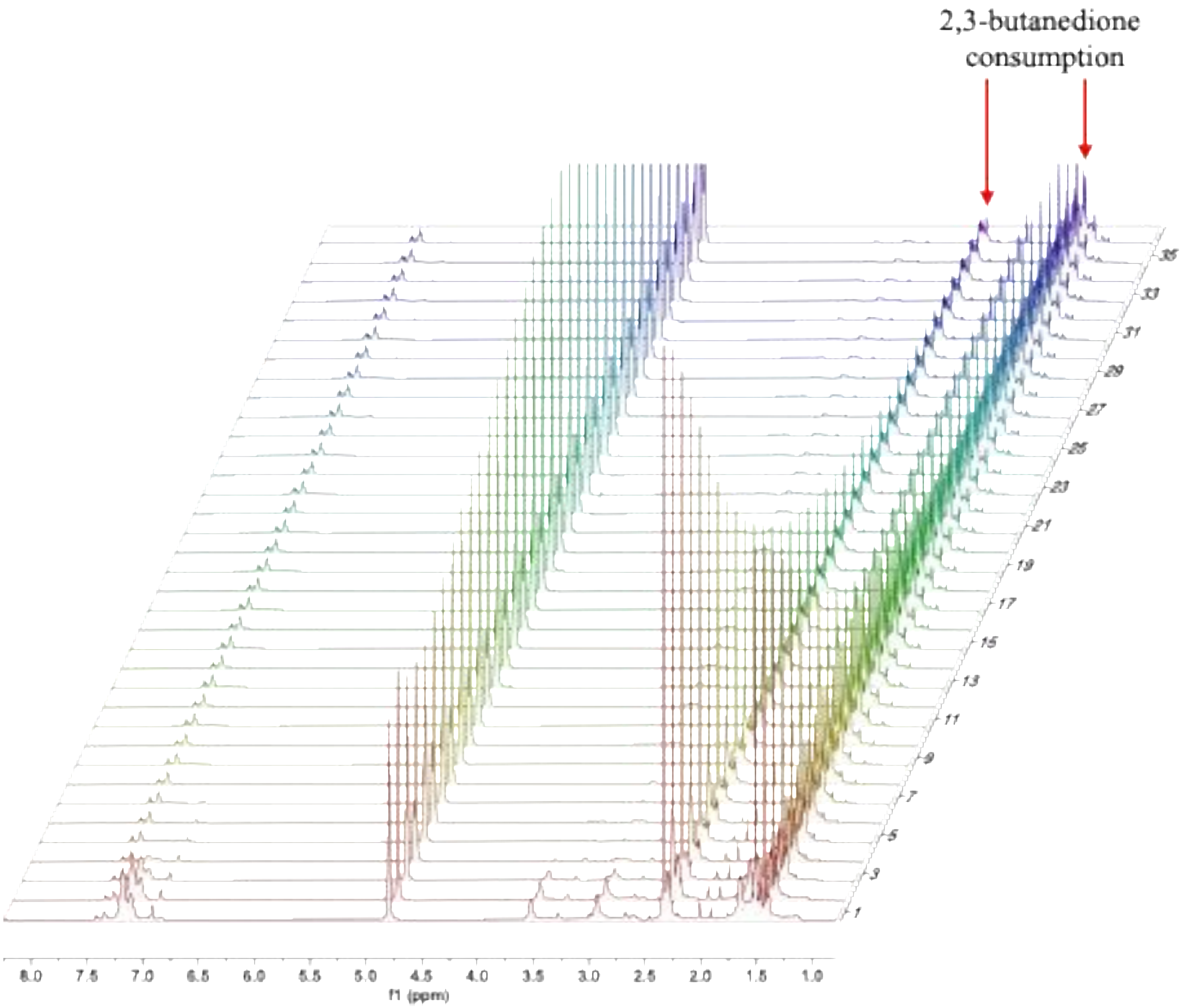

Figure S25. ^{1}H NMR spectra from a kinetics experiment on the non-enzymatic reaction of phenethylamine with 2,3-butanedione in D_2O. Y-axis numbers are independent spectra that were acquired every 5 min (8 scans and 5 sec relaxation delay). Over time, 2,3-butanedione is consumed as evidenced by a decrease in the relative peak height [sharp decline over the first hour (scans 1 – 10)].

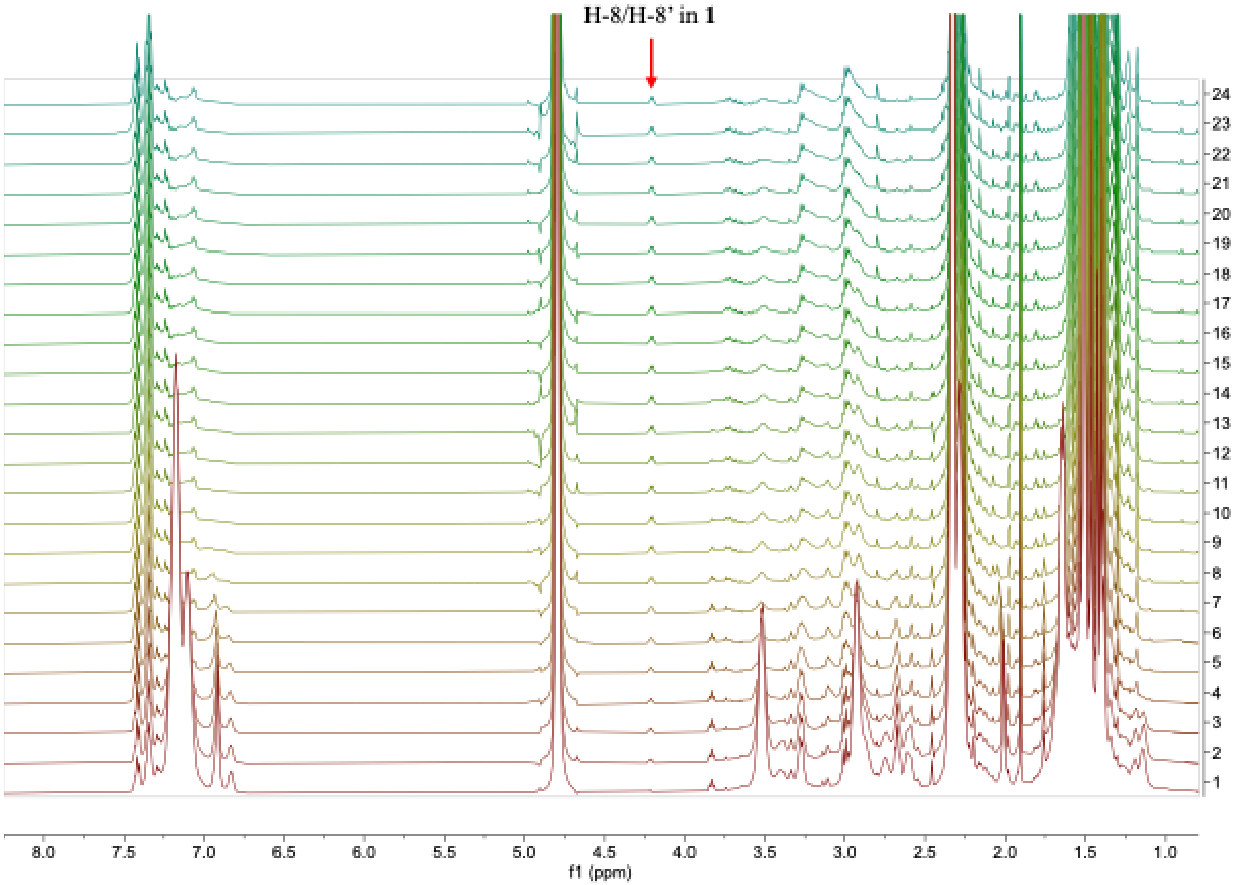

Figure S26. Intensified ^{1}H NMR spectra from a kinetics experiment on the non-enzymatic reaction of phenethylamine with 2,3-butanedione in D_2O. Y-axis numbers are independent spectra that were acquired every 5 min (8 scans and 5 sec relaxation delay). Within 5 min, **1** is beginning to be formed as evidenced by the appearance of the peak at 4.27 ppm, which is H-8/H-8'.

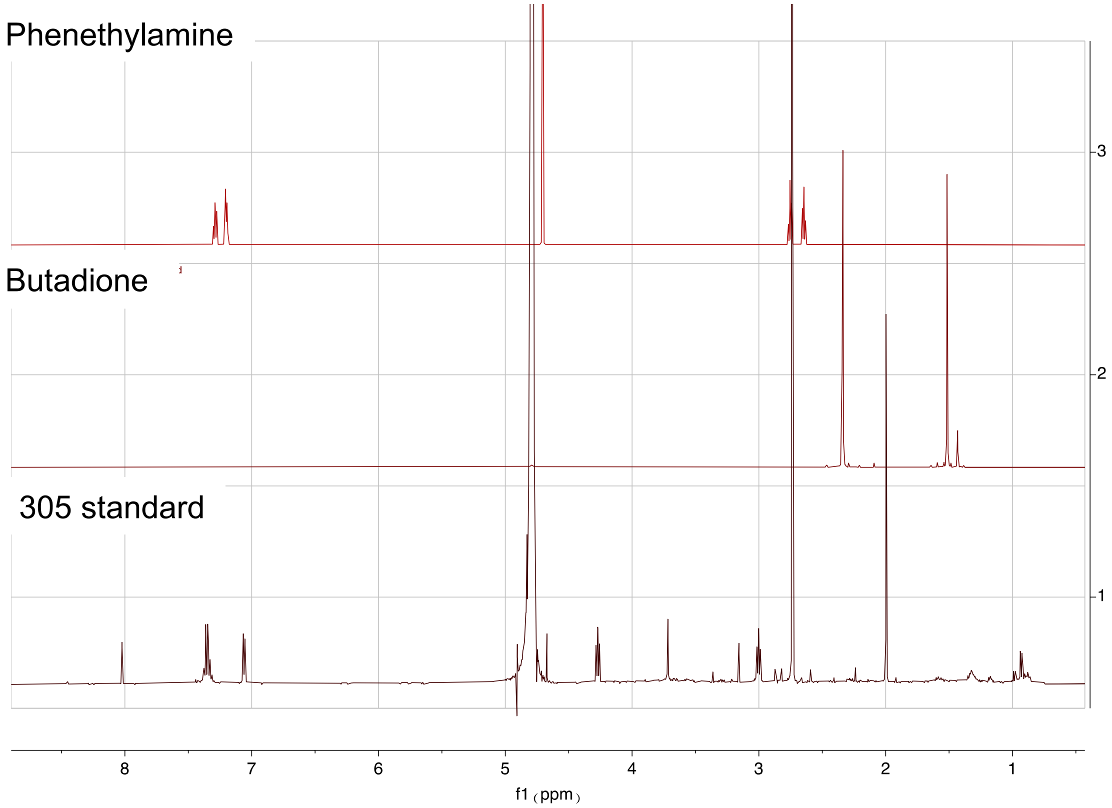

Figure S27. ^{1}H NMR spectra from individual components of the kinetics experiment on the non-enzymatic reaction of phenethylamine with 2,3-butanedione in D_2O.

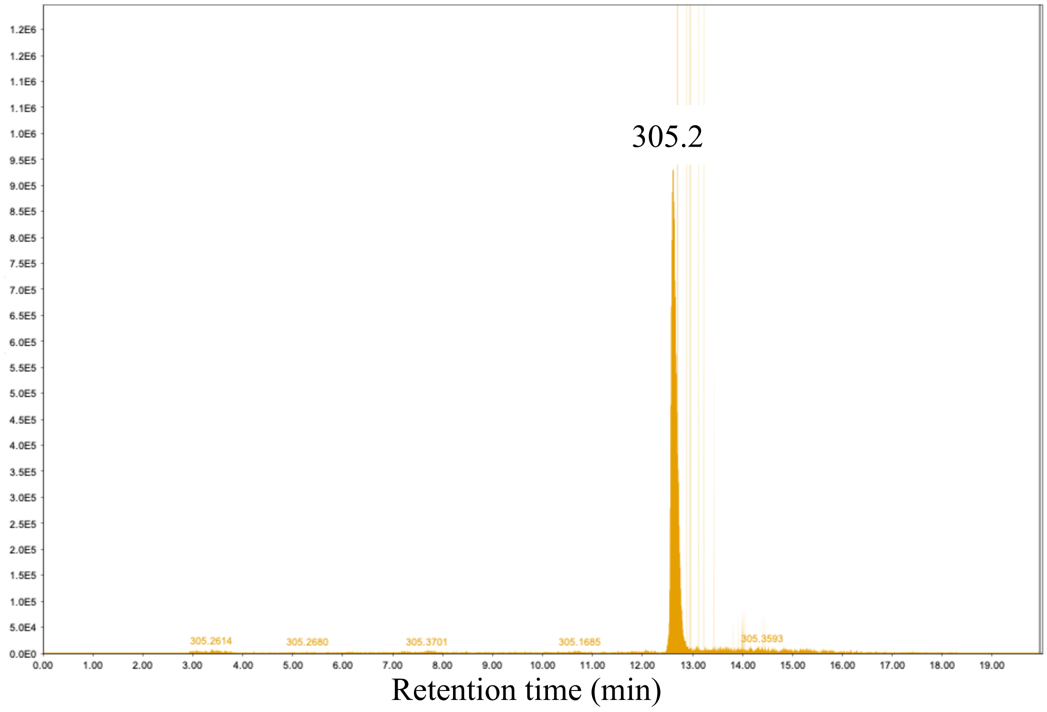

Figure S28. LCMS chromatogram of the non-enzymatic reaction of phenethylamine with 2,3-butanedione conducted under an inert atmosphere.

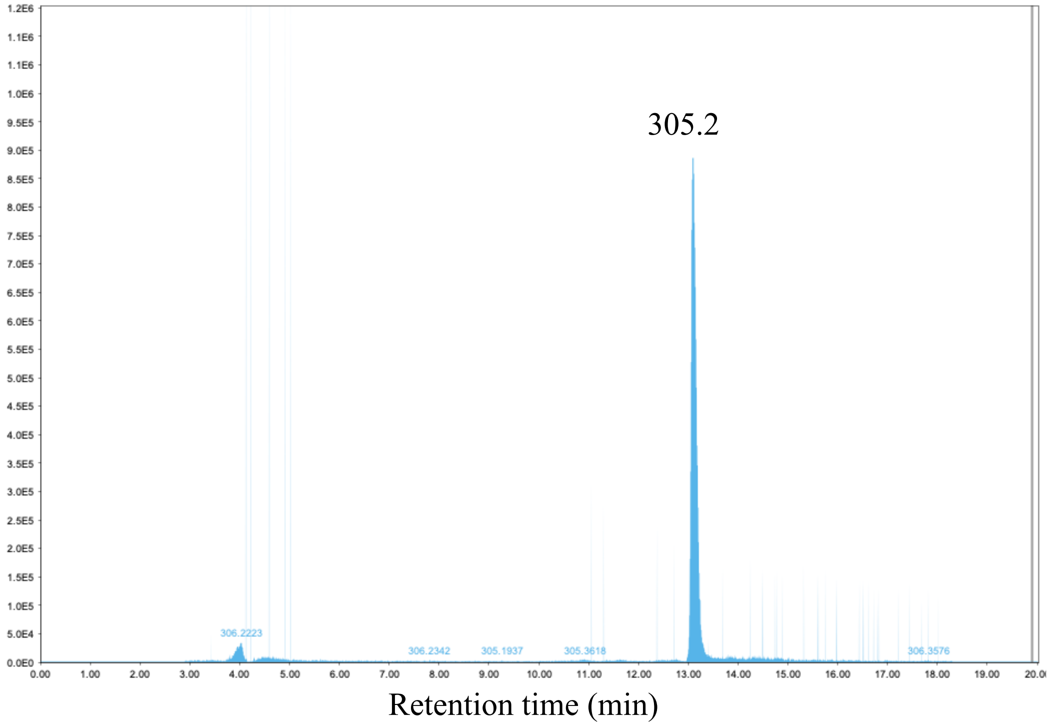

Figure S29. LCMS chromatogram of the non-enzymatic reaction of phenethylamine with 2,3-butanedione conducted in the absence of light.

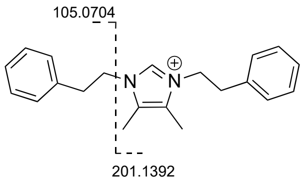

bacillimidazole A (**1**)

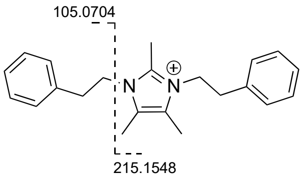

bacillimidazole B (**1a**)

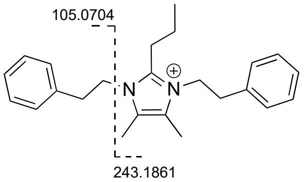

discolin A (**1b**)

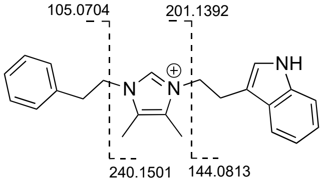

bacillimidazole E (**2**)

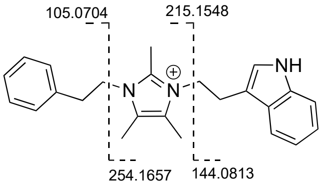

bacillimidazole F (**2a**)

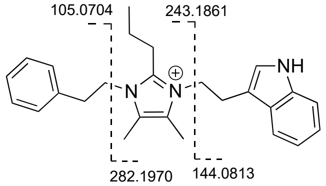

discolin B (**2b**)

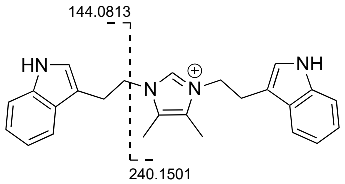

bacillimidazole C (**3**)

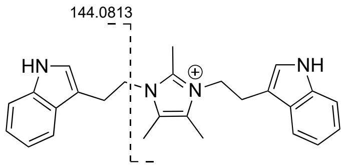

bacillimidazole D (**3a**)

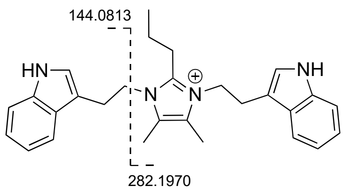

discolin D (**3b**)
bacilimidazole G

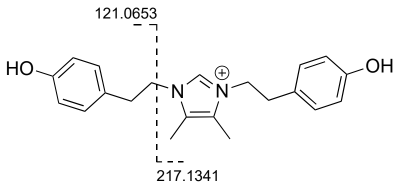

4

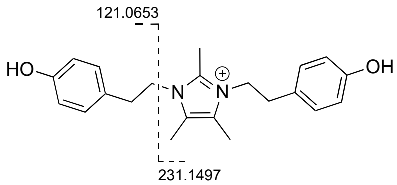

4a

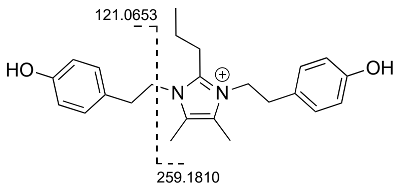

4b

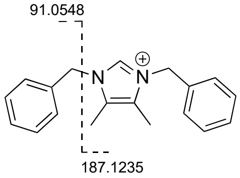

lepidiline A (**5**)

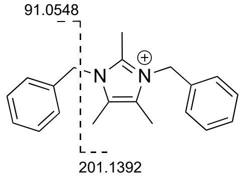

lepidiline B (**5a**)

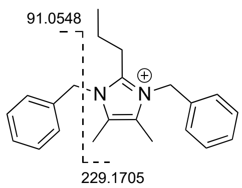

5b

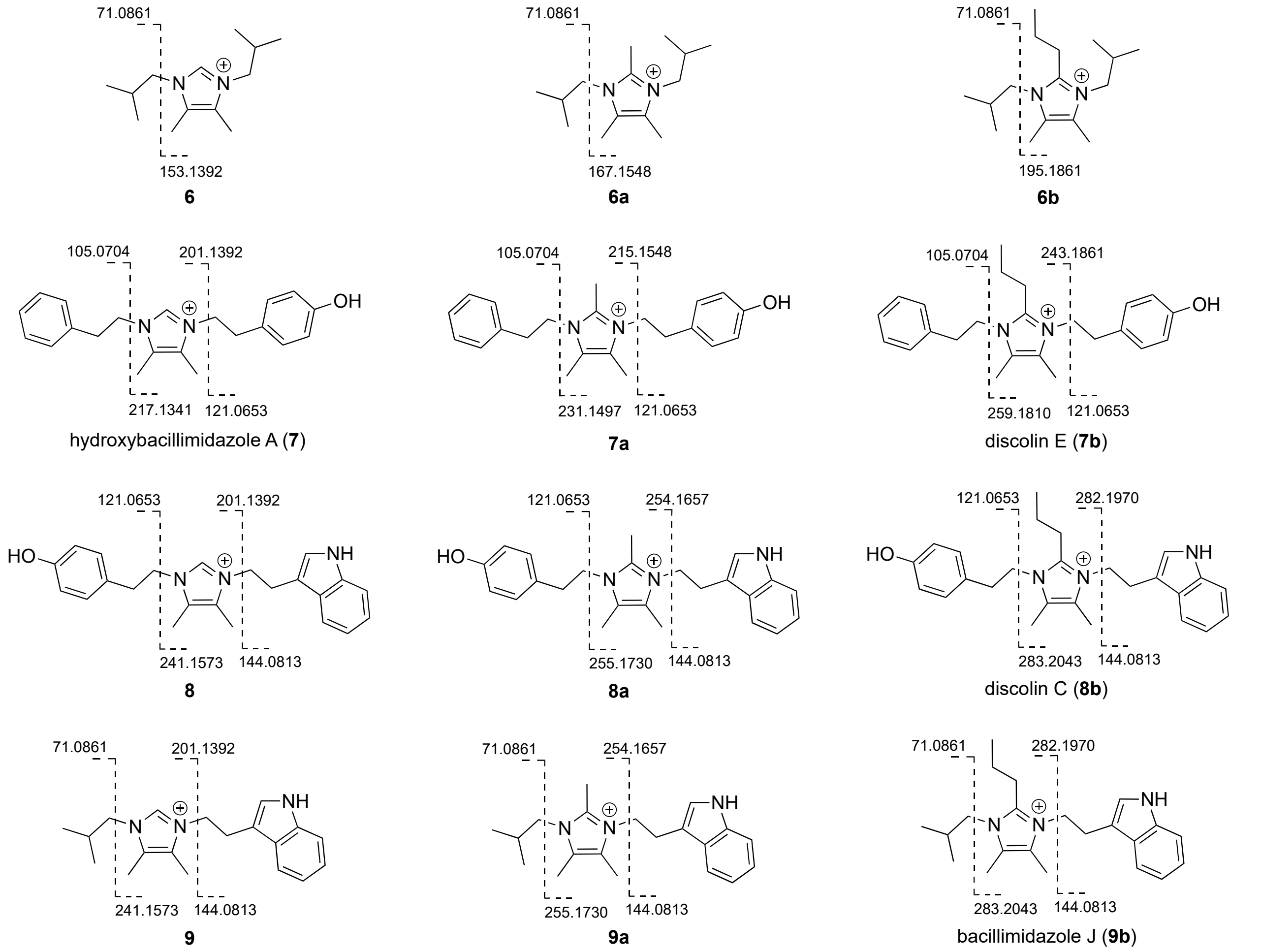

Figure S30. Products and observed fragments for each of the non-enzymatic reactions.

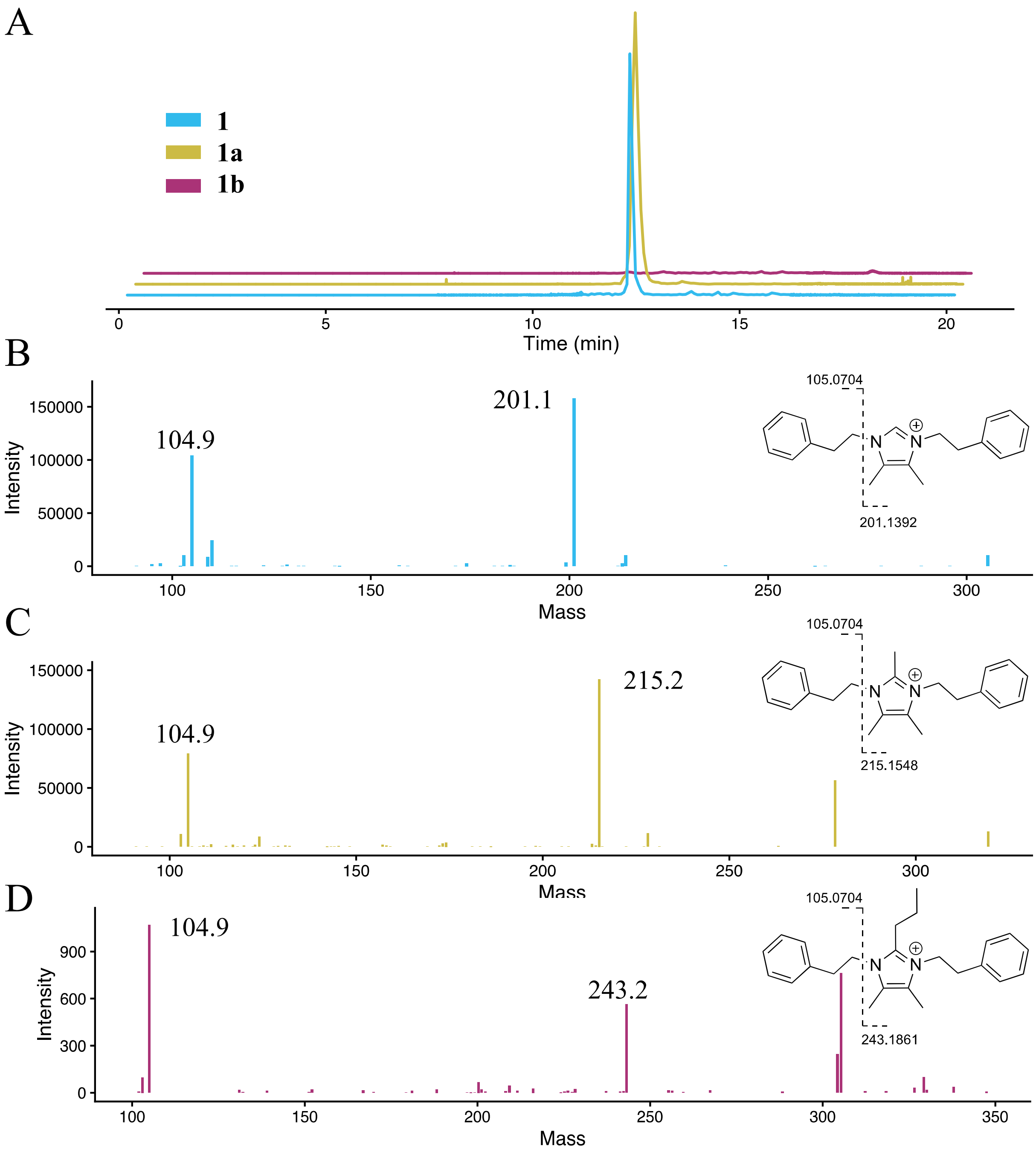

Figure S31. Mass spectroscopy of phethethylamine non-enzymatic reactions to generate bacillimidazole A (**1**) (305 *m/z*), bacillimidazole B (**1a**) (319 *m/z*), and discolin A (**1b**) (347 *m/z*). (**A**) EIC of 305 (blue), 319 (yellow), and 347 (purple). Peak for **1b** appears at 12.20 minutes and has low abundance, presence of **1b** confirmed with MSMS fragmentation, (**B**) MSMS fragmentation of **1,** with characteristic fragments of 105 and 201 *m/z*, (**C**) MSMS fragmentation of **1a,** with characteristic fragments of 105 and 215 *m/z*, and (**D**) MSMS fragmentation of **1b**, with characteristic fragments of 105 and 243 *m/z*.

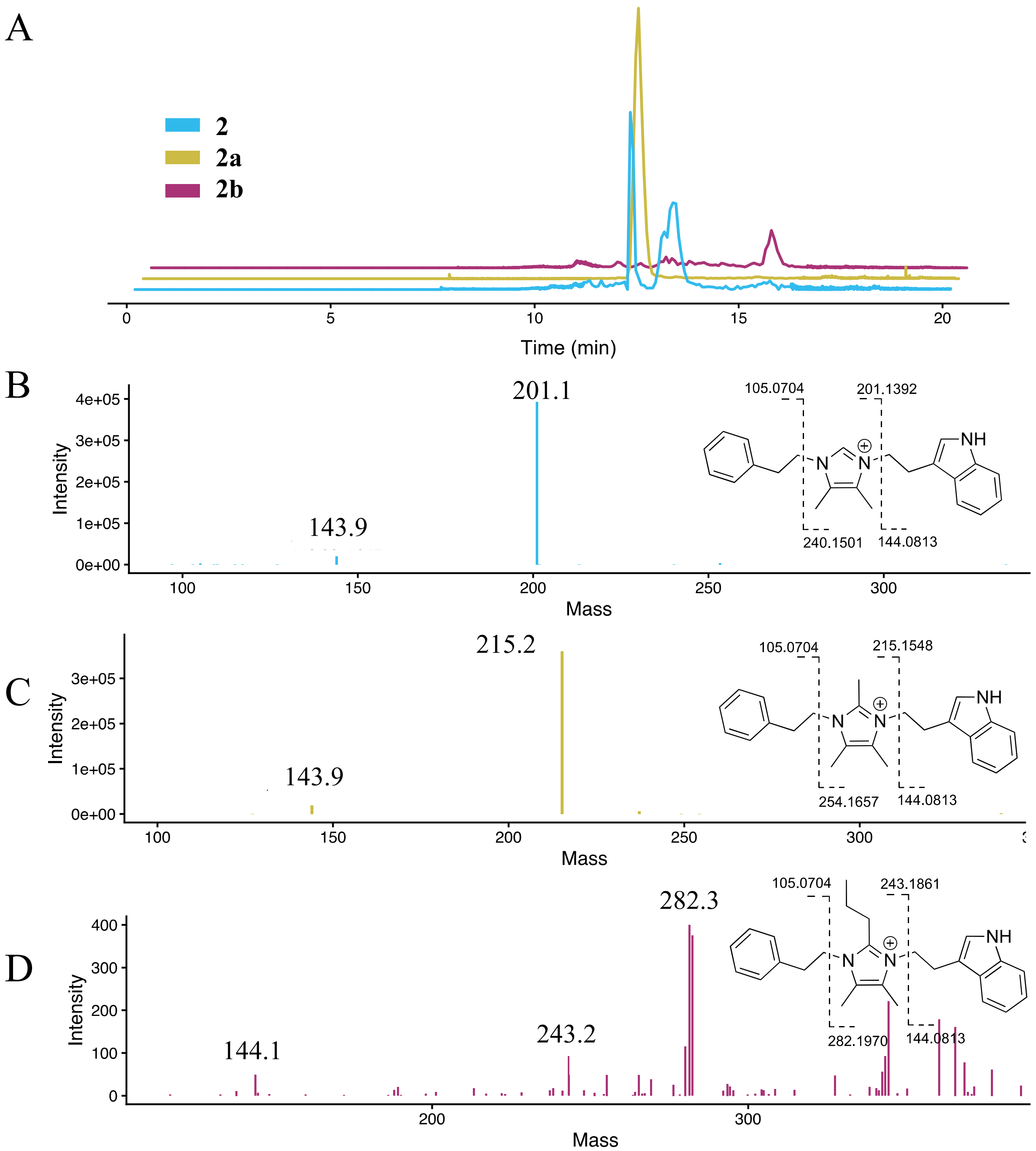

Figure S32. Mass spectroscopy of phethethylamine and tryptamine non-enzymatic reactions to generate bacillimidazole E (**2**) (344 *m/z*), bacillimidazole F (**2a**) (358 *m/z*), and discolin B (**2b**) (386 *m/z*). (**A**) EIC of 344 (blue), 358 (yellow), and 386 (purple) (peak for **2b** appears at 15.40 minutes), (**B**) MSMS fragmentation of **2**, with characteristic fragments of 144 and 201 *m/z*, (**C**) MSMS fragmentation of **2a,** with characteristic fragments of 144 and 215 *m/z*, and (**D**) MSMS fragmentation of **2b**, with characteristic fragments of 144, 243 and 282 *m/z*.

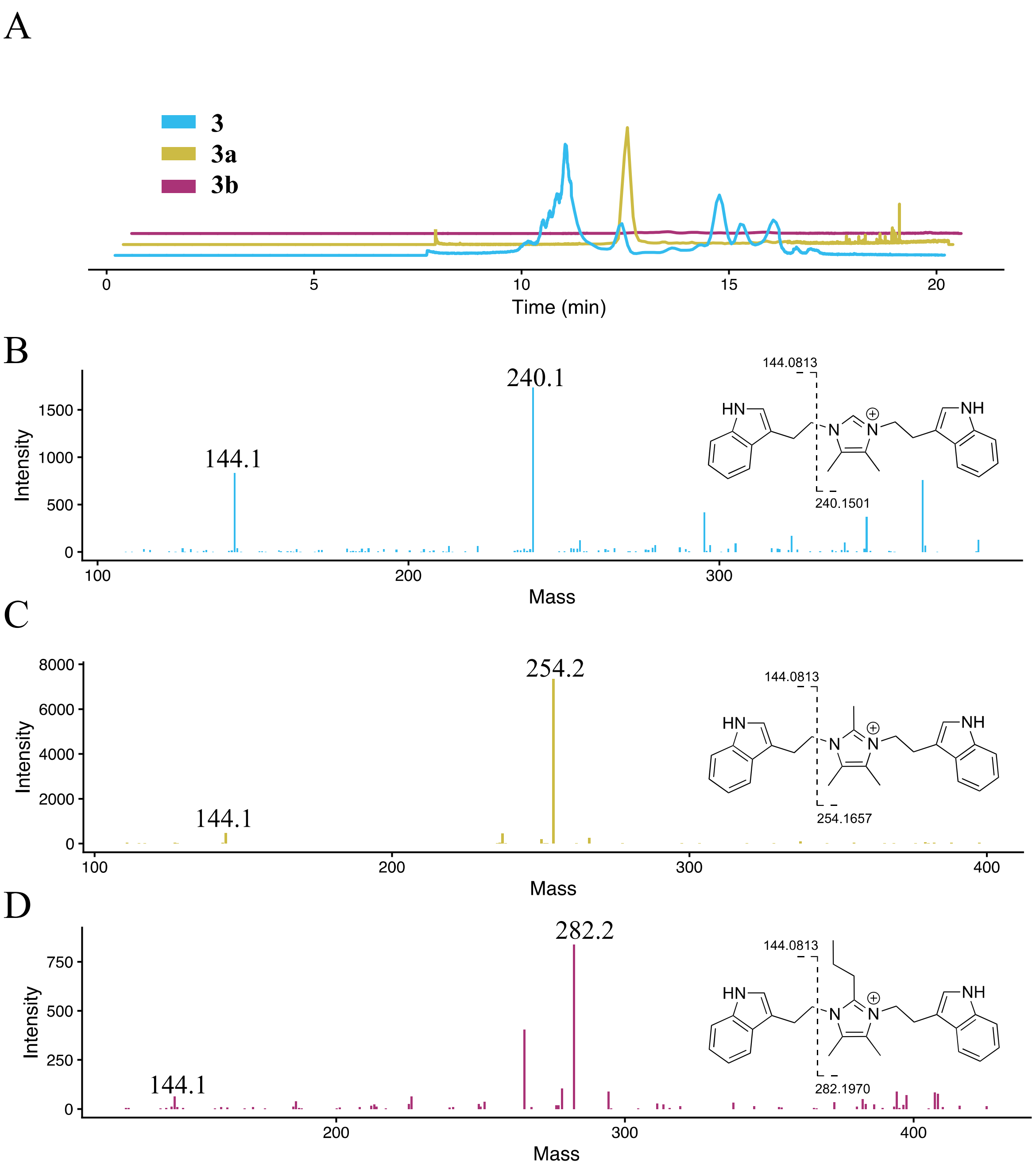

Figure S33. Mass spectroscopy of tryptamine non-enzymatic reactions to generate bacillimidazole C (**3**) (383 *m/z*), bacillimidazole D (**3a**) (397 *m/z*), and discolin D/bacillimidazole G (**3b**) (425 *m/z*), Peak for **3b** appears at 13.13 minutes and has low abundance, presence of **3b** confirmed with MSMS fragmentation. (**A**) EIC of 383 (blue), 397 (yellow), and 425 (purple), (**B**) MSMS fragmentation of **3**, with characteristic fragments of 144 and 240 *m/z*, (**C**) MSMS fragmentation of **3a,** with characteristic fragments of 144 and 254 *m/z*, and (**D**) MSMS fragmentation of **3b**, with characteristic fragments of 144 and 282 *m/z*.

A

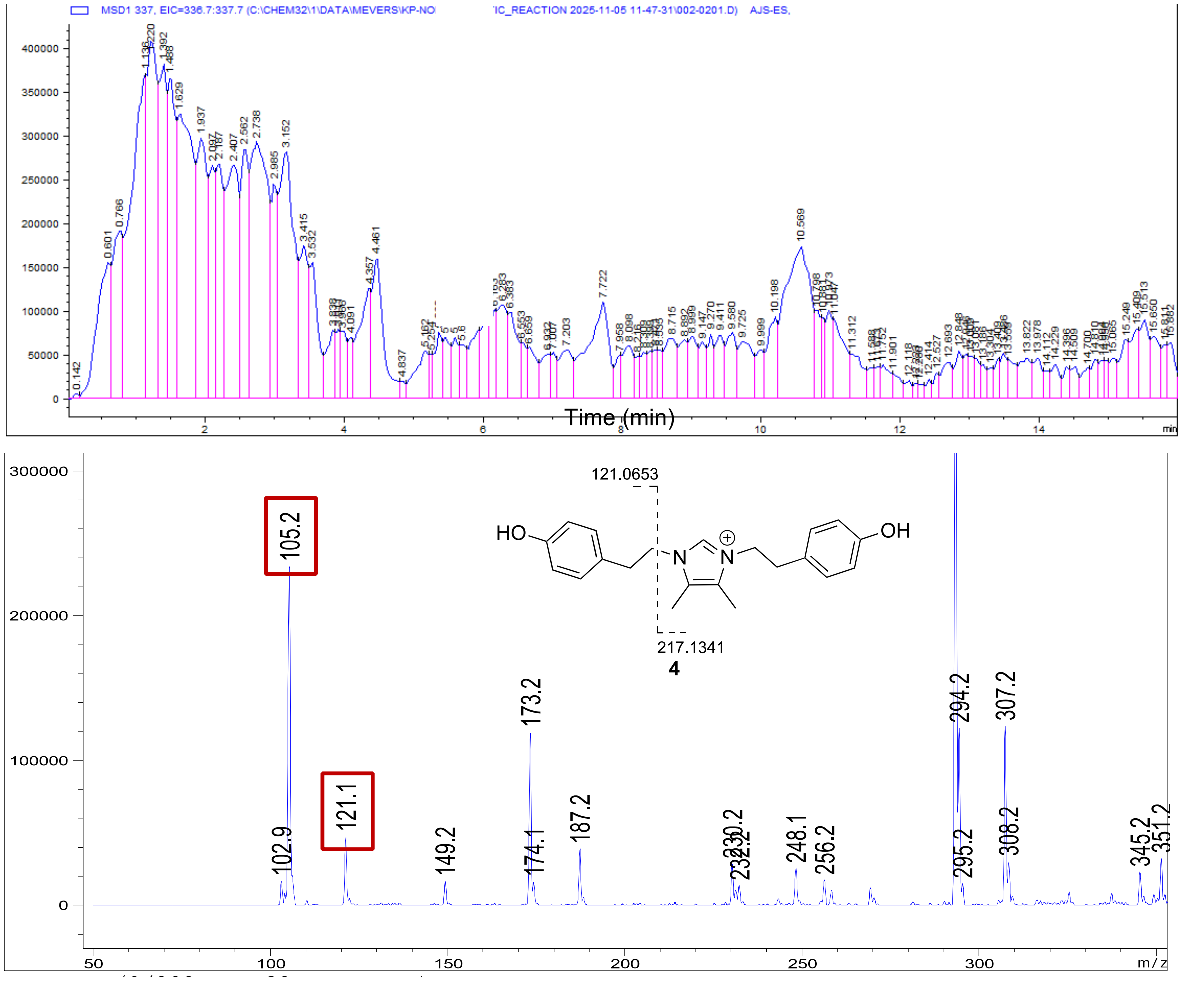
MSD1 337, EIC=336.7:337.7 (C:\CHEM32\1\DATA\MEVERS\KP-NO[illegible]IC_REACTION 2025-11-05 11-47-31\002-0201.D) AJS-ES,
Time (min)
121.0653
217.1341
4
HO
OH
105.2
121.1
102.9
149.2
173.2
174.1
187.2
230.2
232.2
248.1
256.2
294.2
295.2
307.2
308.2
345.2
351.2
m/z

B

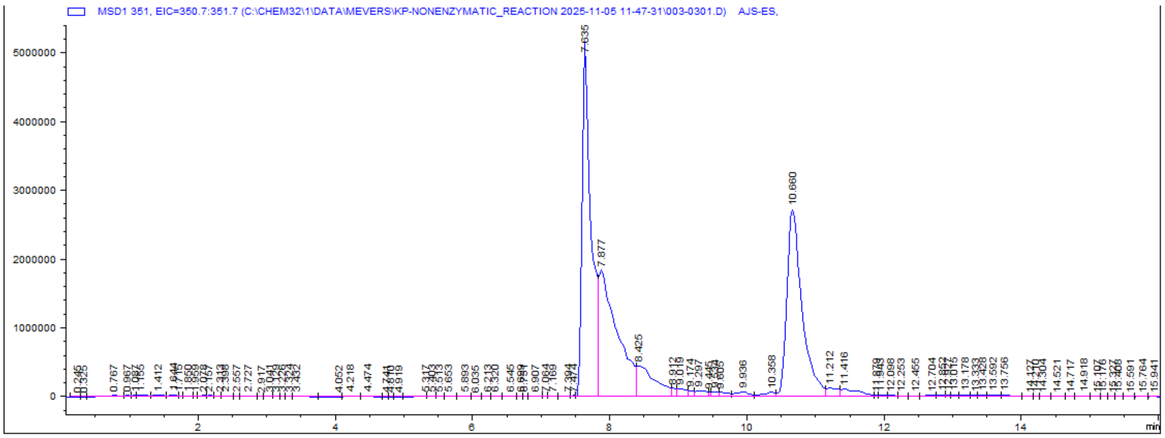
MSD1 351, EIC=350.7:351.7 (C:\CHEM32\1\DATA\MEVERS\KP-NONENZYMATIC_REACTION 2025-11-05 11-47-31\003-0301.D) AJS-ES,
7.635
7.877
8.425
10.680
min

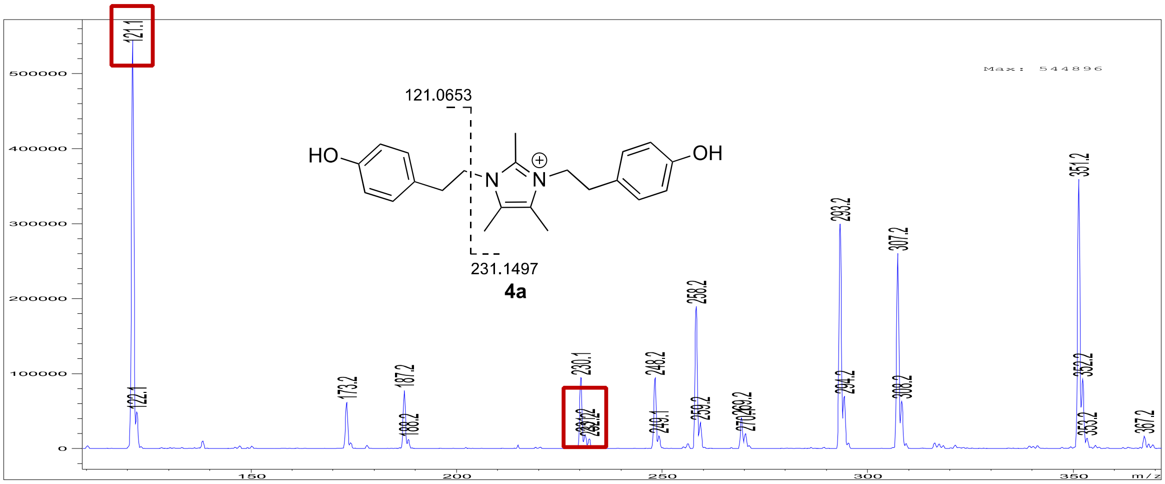

C

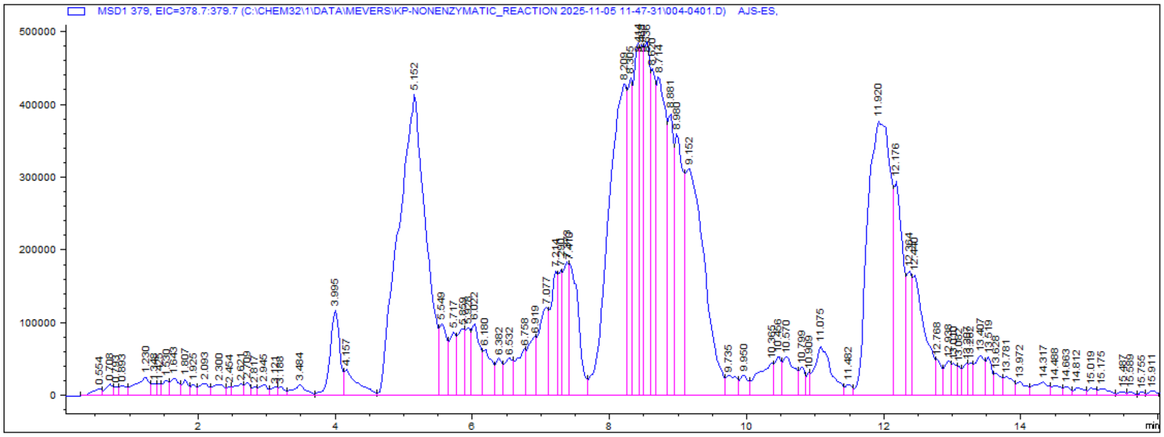

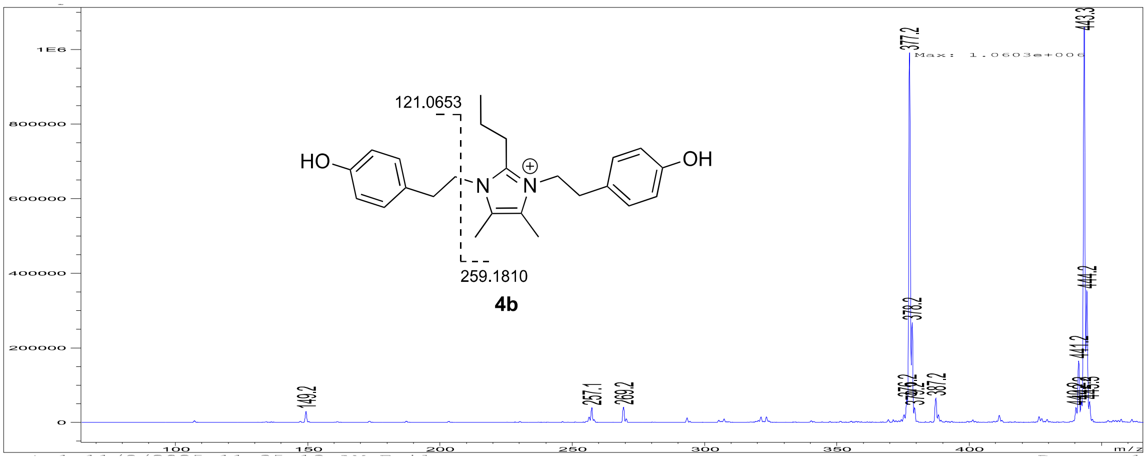

Figure S34. Mass spectroscopy of tyramine non-enzymatic reactions to (**4**) (337 *m/z*), (**4a**) (351 *m/z*), and (**4b**) (397 *m/z*). (**A**) EIC of 337 (top panel) and pseudo-MSMS fragmentation (bottom panel) with characteristic fragments of 121 and 105 *m/z* (red boxes), (**B**) EIC of 351 (top panel) and pseudo-MSMS fragmentation (bottom panel) with characteristic fragments of 121 and 231 *m/z*, and (**C**) EIC of 397 (top panel) and pseudo-MSMS fragmentation without characteristic fragments of 121 and 259 *m/z*.

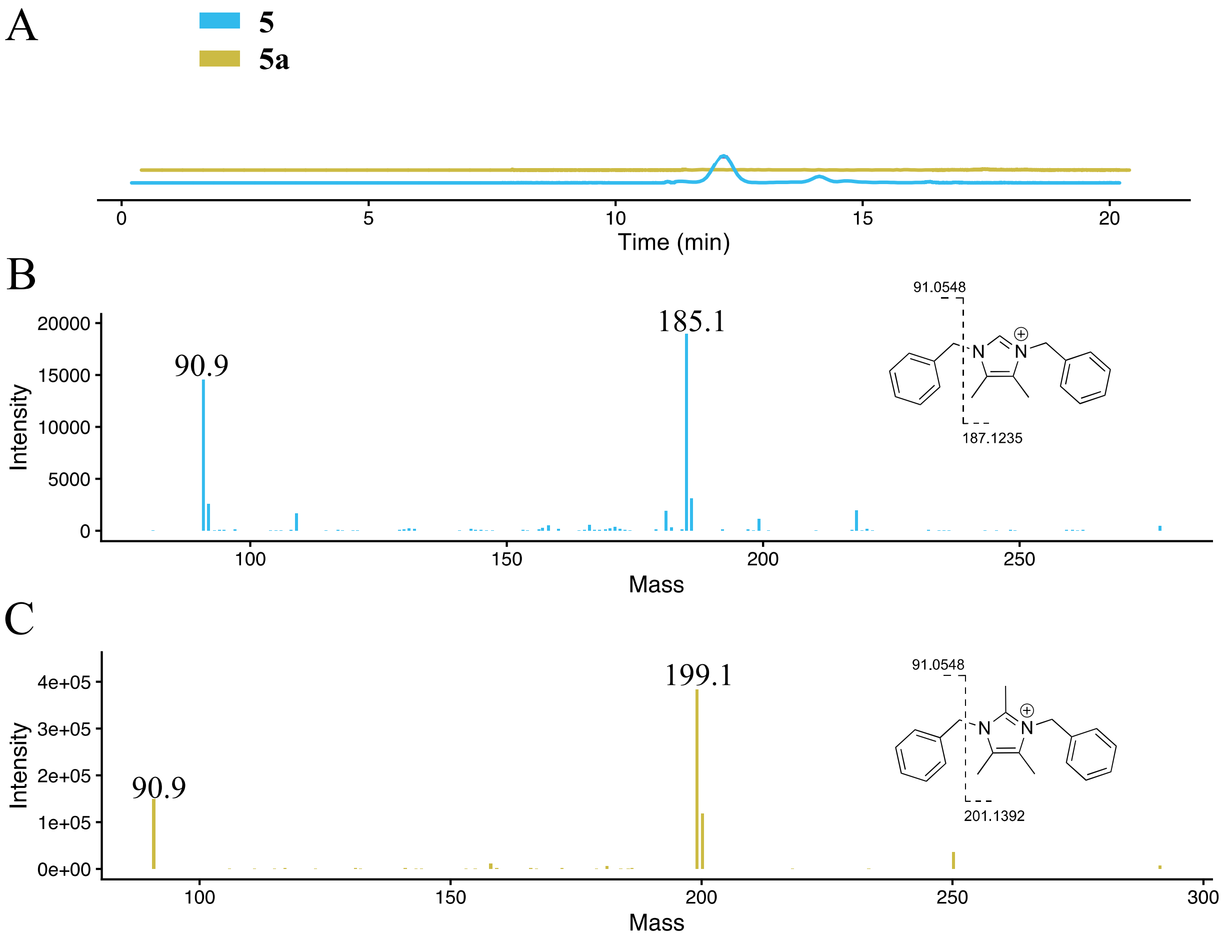
A
5
5a
0
5
10
15
20
Time (min)
B
Intensity
20000
15000
10000
5000
0
90.9
185.1
91.0548
187.1235
N
N
100
150
200
250
Mass
C
Intensity
4e+05
3e+05
2e+05
1e+05
0e+00
90.9
199.1
91.0548
201.1392
N
N
100
150
200
250
300
Mass

D

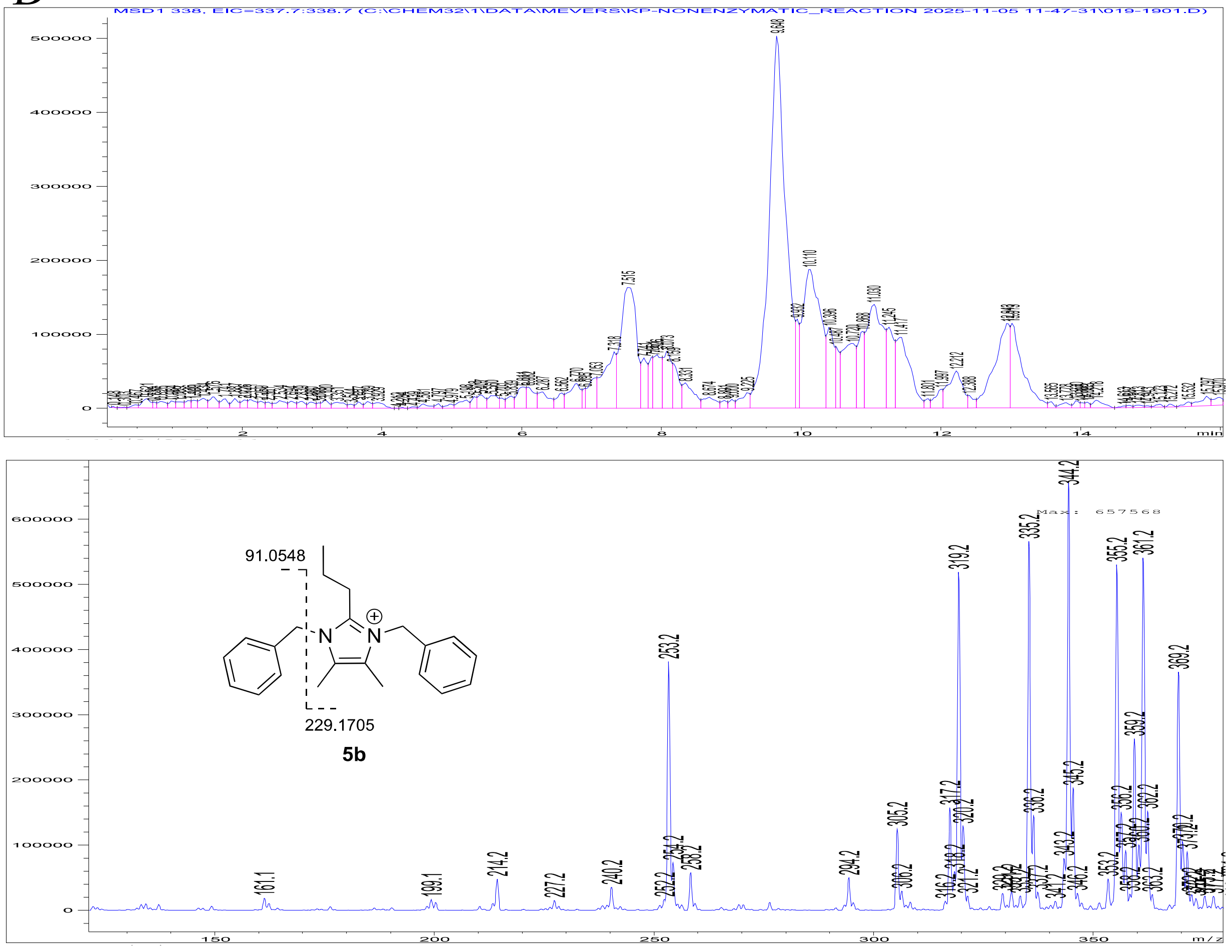

Figure S35. Mass spectroscopy of benzylamine non-enzymatic reactions to generate lepidiline A (**5**) (277 *m/z*), lepidiline B (**5a**) (*m/z* 291), and (**5b**) (319 *m/z*). (**A**) EIC of 277 (blue) and 291 (yellow), (**B**) MS2 fragmentation of **5**, with characteristic fragments of 91 and 185 *m/z*, (**C**) MS2 fragmentation of **5a,** with characteristic fragments of 91 and 199 *m/z*, and (**D**) EIC of 291 (top panel) and pseudo-MSMS fragmentation (bottom panel) without characteristic fragments.

A

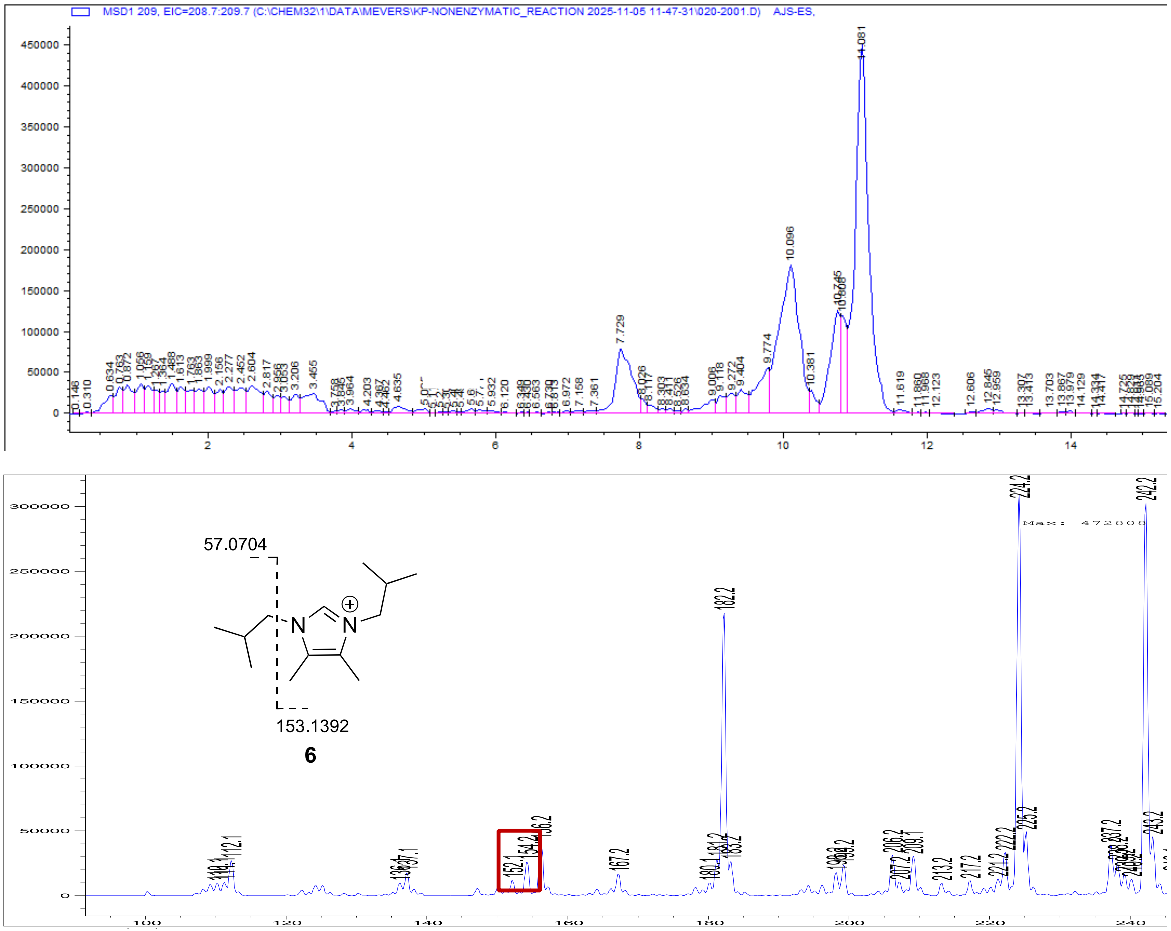
MSD1 209, EIC=208.7:209.7 (C:\CHEM32\1\DATA\MEVERS\KP-NONENZYMATIC_REACTION 2025-11-05 11-47-31\020-2001.D) AJS-ES,
7.729
10.096
11.081
57.0704
153.1392
6
182.2
224.2
242.2

B

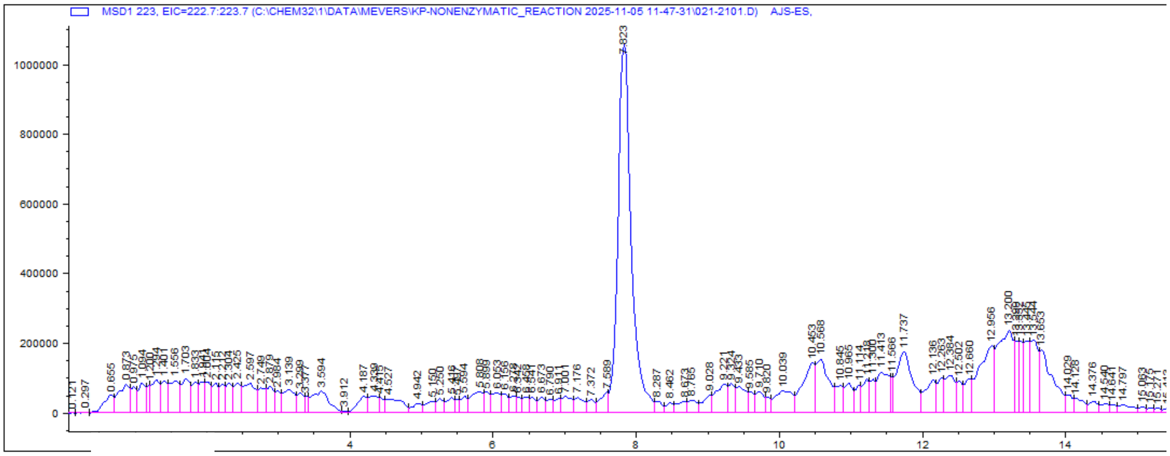
MSD1 223, EIC=222.7:223.7 (C:\CHEM32\1\DATA\MEVERS\KP-NONENZYMATIC_REACTION 2025-11-05 11-47-31\021-2101.D) AJS-ES,
7.823

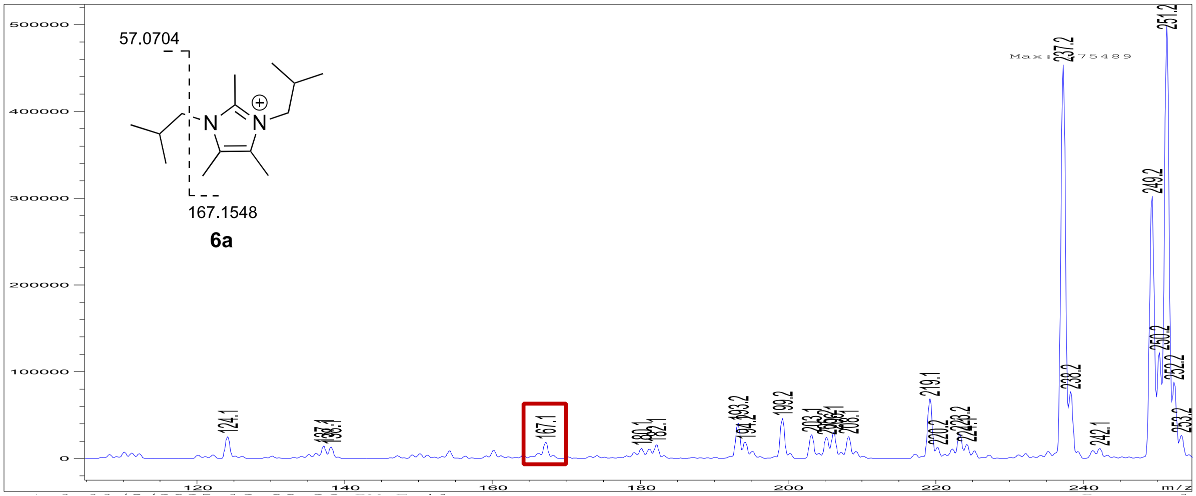

C

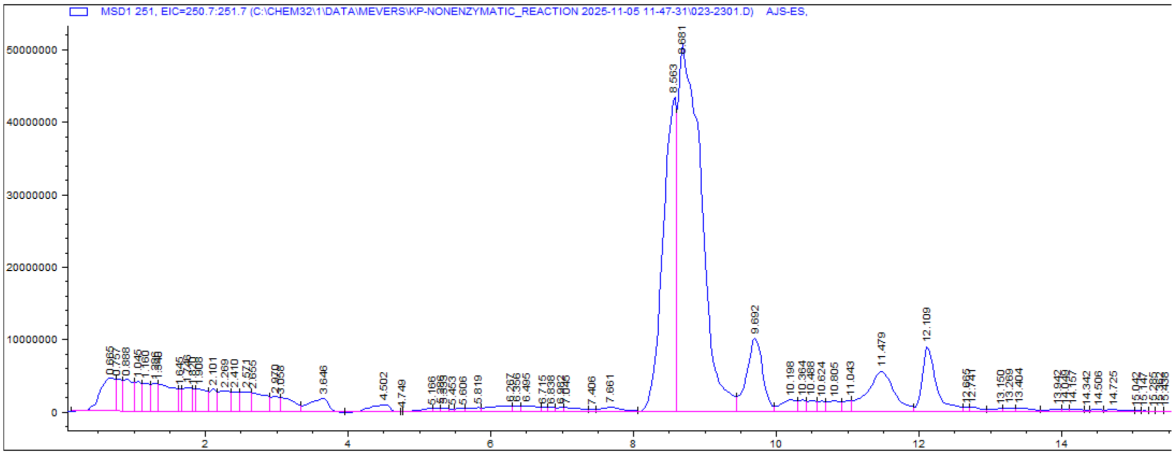

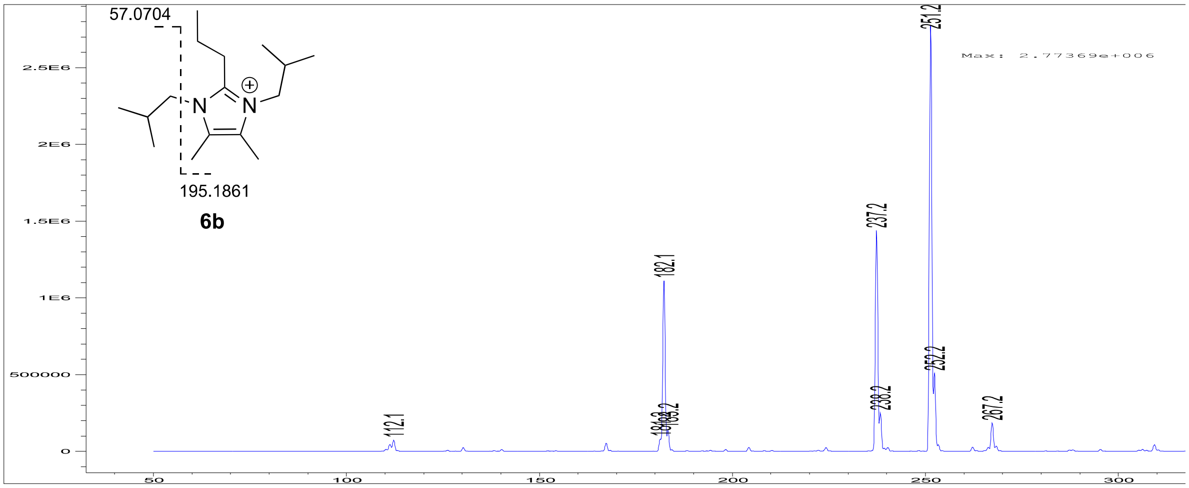

Figure S36. Mass spectroscopy of isobutyl amine non-enzymatic reactions (**6**) (209 *m/z*), (**6a**) (223 *m/z*), and (**6b**) (251 *m/z*). (**A**) EIC of 209 (top panel) and pseudo-MSMS fragmentation with characteristic fragments of 152 *m/z*, (**B**) EIC of 223 (top panel) and pseudo-MSMS fragmentation (bottom panel) with characteristic fragments of 167 *m/z*, and (**C**) EIC of 251 (top panel) and pseudo-MSMS fragmentation (bottom panel) without characteristic fragments.

A

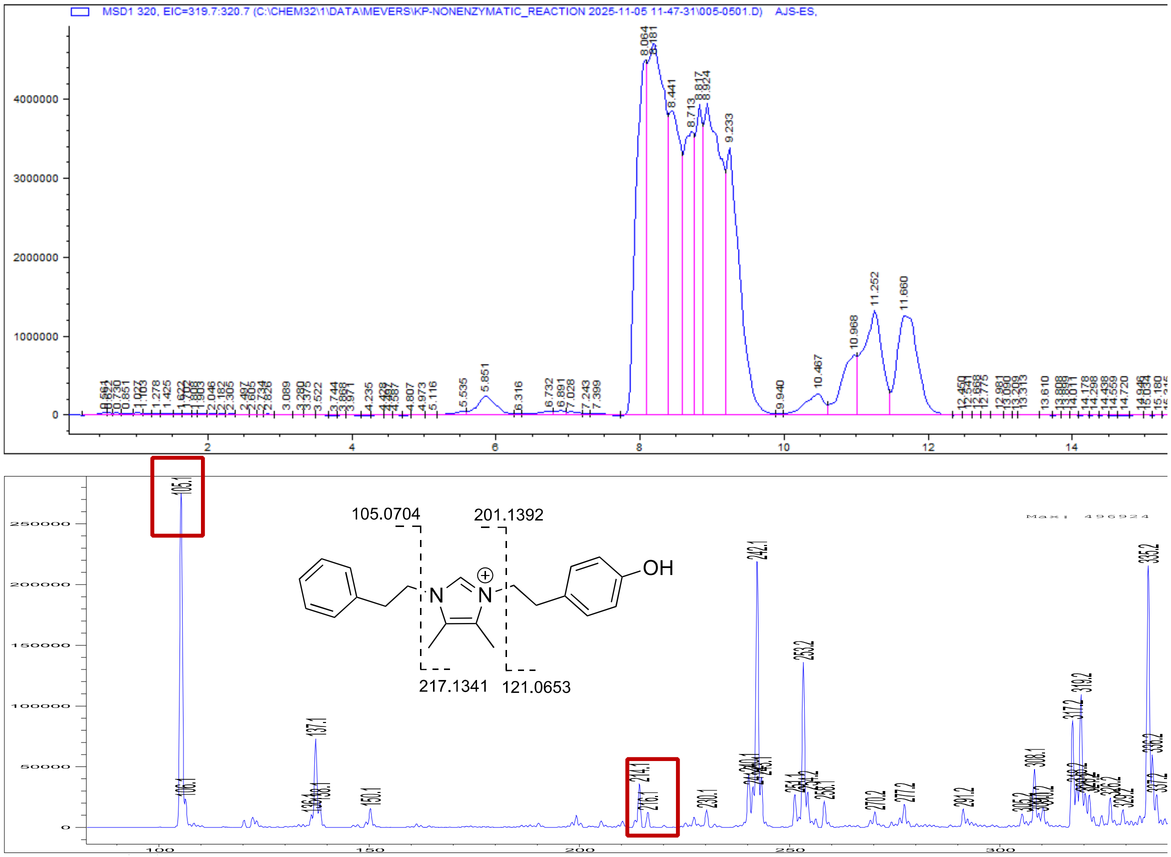
MSD1 320, EIC=319.7:320.7 (C:\CHEM32\1\DATA\MEVERS\KP-NONENZYMATIC_REACTION 2025-11-05 11-47-31\005-0501.D) AJS-ES,
105.0704
201.1392
217.1341
121.0653
OH
N
N

B

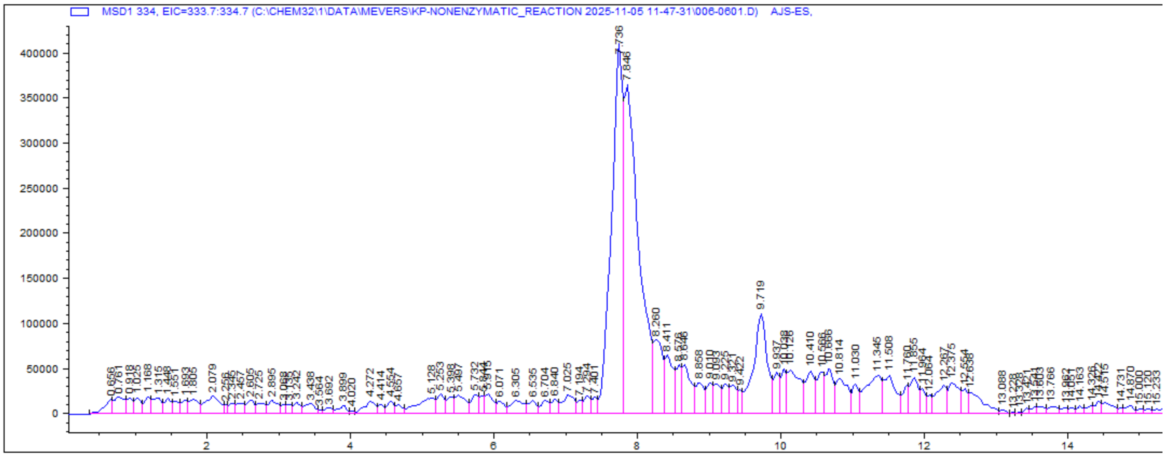
MSD1 334, EIC=333.7:334.7 (C:\CHEM32\1\DATA\MEVERS\KP-NONENZYMATIC_REACTION 2025-11-05 11-47-31\006-0601.D) AJS-ES,

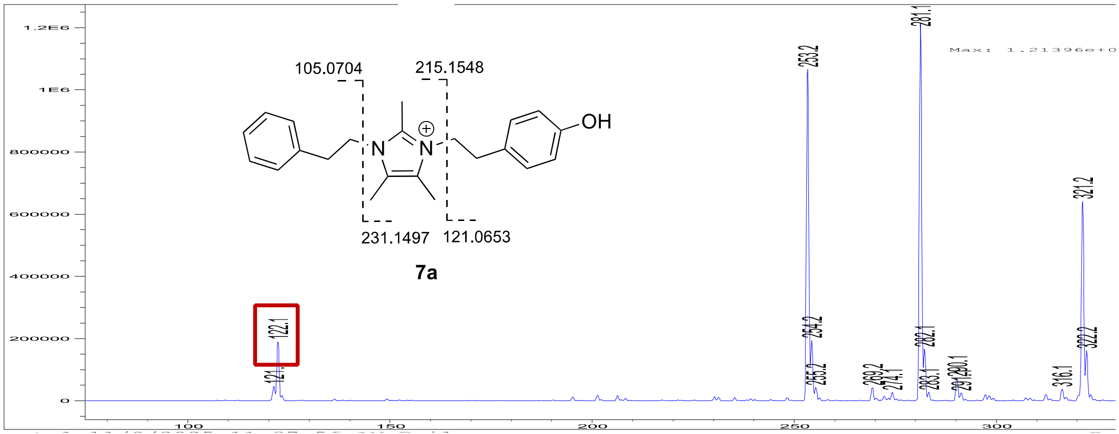

C

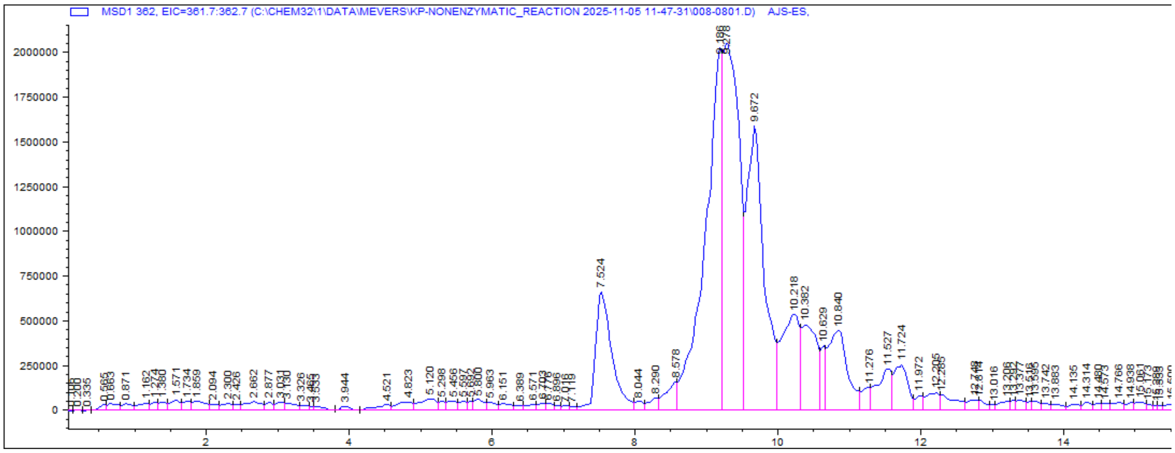

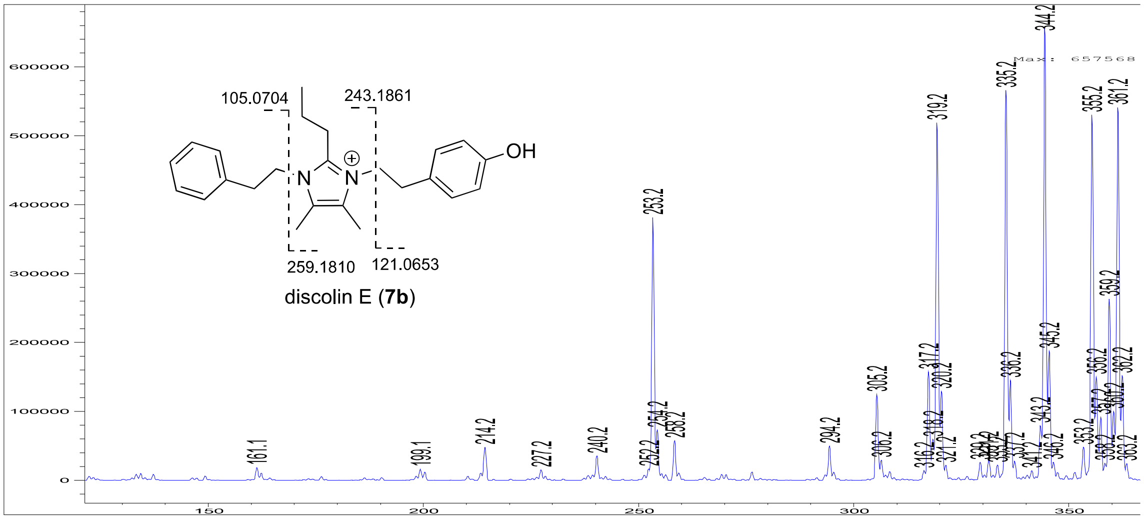

Figure S37. Mass spectroscopy of phenethylamine and tyramine non-enzymatic reactions to generate hydroxybacillimidazole A (**7**) (321 *m/z*), (**7a**) (335 *m/z*), and discolin E (**7b**) (363 *m/z*). (**A**) EIC of 321 (top panel) and pseudo-MSMS fragmentation (bottom panel) with characteristic fragments of 105 and 217 *m/z*, (**B**) EIC of 335 (top panel) and pseudo-MSMS fragmentation (bottom panel) with characteristic fragment of 121 *m/z*, and (**C**) EIC of 363 (top panel) and pseudo-MSMS fragmentation (bottom panel) without characteristic fragments.

A

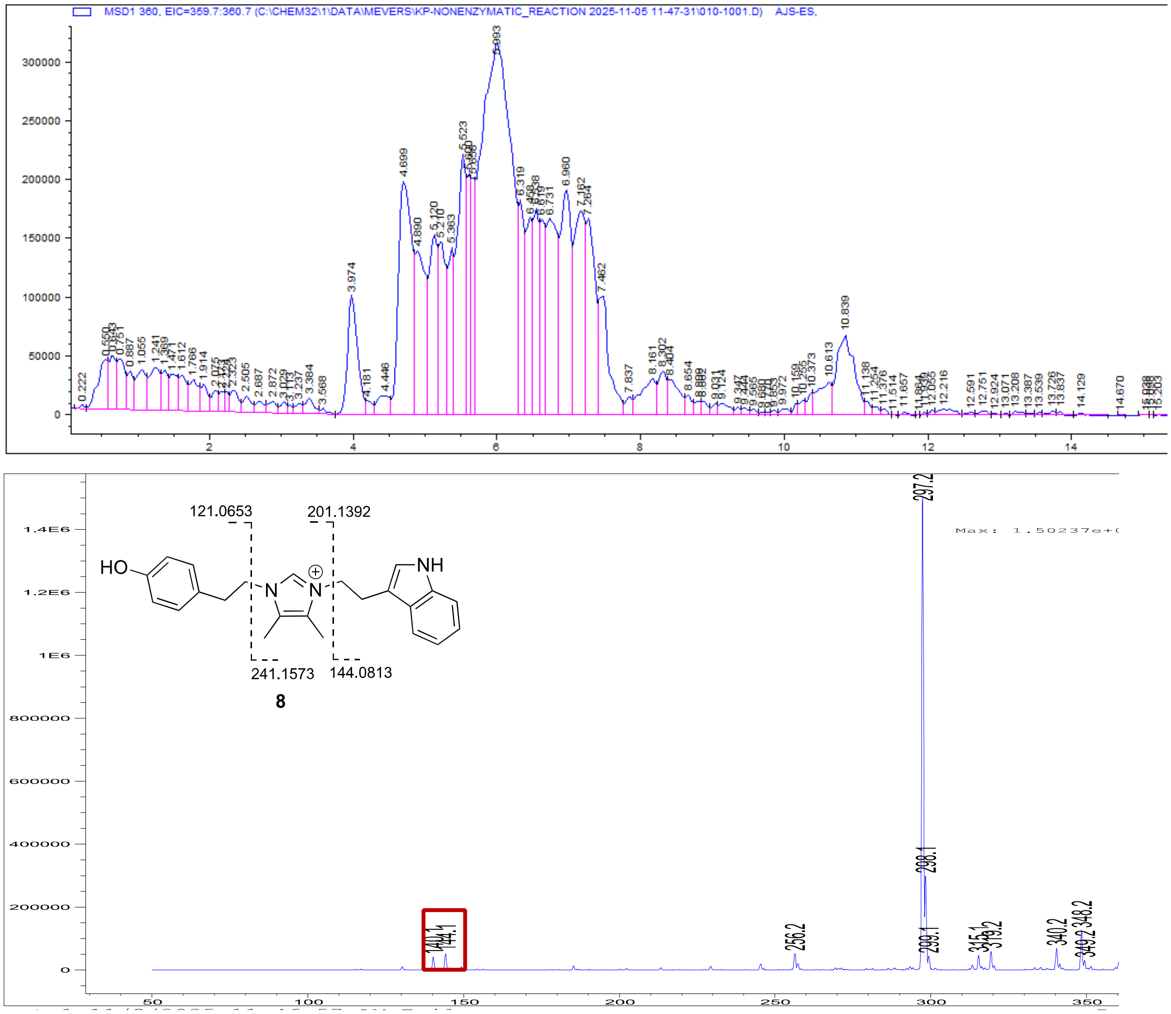
MSD1 360, EIC=359.7:360.7 (C:\CHEM32\1\DATA\MEVERS\KP-NONENZYMATIC_REACTION 2025-11-05 11-47-31\010-1001.D) AJS-ES,
121.0653
201.1392
HO
NH
N
N
241.1573
144.0813
8
Max: 1.50237e+0
297.2
298.1
256.2
140.1
144.1

B

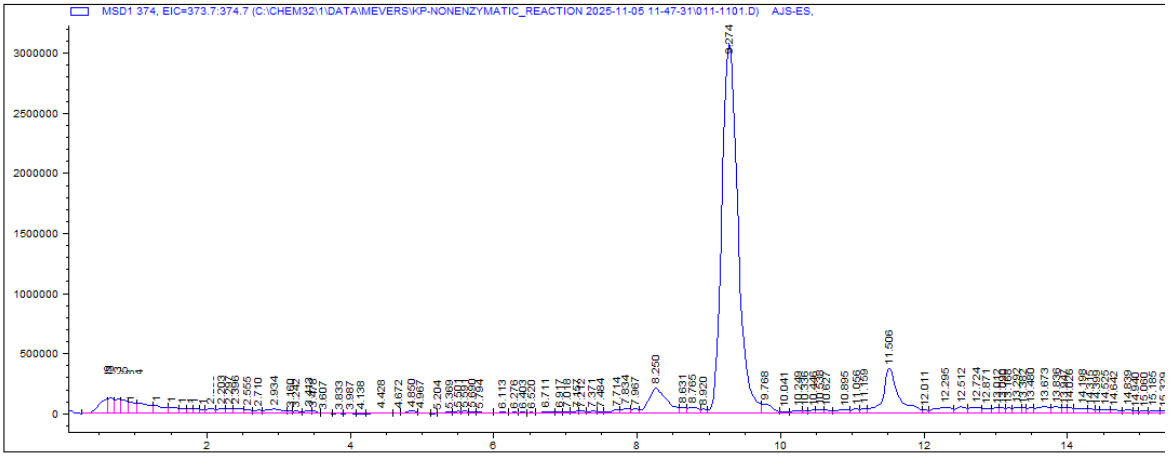
MSD1 374, EIC=373.7:374.7 (C:\CHEM32\1\DATA\MEVERS\KP-NONENZYMATIC_REACTION 2025-11-05 11-47-31\011-1101.D) AJS-ES,
9.274
11.506
8.250

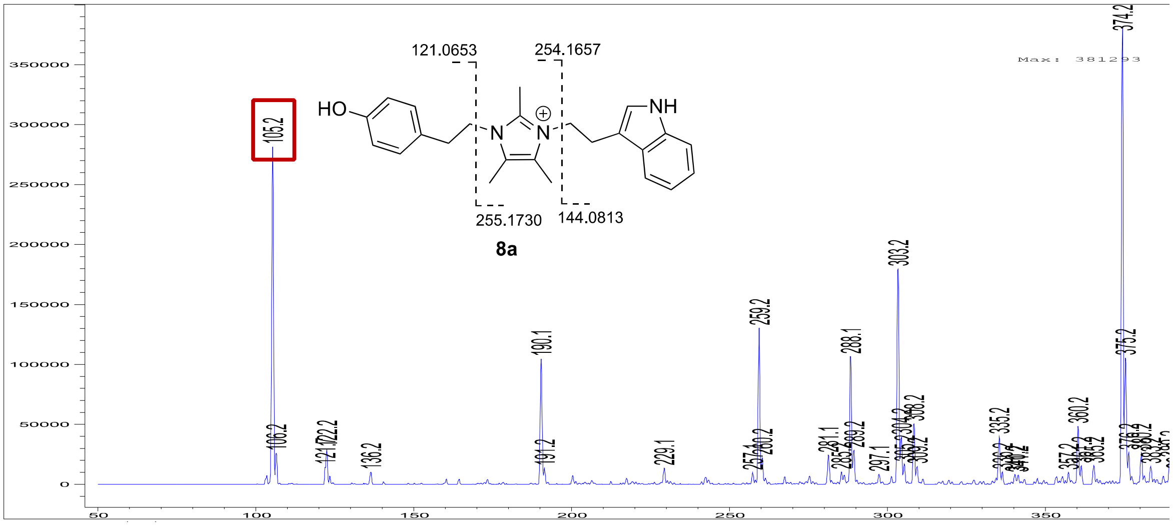

C

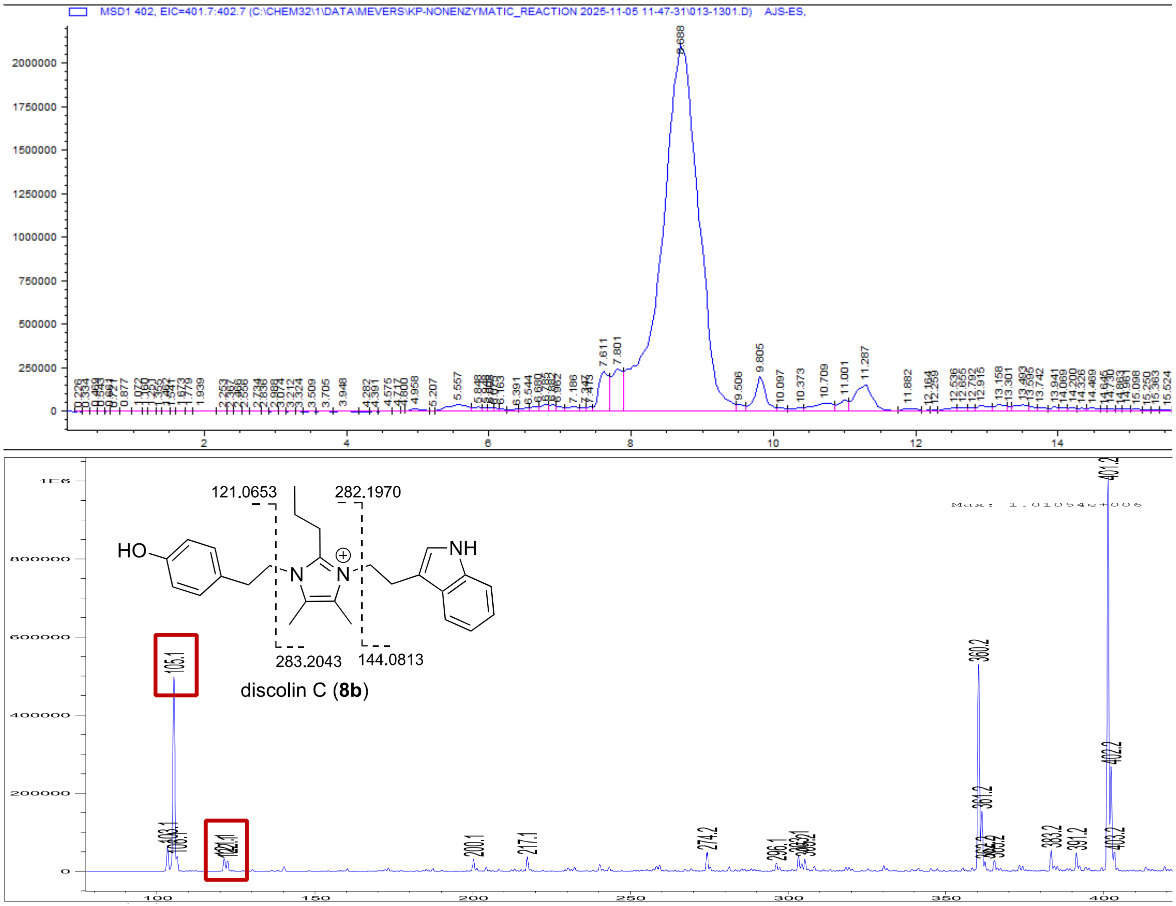

Figure S38. Mass spectroscopy of tryptamine and tyramine non-enzymatic reactions to generate (**8**) (360 *m/z*), (**8a**) (374 *m/z*), and discolin C (**8b**) (402 *m/z*). (**A**) EIC of 360 (top panel) and pseudo-MSMS fragmentation (bottom panel) with characteristic fragments of 144 *m/z*; (**B**) EIC of 374 (top panel) and pseudo-MSMS fragmentation (bottom panel) with characteristic fragments of 105 and 121 *m/z*, and (**C**) EIC of 402 (top pane) and pseudo-MSMS fragmentation (bottom panel) with characteristic fragments of 105 and 121 *m/z*.

A

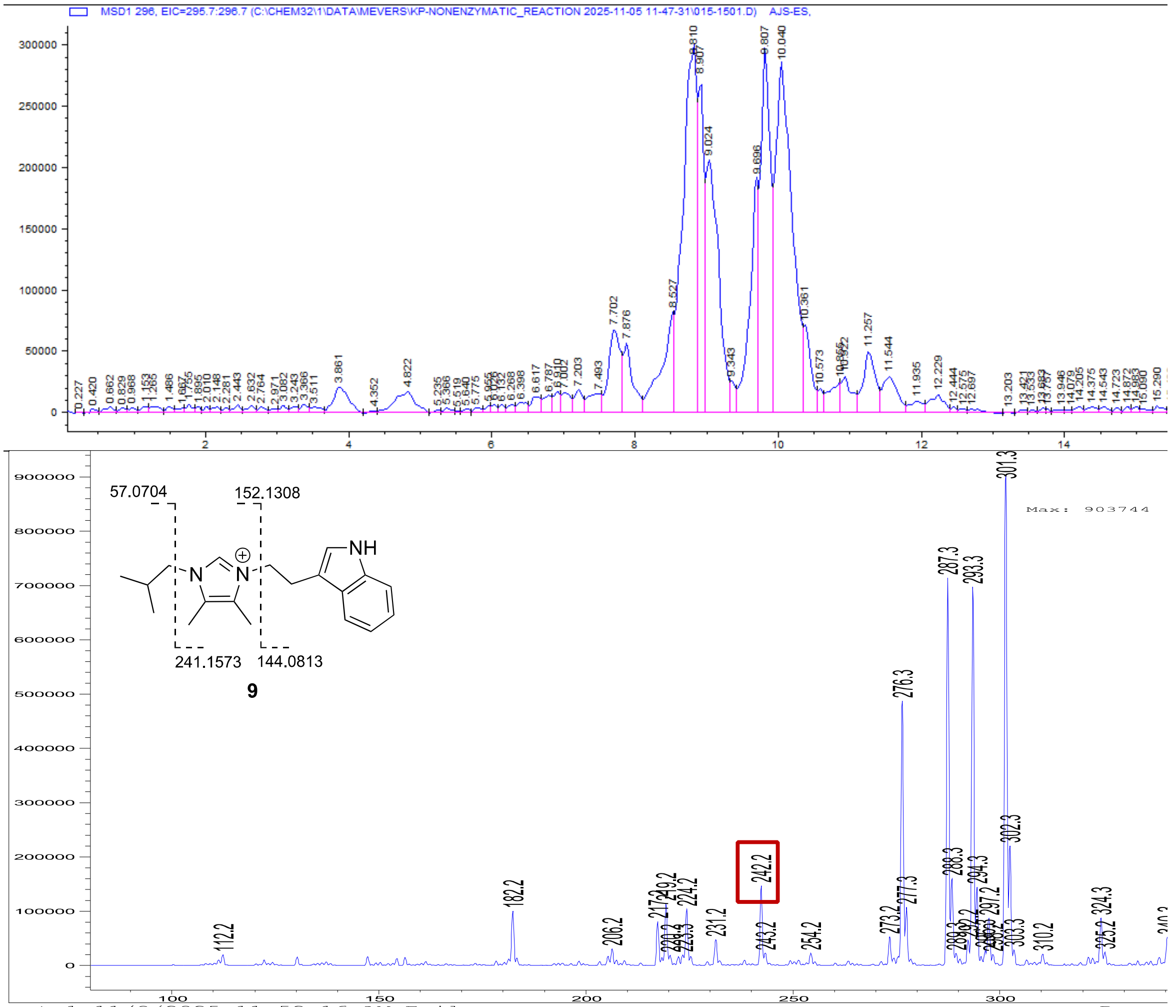
MSD1 296, EIC=295.7:296.7 (C:\CHEM32\1\DATA\MEVERS\KP-NONENZYMATIC_REACTION 2025-11-05 11-47-31\015-1501.D) AJS-ES,
57.0704
152.1308
241.1573
144.0813
9
Max: 903744
242.2

B

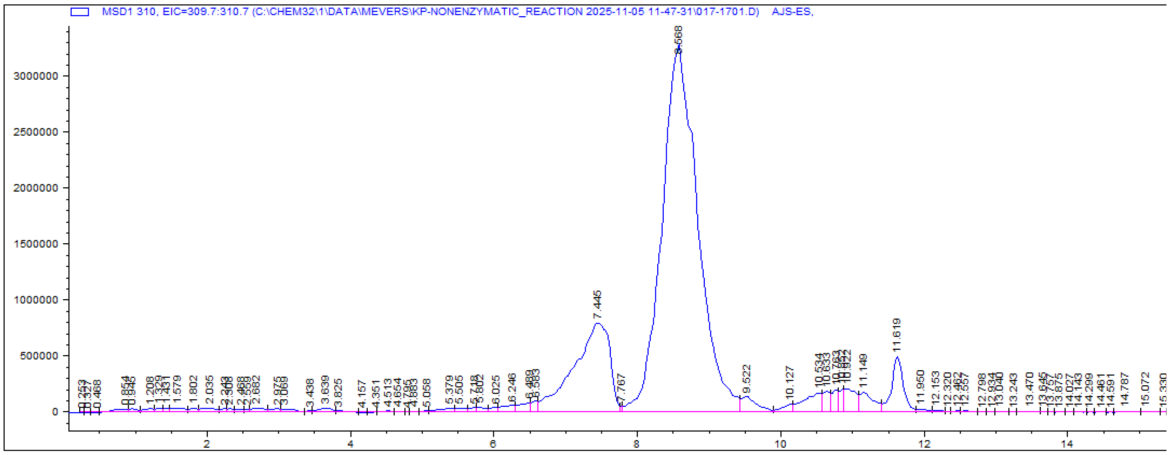
MSD1 310, EIC=309.7:310.7 (C:\CHEM32\1\DATA\MEVERS\KP-NONENZYMATIC_REACTION 2025-11-05 11-47-31\017-1701.D) AJS-ES,

C

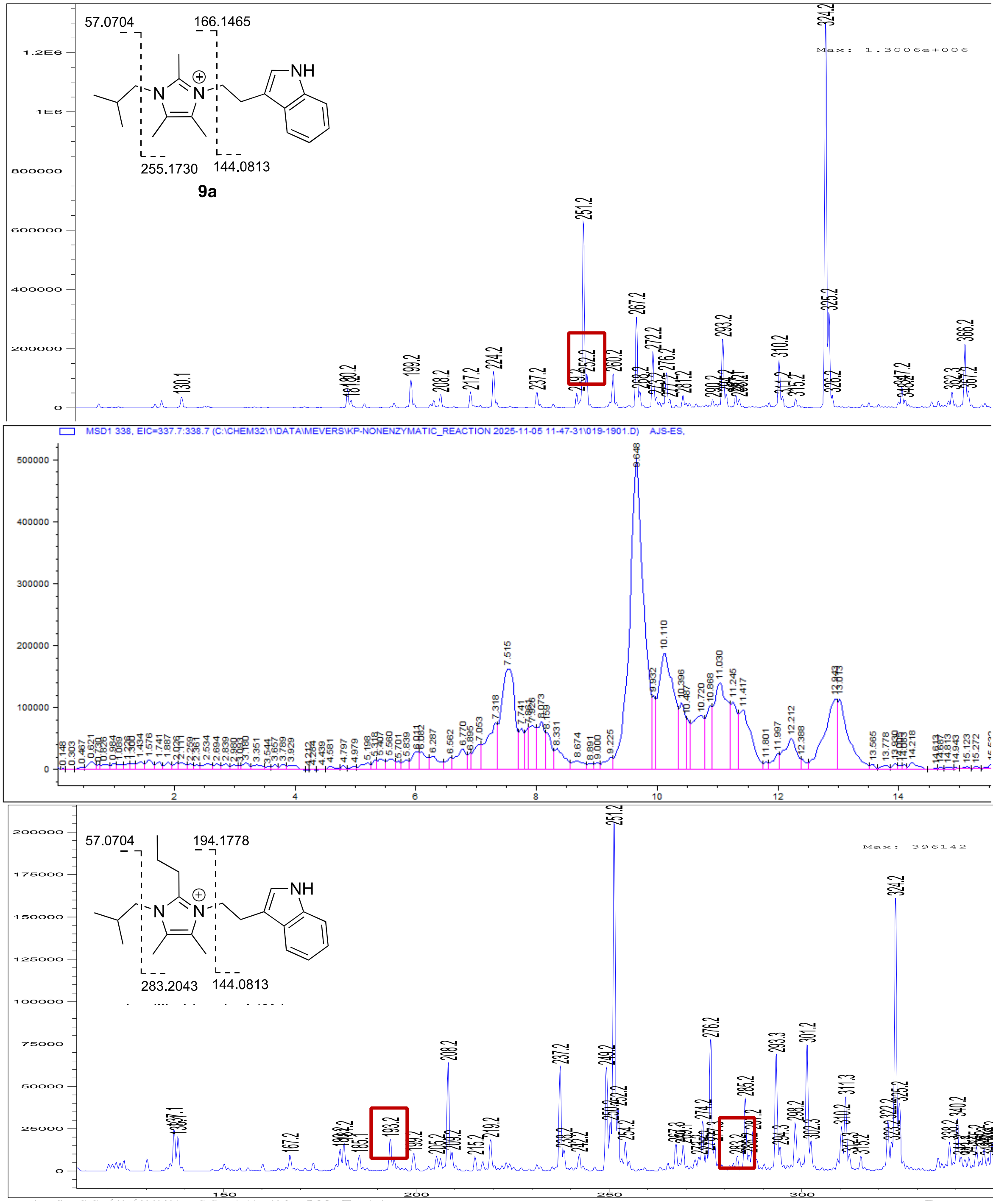

Figure S39. Mass spectroscopy of isobutyl amine and tryptamine non-enzymatic reactions to generate (**9**) (296 *m/z*), (**9a**) (310 *m/z*), and bacillimidazole J (**9b**) (338 *m/z*). (**A**) EIC of 296 (top panel) and pseudo-MSMS fragmentation with characteristic fragments of 242 *m/z*, (**B**) EIC of 310 (top panel) and pseudo-MSMS fragmentation (bottom panel) without characteristic fragments, and (**C**) EIC of 338 (top panel) and pseudo-MSMS fragmentation (bottom panel) without characteristic fragments.

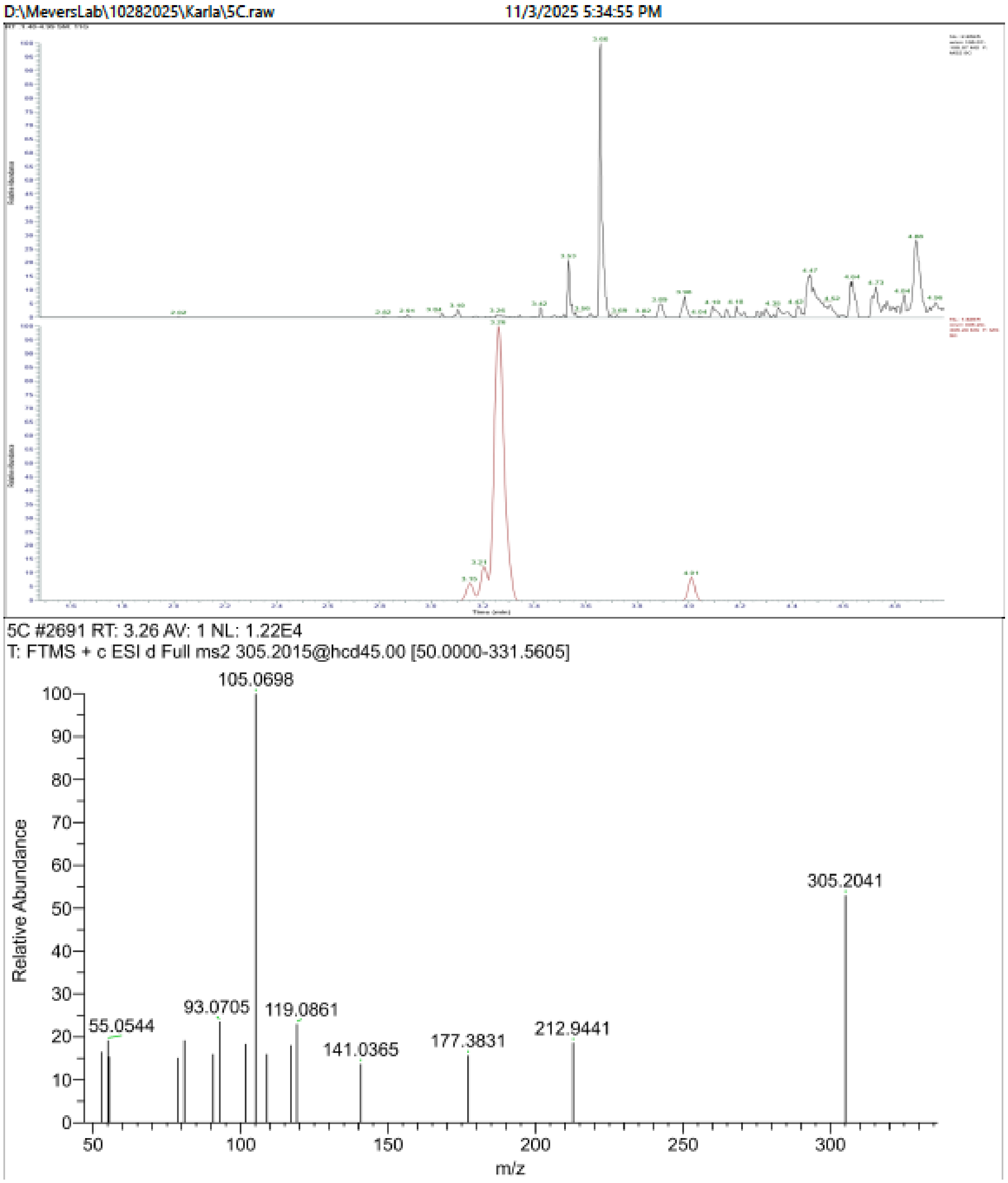

Figure S40. HR-LCMS analysis of egg collar 5C (site 1). The top chromatogram is an EIC of MSMS fragment data for 105.0700 *m/z* , the bottom chromatogram is an EIC of the parent spectra for 305.2015 *m/z*, and the spectra are for the fragmentation of 305.2041 *m/z*.

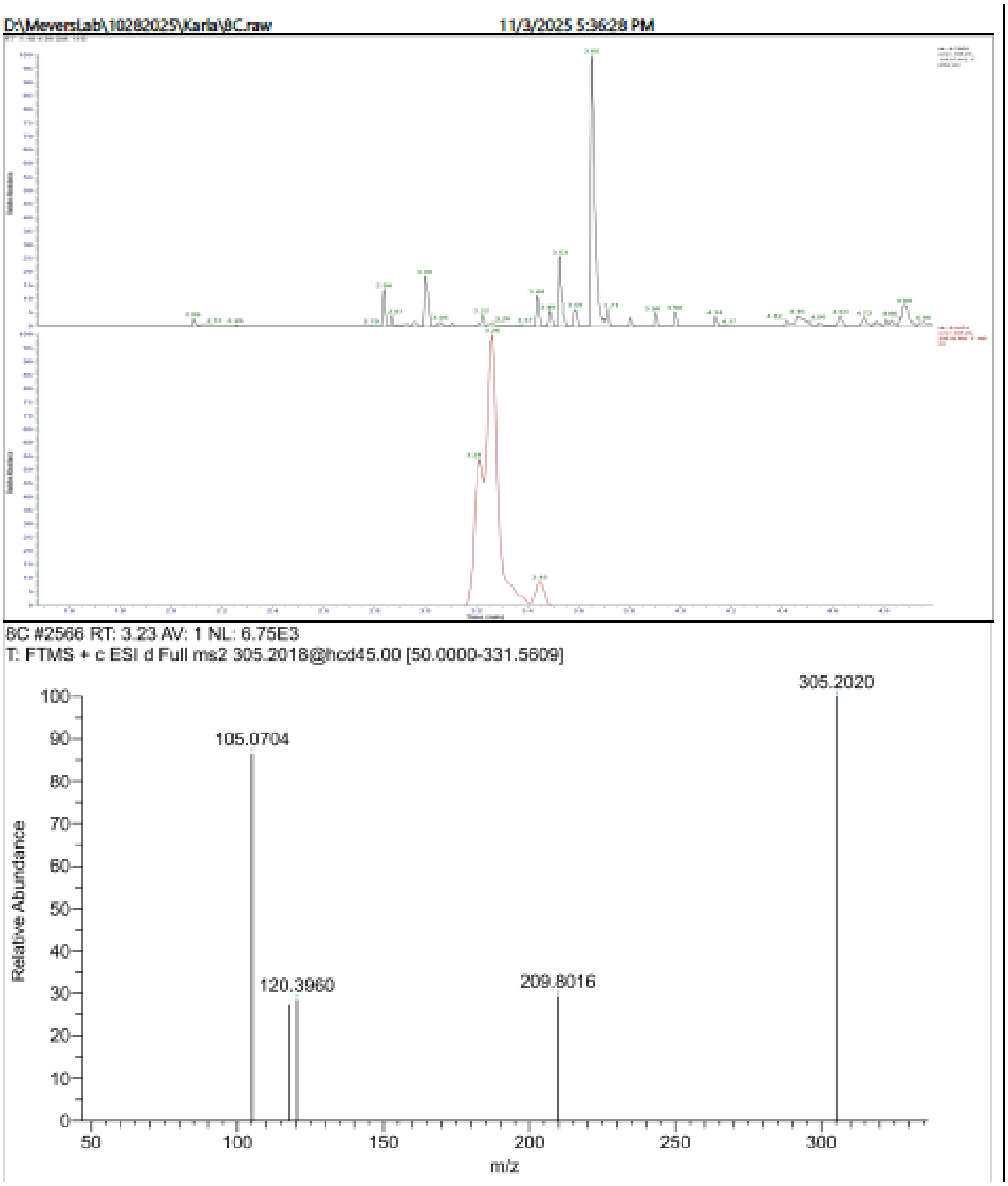

Figure S41. HR-LCMS analysis of egg collar 8C (site 1). The top chromatogram is an EIC of MSMS fragment data for 105.0700 *m/z*, the bottom chromatogram is an EIC of the parent spectra for 305.2018 *m/z*, and the spectra are for the fragmentation of 305.2041 *m/z*.

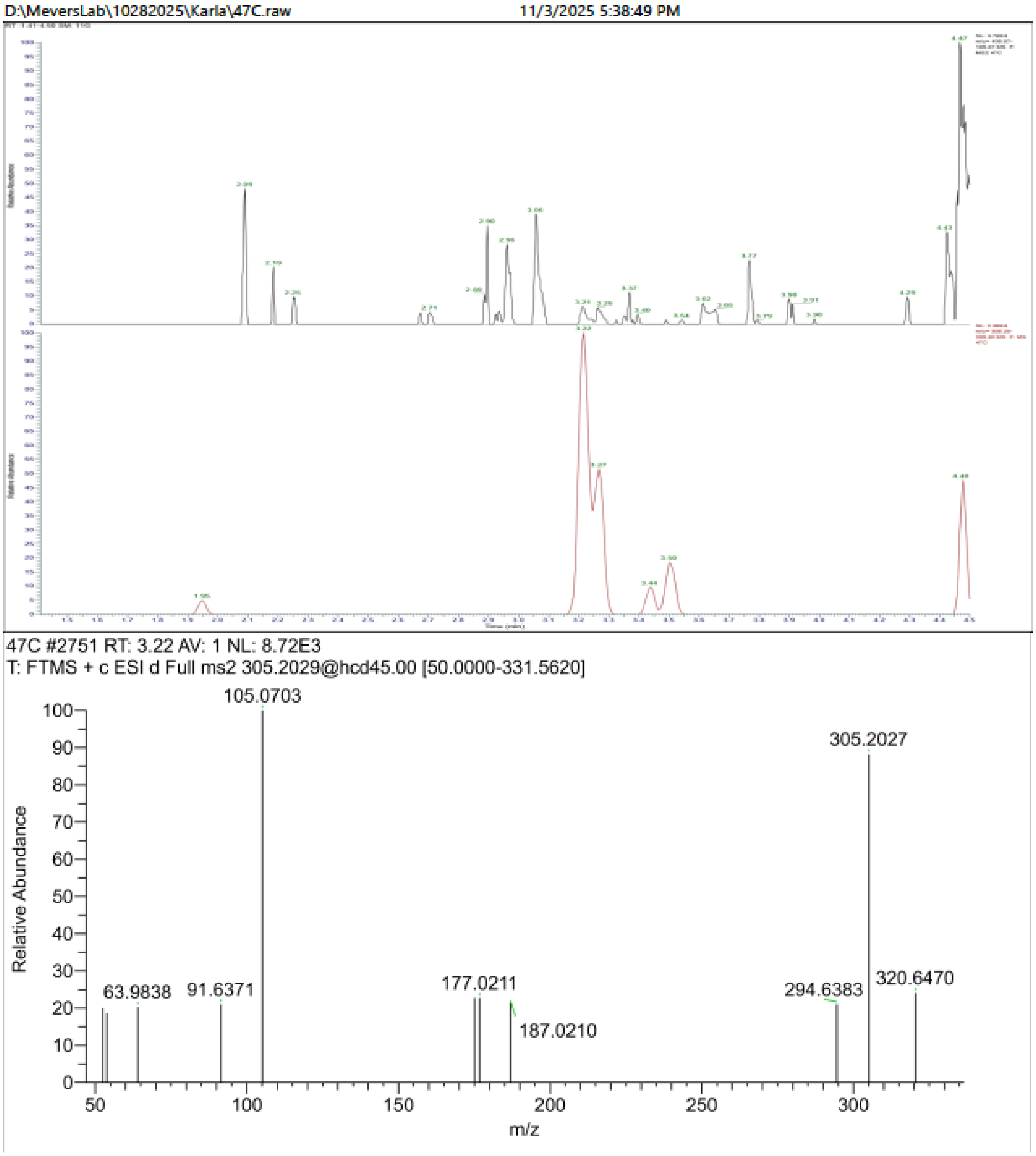

Figure S42. HR-LCMS analysis of egg collar 47C (site 2). The top chromatogram is an EIC of MSMS fragment data for 105.0700 *m/z*, the bottom chromatogram is an EIC of the parent spectra for 305.2029 *m/z*, and the spectra are for the fragmentation of 305.2041 *m/z*.

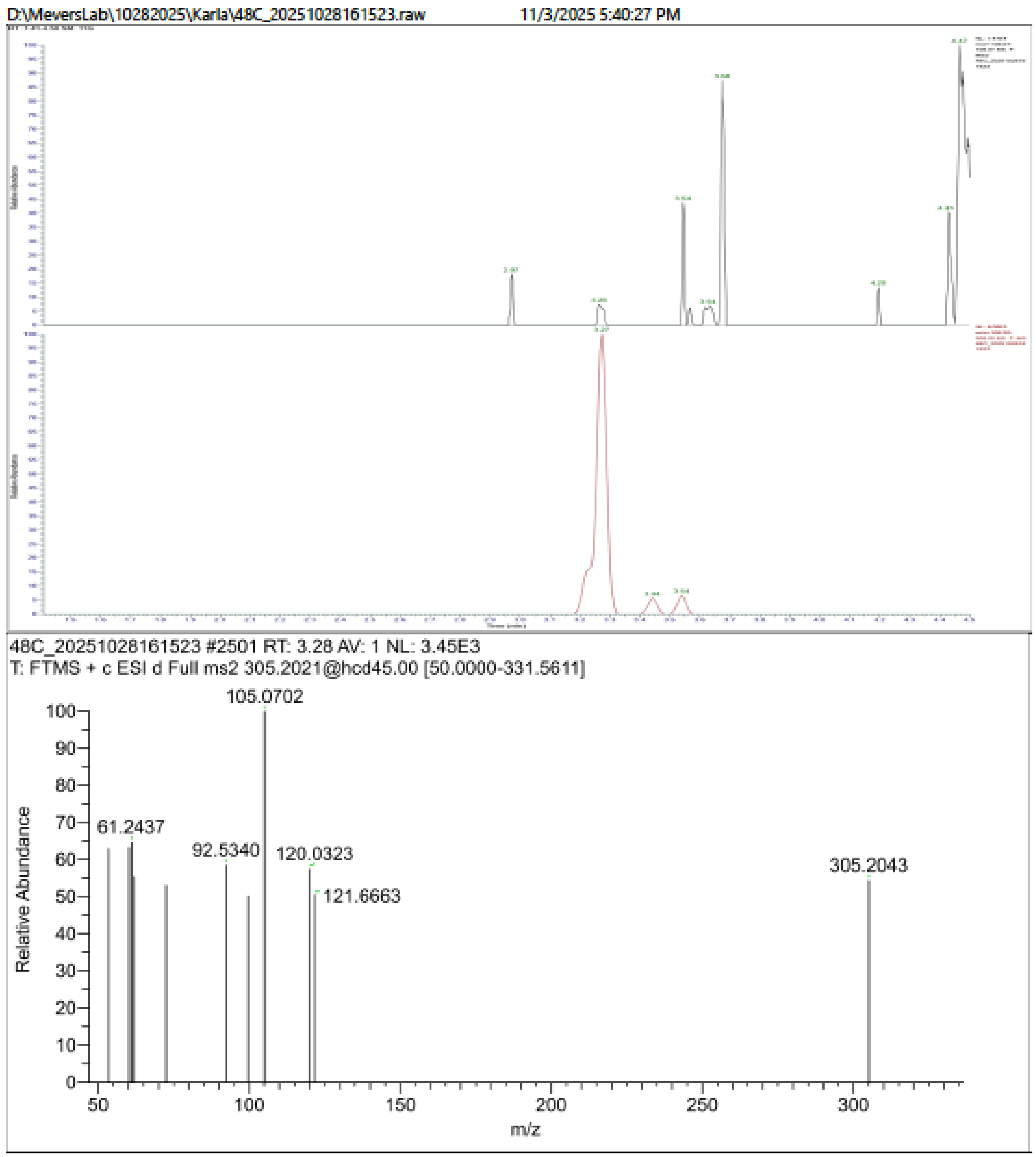

Figure S43. HR-LCMS analysis of egg collar 48C (site 2). The top chromatogram is an EIC of MSMS fragment data for 105.0700 *m/z*, the bottom chromatogram is an EIC of the parent spectra for 305.2021 *m/z*, and the spectra are for the fragmentation of 305.2041 *m/z.*

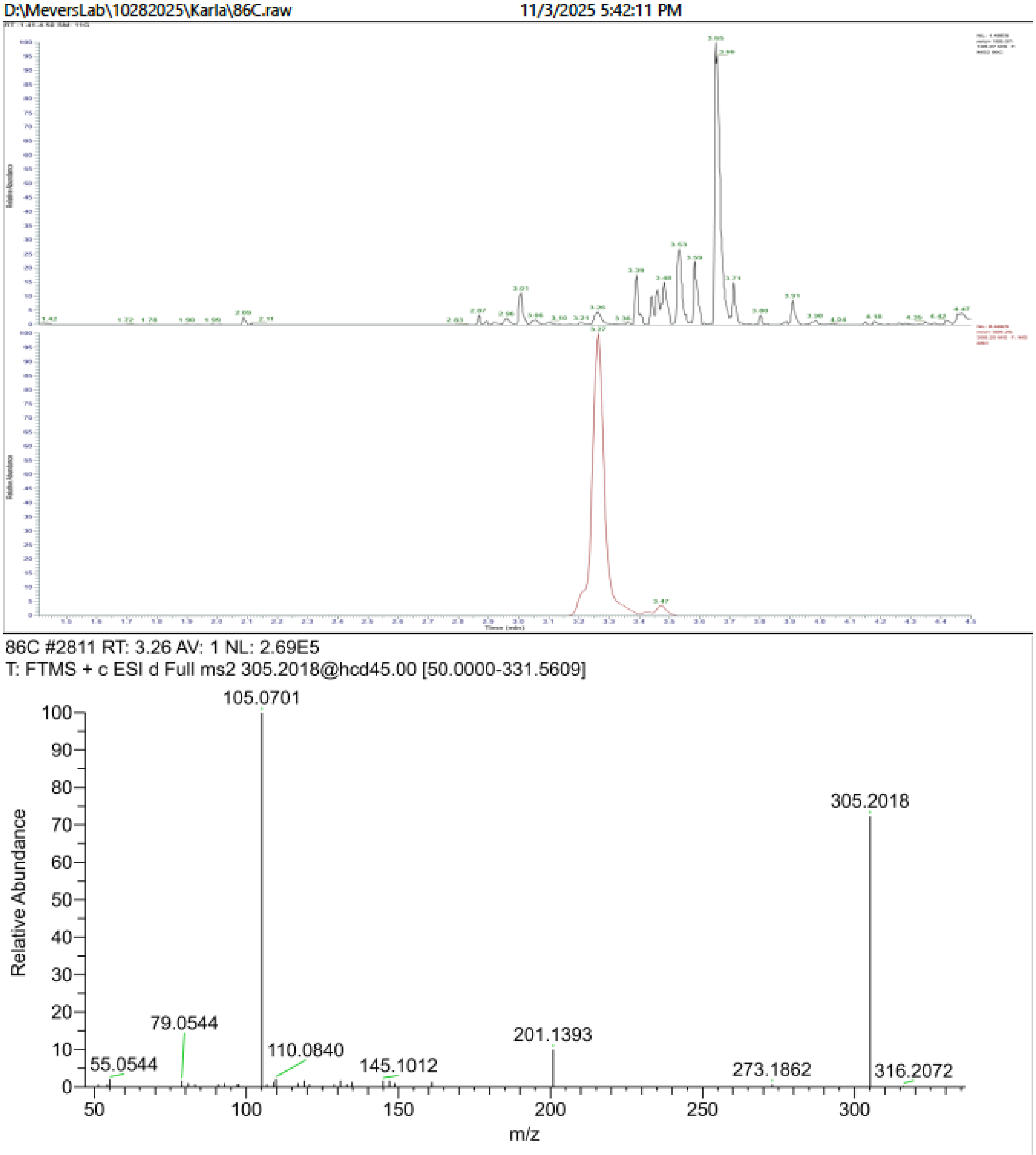

Figure S44. HR-LCMS analysis of egg collar 86C (site 3). The top chromatogram is an EIC of MSMS fragment data for 105.0700 *m/z*, the bottom chromatogram is an EIC of the parent spectra for 305.2018 *m/z*, and the spectra are for the fragmentation of 305.2041 *m/z*.

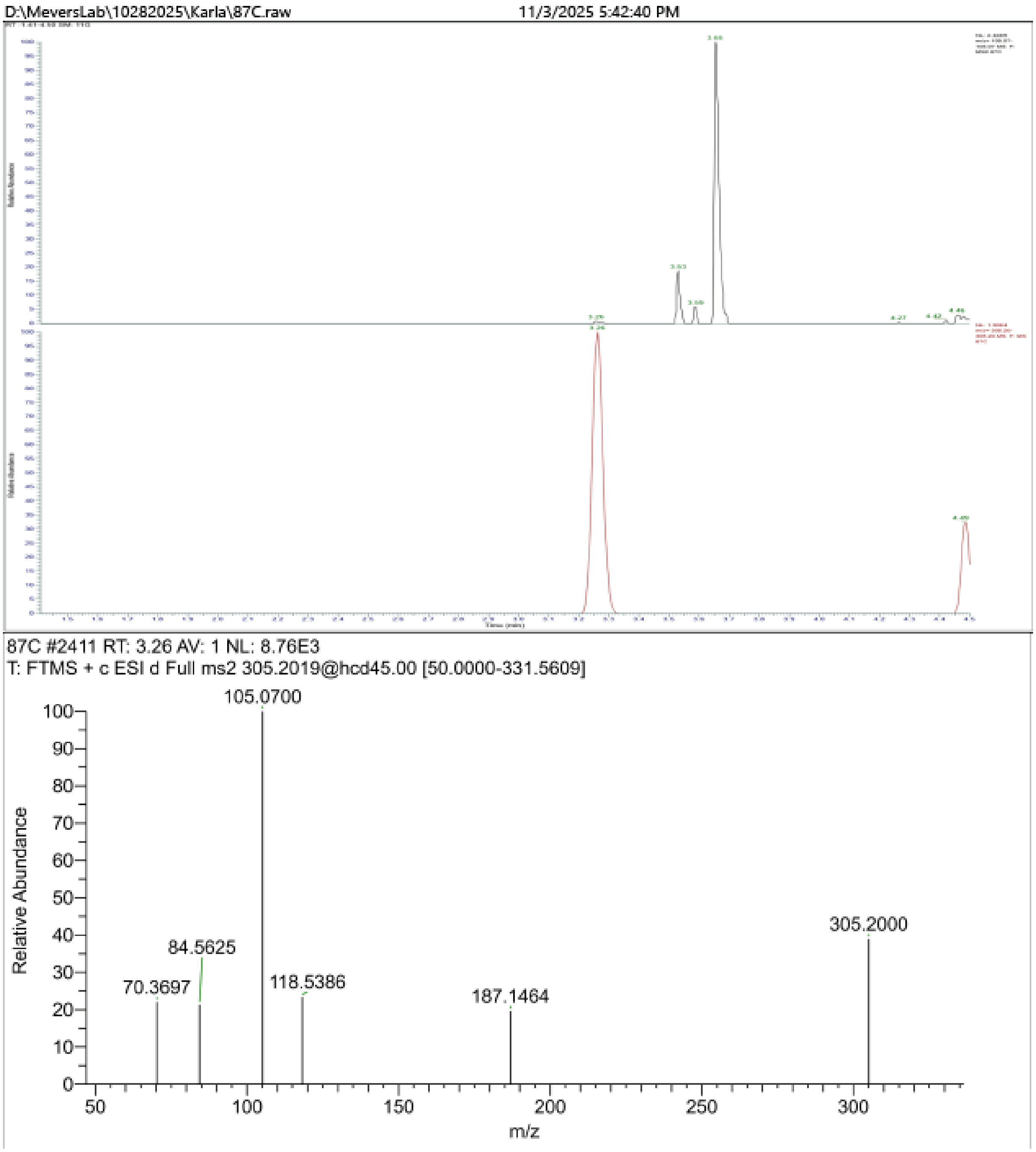

Figure S45. HR-LCMS analysis of egg collar 87C (site 3). The top chromatogram is an EIC of MSMS fragment data for 105.0700 *m/z*, the bottom chromatogram is an EIC of the parent spectra for 305.2019 *m/z*, and the spectra are for the fragmentation of 305.2041 *m/z.*

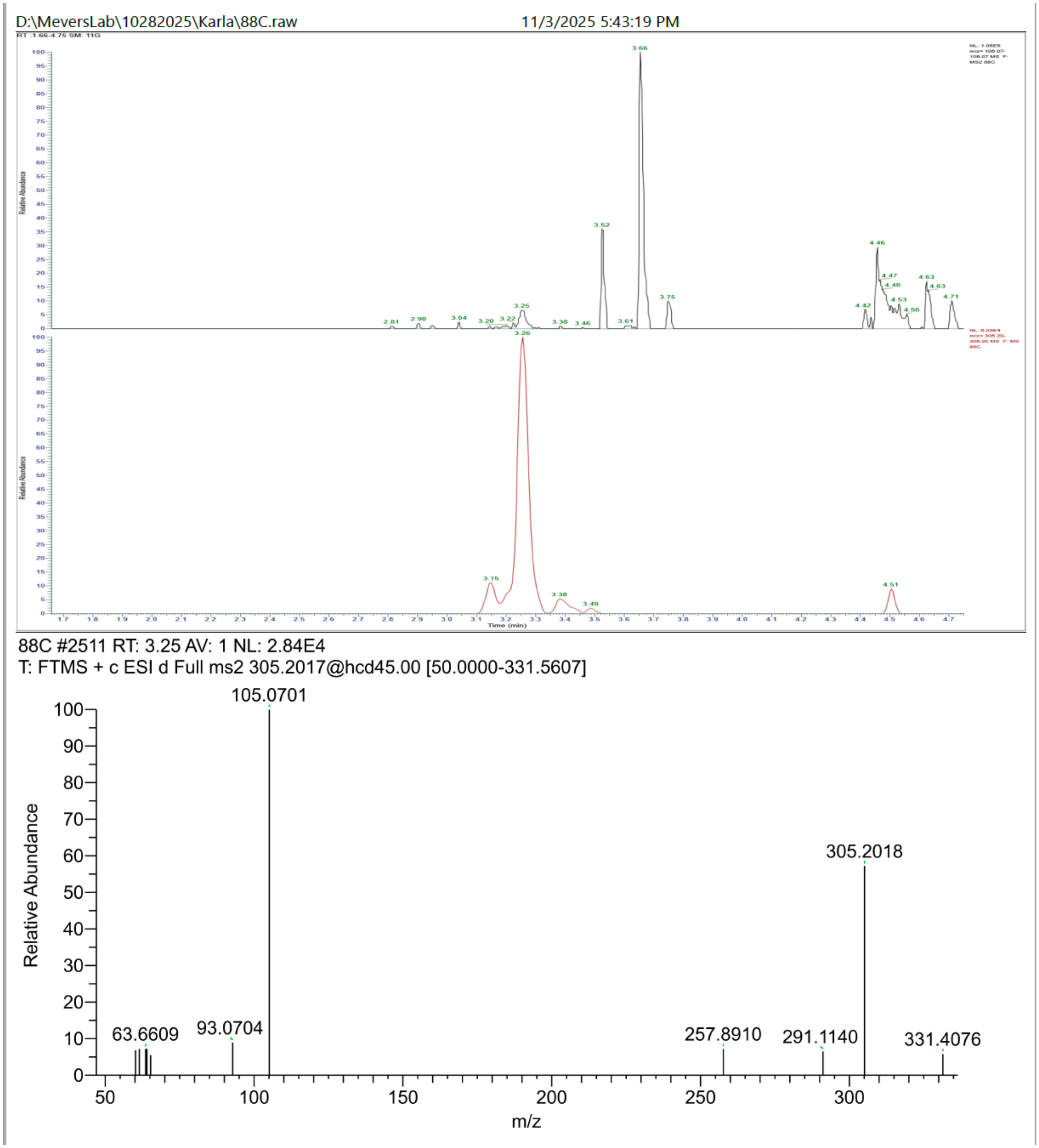

Figure S46. HR-LCMS analysis of egg collar 88C (site 3). The top chromatogram is an EIC of MSMS fragment data for 105.0700 *m/z*, the bottom chromatogram is an EIC of the parent spectra for 305.2017 *m/z*, and the spectra are for the fragmentation of 305.2041 *m/z.*

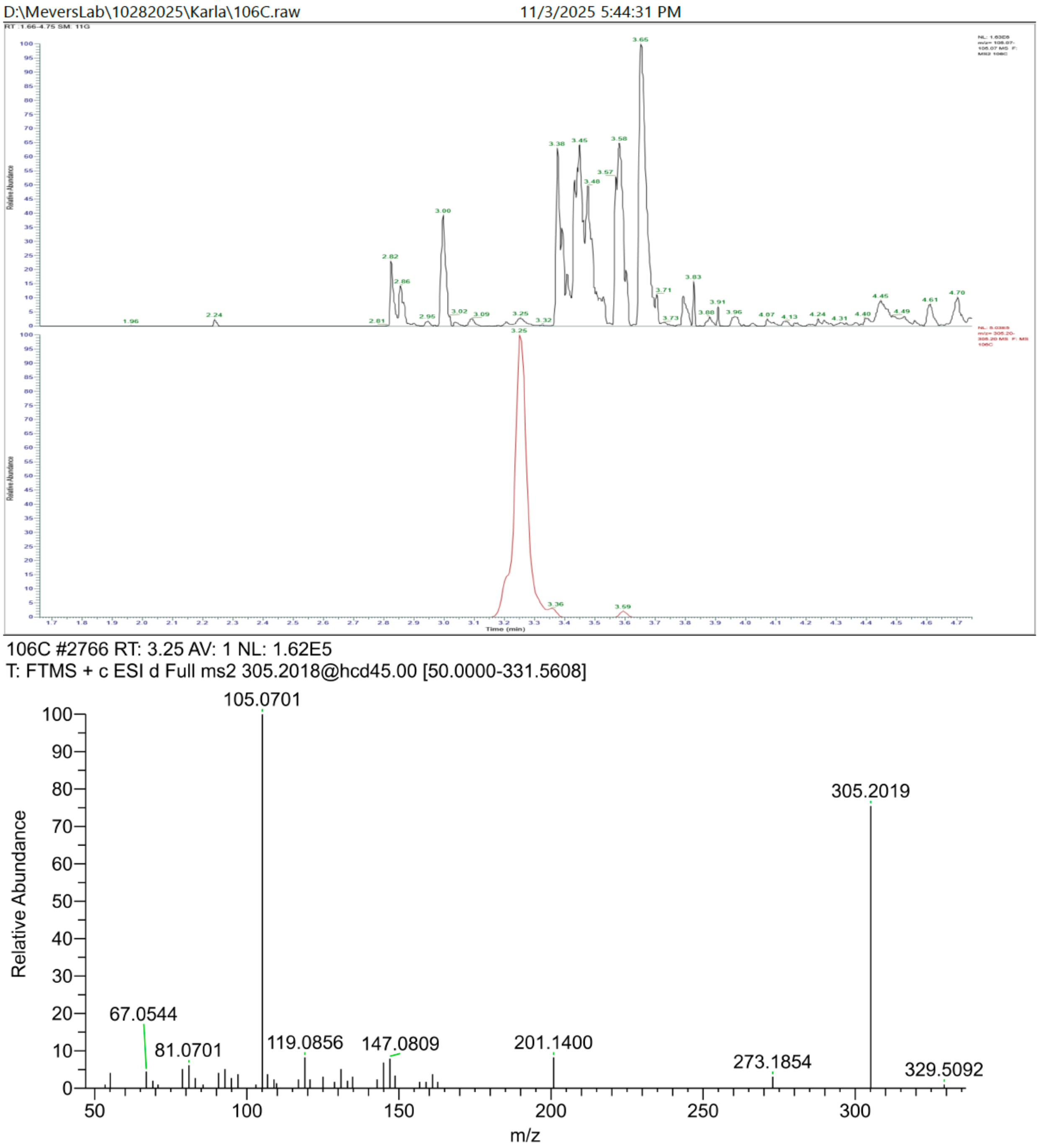

Figure S47. HR-LCMS analysis of egg collar 106C (site 4). The top chromatogram is an EIC of MSMS fragment data for 105.0700 *m/z*, the bottom chromatogram is an EIC of the parent spectra for 305.2018 *m/z*, and the spectra are for the fragmentation of 305.2041 *m/z.*

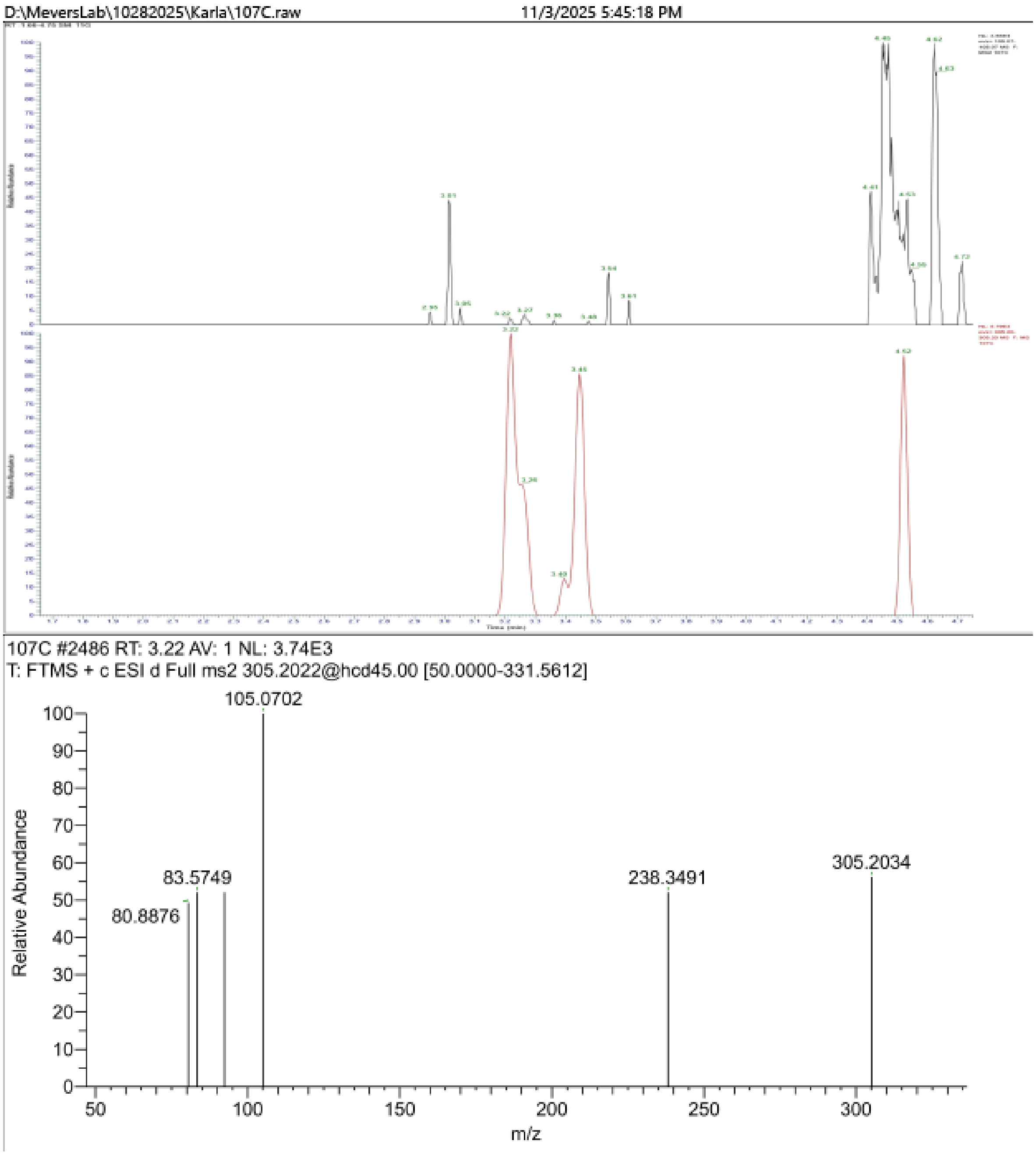

Figure S48. HR-LCMS analysis of egg collar 107C (site 4). The top chromatogram is an EIC of MSMS fragment data for 105.0700 *m/z*, the bottom chromatogram is an EIC of the parent spectra for 305.2022 *m/z*, and the spectra are for the fragmentation of 305.2041 *m/z*.

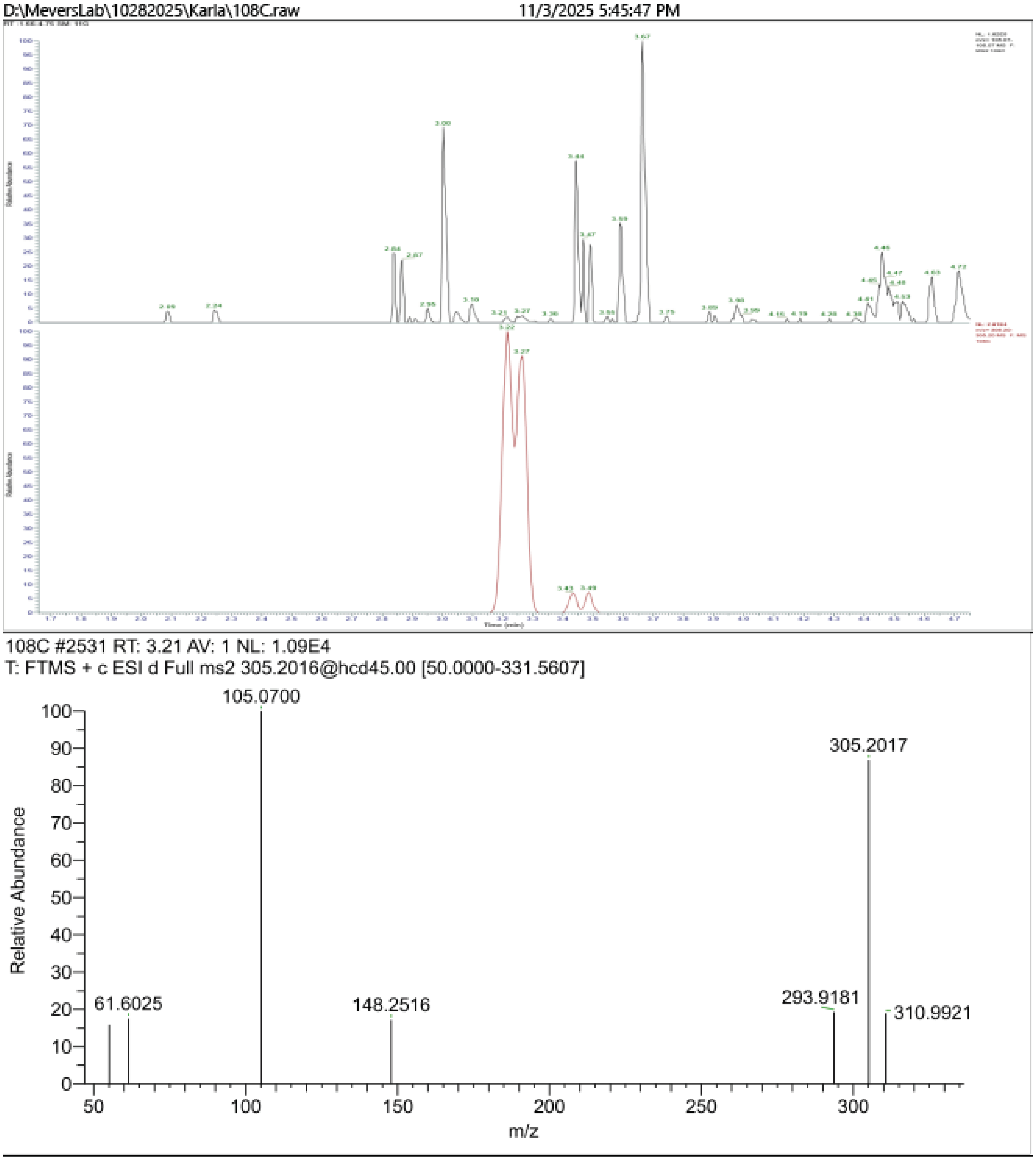

Figure S49. HR-LCMS analysis of egg collar 108C (site 4). The top chromatogram is an EIC of MSMS fragment data for 105.0700 *m/z*, the bottom chromatogram is an EIC of the parent spectra for 305.2016 *m/z*, and the spectra are for the fragmentation of 305.2041 *m/z*.

A

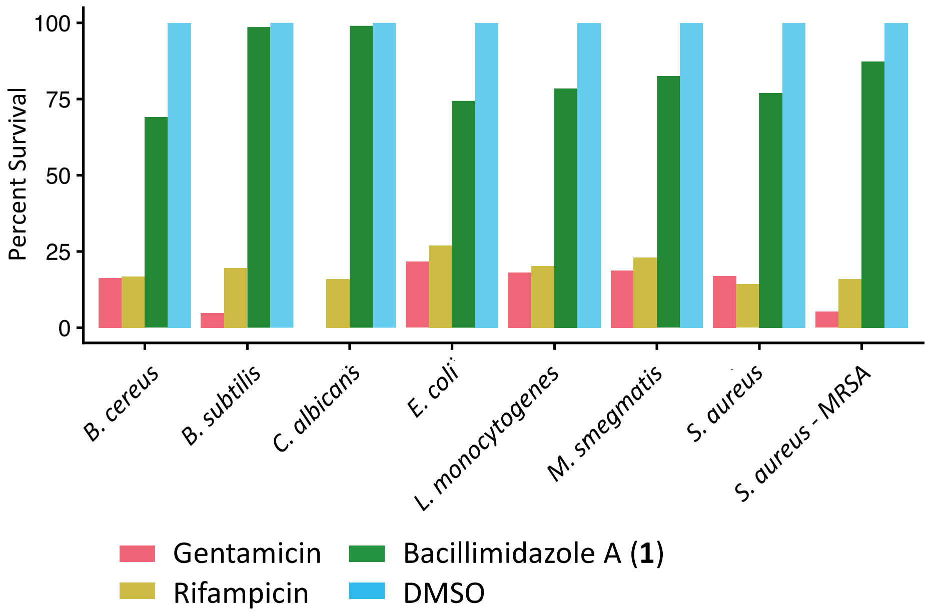

B

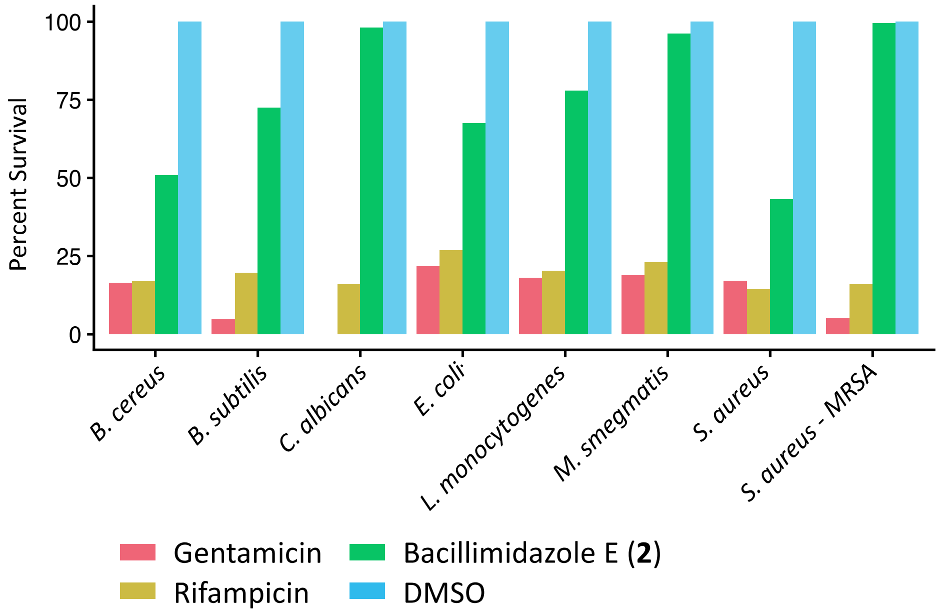

Figure S50. Percent survival at 64 µg/ml. (**A**) Bacillimidazole A (**1**) (dark green bars) and (**B**) Bacillimidazole E (**2**) (light green bars) against the human pathogens *B. cereus, E. coli, S. aureus, M. smegmatis, L. monocytogenes, S. aureus* MRSA, and *B. subtilis*. All compounds were evaluated at 64 µg/mL. *C. albicans* was evaluated against nystatin. Negative control (1% DMSO) is in light blue.

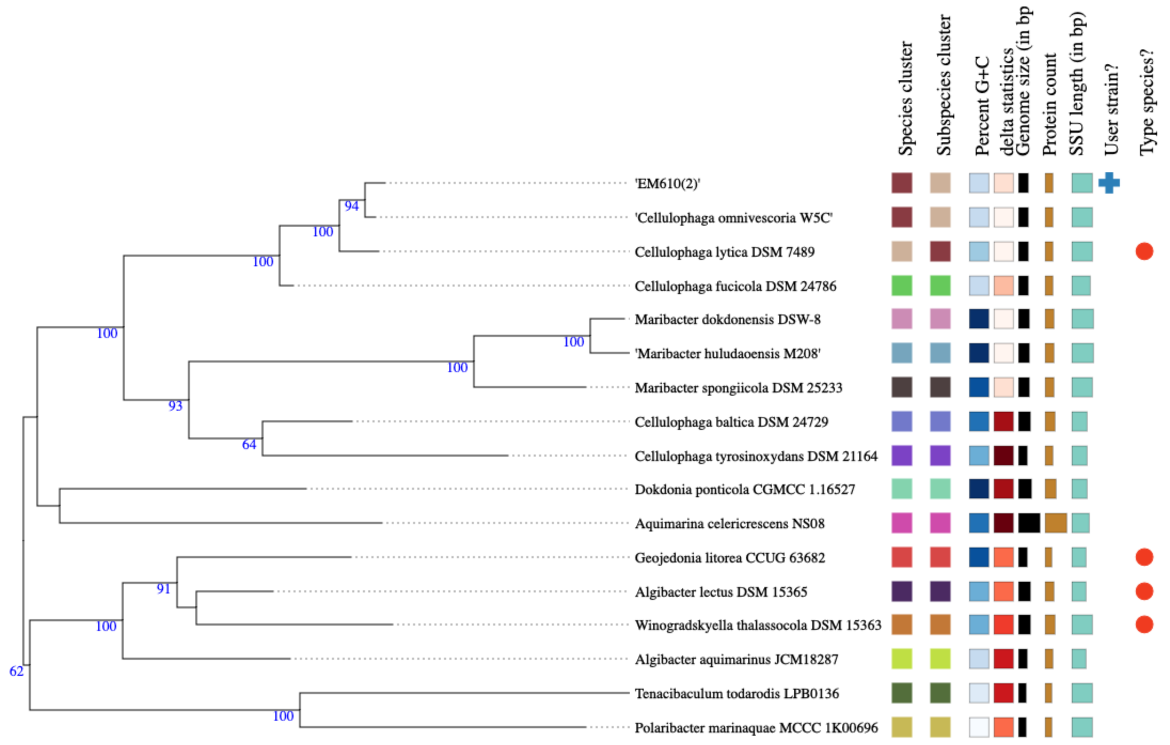

Figure S51. Genomic phylogram of C. *omniverscoria* EM610 generated by the Type Genome Server. “EM610(2)” is the query genome sequence

Supplemental Tables

**Table S1** – Binary pairings for *Flavobacteriaceae* vs. *Flavobacteriaceae.* Growth inhibition indicated with "X."		
**Resident**	**Intruder**	**Growth Inhibition**
Aquimarina sp. EM274	*Euzebyella* sp. EM328	
	Tenacibaculum sp. EM330	
	Tenacibaculum sp. EM332	
	Tenacibaculum sp. EM359	
	Tenacibaculum sp. EM375	
	Tenacibaculum sp. EM379	
	Tenacibaculum sp. EM414	
	Tenacibaculum sp. EM439	
	Tenacibaculum sp. EM445	
	Maribacter sp. EM447	
	Tenacibaculum sp. EM448	
	Aquimarina sp. EM466	
	Zunongwangia sp. EM537	
	Sufflavibacter sp. EM538	
	Olleya sp. EM584	
	Lacinutrix sp. EM585	
	Sufflavibacter sp. EM601	
	Muricauda sp. EM609	
	Cellulophaga sp. EM610	
	Winogradskyella sp. EM632	
Euzebyella sp. EM328	*Aquimarina* sp. EM274	
	Tenacibaculum sp. EM330	
	Tenacibaculum sp. EM332	
	Tenacibaculum sp. EM359	X
	Tenacibaculum sp. EM375	
	Tenacibaculum sp. EM379	X
	Tenacibaculum sp. EM414	
	Tenacibaculum sp. EM439	
	Tenacibaculum sp. EM445	
	Maribacter sp. EM447	
	Tenacibaculum sp. EM448	
	Aquimarina sp. EM466	
	Zunongwangia sp. EM537	
	Sufflavibacter sp. EM538	
	Olleya sp. EM584	X
	Lacinutrix sp. EM585	
	Sufflavibacter sp. EM601	
	Muricauda sp. EM609	
	Cellulophaga sp. EM610	
	Winogradskyella sp. EM632	

Tenacibaculum sp. EM375	*Euzebyella* sp. EM328	
	Aquimarina sp. EM274	
	Tenacibaculum sp. EM330	
	Tenacibaculum sp. EM332	
	Tenacibaculum sp. EM359	
	Tenacibaculum sp. EM379	
	Tenacibaculum sp. EM414	
	Tenacibaculum sp. EM439	
	Tenacibaculum sp. EM445	
	Maribacter sp. EM447	
	Tenacibaculum sp. EM448	
	Aquimarina sp. EM466	
	Zunongwangia sp. EM537	
	Sufflavibacter sp. EM538	
	Olleya sp. EM584	
	Lacinutrix sp. EM585	
	Sufflavibacter sp. EM601	
	Muricauda sp. EM609	
	Cellulophaga sp. EM610	
	Winogradskyella sp. EM632	
EM439 *Tenacibaculum* sp.	*Euzebyella* sp. EM328	
	Aquimarina sp. EM274	
	Tenacibaculum sp. EM330	
	Tenacibaculum sp. EM332	
	Tenacibaculum sp. EM359	
	Tenacibaculum sp. EM379	
	Tenacibaculum sp. EM414	
	Tenacibaculum sp. EM445	
	Maribacter sp. EM447	
	Tenacibaculum sp. EM448	
	Aquimarina sp. EM466	
	Zunongwangia sp. EM537	
	Sufflavibacter sp. EM538	
	Olleya sp. EM584	
	Lacinutrix sp. EM585	
	Sufflavibacter sp. EM601	
	Muricauda sp. EM609	
	Cellulophaga sp. EM610	
	Winogradskyella sp. EM632	
Tenacibaculum sp. EM448	*Euzebyella* sp. EM328	
	Aquimarina sp. EM274	
	Tenacibaculum sp. EM330	
	Tenacibaculum sp. EM332	
	Tenacibaculum sp. EM359	
	Tenacibaculum sp. EM379	

	Tenacibaculum sp. EM414	
	Tenacibaculum sp. EM445	
	Maribacter sp. EM447	
	Aquimarina sp. EM466	
	Zunongwangia sp. EM537	
	Sufflavibacter sp. EM538	
	Olleya sp. EM584	
	Lacinutrix sp. EM585	
	Sufflavibacter sp. EM601	
	Muricauda sp. EM609	
	Cellulophaga sp. EM610	
	Winogradskyella sp. EM632	
Zunongwangia sp. EM537	*Euzebyella* sp. EM328	
	Aquimarina sp. EM274	
	Tenacibaculum sp. EM330	
	Tenacibaculum sp. EM332	
	Tenacibaculum sp. EM359	
	Tenacibaculum sp. EM379	
	Tenacibaculum sp. EM414	
	Tenacibaculum sp. EM445	
	Maribacter sp. EM447	
	Tenacibaculum sp. EM448	
	Aquimarina sp. EM466	
	Sufflavibacter sp. EM538	
	Olleya sp. EM584	
	Lacinutrix sp. EM585	
	Sufflavibacter sp. EM601	
	Muricauda sp. EM609	
	Cellulophaga sp. EM610	
	Winogradskyella sp. EM632	
Sufflavibacter sp. EM601	*Euzebyella* sp. EM328	
	Aquimarina sp. EM274	
	Tenacibaculum sp. EM330	
	Tenacibaculum sp. EM332	
	Tenacibaculum sp. EM359	
	Tenacibaculum sp. EM379	
	Tenacibaculum sp. EM414	
	Tenacibaculum sp. EM445	
	Maribacter sp. EM447	
	Tenacibaculum sp. EM448	
	Aquimarina sp. EM466	
	Sufflavibacter sp. EM538	
	Olleya sp. EM584	
	Lacinutrix sp. EM585	
	Muricauda sp. EM609	

	Cellulophaga sp. EM610	
	Winogradskyella sp. EM632	
Cellulophaga sp. EM610	*Euzebyella* sp. EM328	
	Aquimarina sp. EM274	
	Tenacibaculum sp. EM330	X
	Tenacibaculum sp. EM332	
	Tenacibaculum sp. EM359	
	Tenacibaculum sp. EM379	
	Tenacibaculum sp. EM414	
	Tenacibaculum sp. EM445	
	Maribacter sp. EM447	
	Tenacibaculum sp. EM448	
	Aquimarina sp. EM466	
	Zunongwangia sp. EM537	X
	Sufflavibacter sp. EM538	X
	Olleya sp. EM584	
	Lacinutrix sp. EM585	
	Muricauda sp. EM609	
	Winogradskyella sp. EM632	

**Table S2** Genes involved in acetolactate pathway and acetolactate degradation				
**Gene**	**Present in Genome**	**Reaction Catalyzed**	**Genome Location[a]**	**Transcription Regulator[b]**
Amino acid decarboxylase (AAD)	Yes	Decarboxylation of aromatic amino acids	>2.2 million bp from BCAA operon	AsnC or AraC family regulator
Acetolactate synthase (AS)	Yes	Synthesizes 2-acetolactate from two molecules of pyruvate	BCAA operon	
Dihydroxy-acid dehydratase	Yes	Converts 2,3-dihydroxy-isovalerate to α-keto-isovalerate	BCAA operon	
Ketol-acid reductoisomerase	Yes	Converts 2,3-dihydroxy-isovalerate to 2-acetolactate	BCAA operon	
Butanediol dehydrogenase (BDH)	No	Converts 2,3-butanediol to acetoin	NA	
Acetoin (diacetyl) reductase (AR)	Yes	Converts diacetyl first to acetoin then to 2,3-butanediol	>1.1 million bp from BCAA operon	GntR family regulator
Acetoin (diacetyl) reductase (AR)	Yes	Converts diacetyl first to acetoin then to 2,3-butanediol	>2.8 million bp from BCAA operon	GntR or AraC family regulator

[a]BCAA = branched chain amino acid; [b]If annotated within 5 kb in the genome

**Table S3.** Detection of bacillimidazole A (**1**) on Florida moon snail egg masses		
Egg mass extract	Collection site	Detection of **1**
5	1	Yes
6	1	ND
7	1	ND
8	1	Yes
45	2	ND
46	2	ND
47	2	Yes
48	2	Yes
85	3	ND
86	3	Yes
87	3	Yes
88	3	Yes
105	4	ND
106	4	Yes
107	4	Yes
108	4	Yes

[a]ND = not detected

**Table S4.** Antibacterial MIC values of bacillimidazoles A and E				
	**Bacillimidazole A**	**Bacillimidazole E**	**Rifampicin**	**Gentamicin**
Olleya sp. EM584	>64 µg/mL	>64 µg/mL	<1 µg/mL	>64 µg/mL
Sufflavibacter sp. EM538	>64 µg/mL	>64 µg/mL	<1 µg/mL	>64 µg/mL
Zunongwangia sp. EM537	>64 µg/mL	>64 µg/mL	<1 µg/mL	>64 µg/mL
Bacillus cereus	>64 µg/mL	>64 µg/mL	<1 µg/mL	<1 µg/mL
Escherichia coli	>64 µg/mL	>64 µg/mL	<1 µg/mL	<1 µg/mL
Staphylococcus aureus	>64 µg/mL	>64 µg/mL	<1 µg/mL	<1 µg/mL
Pseudomonas aeruginosa	>64 µg/mL	>64 µg/mL	<1 µg/mL	<1 µg/mL
*Candida albicans**	>64 µg/mL	>64 µg/mL	----	2 µg/mL*
Bacillus subtilis	>64 µg/mL	>64 µg/mL	<1 µg/mL	<1 µg/mL
Methicillin-resistant *S. aureus*	>64 µg/mL	>64 µg/mL	<1 µg/mL	8 µg/mL
Listeria monocytogenes	>64 µg/mL	>64 µg/mL	<1 µg/mL	<1 µg/mL
Mycobacterium smegmatis	>64 µg/mL	>64 µg/mL	<1 µg/mL	<1 µg/mL

*Nystatin was used as a positive control for *C. albicans*

**Table S5.** Tools used by the SeqCenter for genome assembly and annotation		
Tool	Version	Parameters
porechop	0.2.4	Default parameters
flye	2.9.2	-asm-coverage 50 – genome-size 6000000 – nano-hq
circulator	1.5.5	All; 6-hour timeout
Bakta	1.8.1	Default parameters; db version 5.0
quast	5.2.0	Default parameters
